# The complete mitochondrial genome of okra (*Abelmoschus esculentus*): using nanopore long reads to investigate gene transfer from chloroplast genomes and rearrangements of mitochondrial DNA molecules

**DOI:** 10.1186/s12864-022-08706-2

**Published:** 2022-06-29

**Authors:** Jihan Li, Jingling Li, Yubo Ma, Lu Kou, Juanjuan Wei, Weixing Wang

**Affiliations:** 1 College of Horticulture and Landscape Architecture, Southwest University, Chongqing, 400716 China; 2 Key Laboratory of Horticulture Science for Southern Mountainous Regions from Ministry of Education, No.2 Tiansheng Road, Beibei District, Chongqing, 400716 China

**Keywords:** Okra, Mitochondrial genome, Organelle genome, *Abelmoschus esculentus*, RNA editing

## Abstract

**Background:**

Okra (*Abelmoschus esculentus* L. Moench) is an economically important crop and is known for its slimy juice, which has significant scientific research value. The *A. esculentus* chloroplast genome has been reported; however, the sequence of its mitochondrial genome is still lacking.

**Results:**

We sequenced the plastid and mitochondrial genomes of okra based on Illumina short reads and Nanopore long reads and conducted a comparative study between the two organelle genomes. The plastid genome of okra is highly structurally conserved, but the mitochondrial genome of okra has been confirmed to have abundant subgenomic configurations. The assembly results showed that okra’s mitochondrial genome existed mainly in the form of two independent molecules, which could be divided into four independent molecules through two pairs of long repeats. In addition, we found that four pairs of short repeats could mediate the integration of the two independent molecules into one complete molecule at a low frequency. Subsequently, we also found extensive sequence transfer between the two organelles of okra, where three plastid-derived genes (*psaA*, *rps7* and *psbJ*) remained intact in the mitochondrial genome. Furthermore, *psbJ*, *psbF*, *psbE* and *psbL* were integrated into the mitochondrial genome as a conserved gene cluster and underwent pseudogenization as nonfunctional genes. Only *psbJ* retained a relatively complete sequence, but its expression was not detected in the transcriptome data, and we speculate that it is still nonfunctional. Finally, we characterized the RNA editing events of protein-coding genes located in the organelle genomes of okra.

**Conclusions:**

In the current study, our results not only provide high-quality organelle genomes for okra but also advance our understanding of the gene dialogue between organelle genomes and provide information to breed okra cultivars efficiently.

**Supplementary Information:**

The online version contains supplementary material available at 10.1186/s12864-022-08706-2.

## Background

Okra (*Abelmoschus esculentus* L. Moench) belongs to the family Malvaceae and is an economic crop that is cultivated throughout the world in tropical, subtropical, and temperate regions [1]. As an annual vegetable and a medicinal source, okra has attracted much attention due to its high nutritional value and health benefits for human beings [2]. Its industrial applications mainly focus on the polysaccharides isolated from immature okra pods, which have been successfully used as emulsifiers, drug binders, edible coatings, and food packaging ingredients. Moreover, okra’s potent pharmacological effects have been verified in clinical studies, including its antidiabetic, antiobesity, and anticancer activities [3, 4]. However, low production limits the development of the okra industries. For a long time, few okra cultivars have been bred, which has contributed to yield stagnation [5]. Developing modern cultivars with significant heterosis based on cytoplasmic male sterility associated with various chimeric open reading frames in the plant mitochondrial genome (mtDNA) is common among crops. Unfortunately, no mitochondrial genome of okra has been reported thus far, which severely restricts follow-up research.

It is generally accepted that plant organelle genomes are derived from endosymbiotic bacteria [6, 7]. They have a genetic system independent of the nuclear genome, and they also established a stable regulatory mechanism with the nuclear genome in long-term evolution. Among them, plastid genomes (cpDNA) are usually structurally conserved; they have stable, double-stranded, and circular genomes that contain the core genes for photosynthesis. The combination of its rapid evolution rate and conserved genome structure make the plastid genome a good material for the phylogenomic study of plants [8–10]. cpDNA is widely used in studies of the origin of species, plant diversity and cytoplasmic evolution. In recent years, numerous plastid genomes have been assembled based on Illumina short reads, including okra [11].

However, plant mtDNA is much larger than that of other eukaryotes and it varies in size even among related species. Although mtDNA is normally depicted as a circular molecule, different structures of mtDNA molecules have also been found, including linear conformations, branched structures, and numerous smaller circular molecules [12, 13]. Thus, it is difficult for us to recover the conformation of plant mtDNA due to its redundant sequences and extensive genomic recombination [14, 15]. It has also been reported that plant mtDNA may simultaneously exist in different genome configurations, which is puzzling. Moreover, there is widespread gene transfer between organelle genomes and between organelle and nuclear genomes. For example, the mtDNA had multiple losses of ribosomal and succinate dehydrogenase genes, caused by these genes being transferred to the host cell and becoming part of the nuclear genome during plant evolution [16, 17]. Some chloroplast genes were also transferred to the nuclear genome during evolution, which is similar to the mitochondrial gene process [18, 19].

The current study sequenced and assembled the complete mitochondrial genome of okra. Based on Illumina short reads and Nanopore long reads, we deciphered the structure of okra mtDNA, whose structure is variable. These results will contribute to understanding the organelle genome evolution of okra, especially for the dialogue between the two organelle genomes, and provide information to breed okra cultivars efficiently.

## Results

### Characteristics of the mitochondrial genomes of *A. esculentus*

**Fig. 1:**
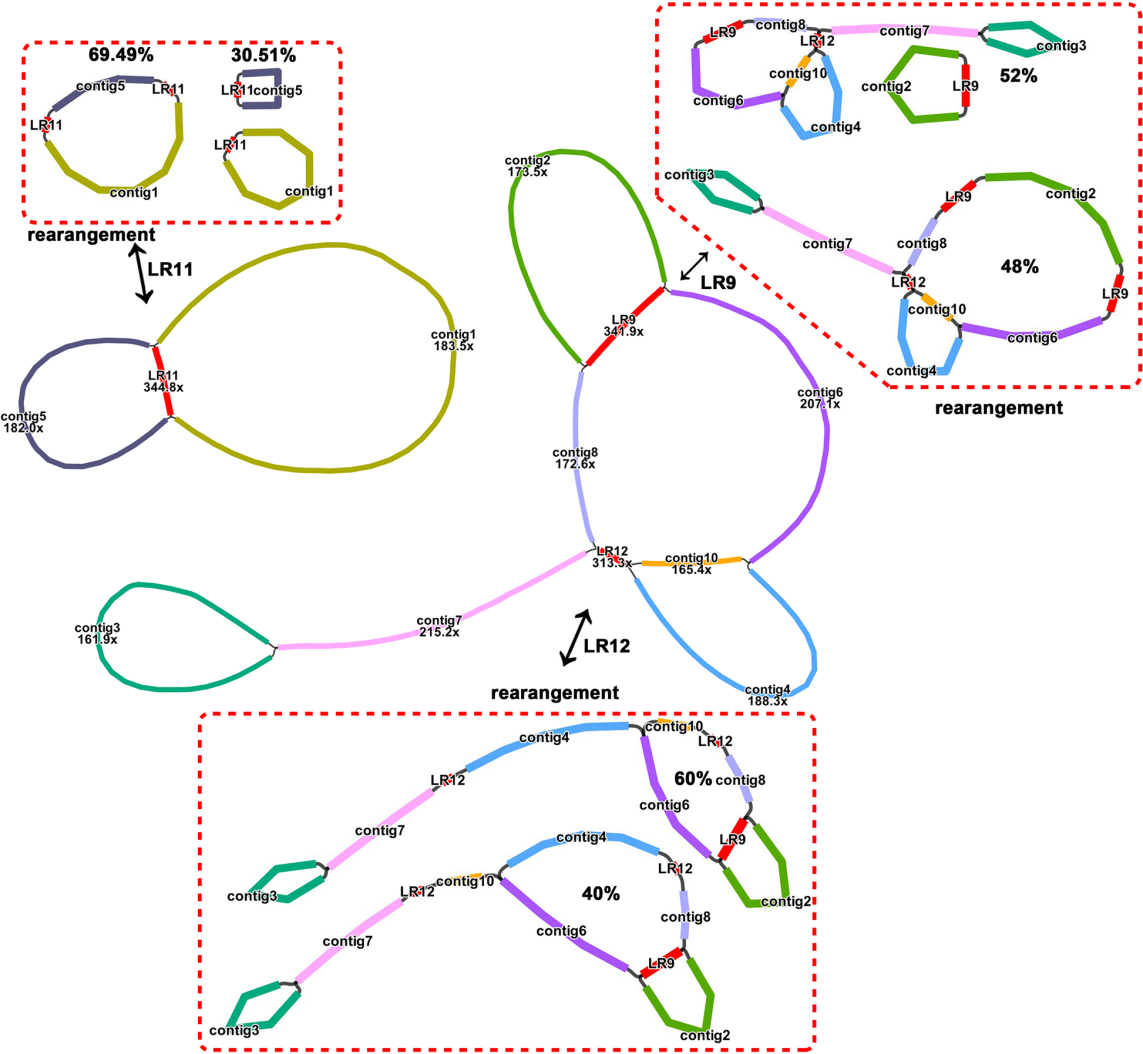
The assembly graph of the *A. esculentus* mitogenome. Each colored segment is labeled with its size and named contig/R 1–12 by rank of size. Only segment 9, 11 and 12 representations are inferred as repeats. All segment adjacencies are supported by the long reads, indicating a complex branching genomic structure. The possible structures formed by high frequency rearrangements mediated by three long repeats were drew

Initially, we obtained a complex assembly graph with 12 pairs of short repeats (SRs) and 3 pairs of long repeats (LRs) and displayed multiple paths in the Illumina-based assembly (Fig. S1A-D). We solved these repeats by artificially simulating four possible paths and making judgements based on the mapping results of long reads. As shown in Fig. S2, the structures we recovered here were supported by most long reads, and a total of 12 contigs were obtained by merging redundant nodes (Table 1). We numbered them according to their length. As shown in Fig. 1, we obtained two independent mtDNA molecules of okra, one of which had a complex multibranched conformation, but it was still a closed-loop structure (Fig. 1, above). The other one presented a typical circular molecule containing a pair of long forward repeats (LR11) (Fig. 1, below). We tried to describe molecule 1 of mtDNA (mtDNA m1) with a reasonable path, but no matter how hard we tried, it could not be reduced to a closed-loop molecule without branches.

**Table 1:** The length, depth and contained genes of each assembled contig

Sequences	Length (bp)	Contained genes	Depth
contig1	107,975	*atp5; atp4; rps12; atp9; nad2-exon3,4,5; nad4; cox2; sdh4*	183.5×
contig2	67,871	*nad1-exon1; nad7; nad9; mttB; rps4; nad5-exon3,4*	173.5×
contig3	67,036	*nad1-exon2,3; nad2-exon1,2; atp6; ccmFN; atp8; nad6; cob*	161.9×
contig4	57,782	*ccmB; ccmFC*	188.3×
contig5	52,008	*rps10; cox1; atp6; rpl16; rps3; rpl2; rpl5*	182.0×
contig6	50,848	*nad6; rps4; nad1-exon4,5; matR; atp9*	207.1×
contig7	45,477	*cob; rps14; nad5-exon1,2; atp9; rpl10; ccmC*	215.2×
contig8	18,018	*nad3; cox2; atp4*	172.6×
LR9	15,128	*rrn18; rrn5*	341.9×
contig10	11,805	*nad4L*	165.4×
LR11	7388	*rrn26; rpl14; rpl5*	344.8×
LR12	3590	*atp8*	313.3×

For the convenience of description, we processed mtDNA 1 into a linear molecule in the order of contig10 - LR12 - contig8 - LR9 - contig2 - LR9 - contig6 - contig4 - LR12 - contig7 - contig3 and processed mtDNA m2 into a circular molecule in the order of contig1 - LR11 - contig5 - LR11 - contig1. Of course, we emphasize that the treatment here is not the only form because the mitochondrial DNA configuration of plants is in dynamic transformation mediated by repeats, and the treatment here was selected since it was convenient for subsequent analysis. We mapped the short reads and long reads to the two mtDNA molecules, and the average depth was 351 × for mtDNA m1, 356 × for mtDNA 2 (short reads), and 402 × for mtDNA 1, 405 × for mtDNA 2 (long reads) (Fig. S3). Statistics of the sequencing depth showed that we obtained a gap-free genome, indicating that our assembly was of high quality.

The mtDNA contained 24 unique core genes and 10 unique variable genes (Table 2), including 5 ATP synthase genes (*atp1*, *atp4*, *atp6*, *atp8* and *atp9*), 9 NADH dehydrogenase genes (*nad1*, *nad2*, *nad3*, *nad4*, *nad4 L*, *nad5*, *nad6*, *nad7* and *nad9*), 4 cytochrome C biogenesis genes (*ccmB*, *ccmC*, *ccmFc* and *ccmFn*), 3 cytochrome C oxidase genes (*cox1*, *cox2* and *cox3*), 3 large subunit of ribosome proteins (*rpl2*, *rpl5*, *rpl10*, and *rpl16*), 4 small subunit of ribosome proteins (*rps3*, *rps4*, *rps10*, *rps12*, and *rps14*), transport membrane protein (*mttB*), maturases (*matR*), ubiquinol cytochrome c reductase (*cob*) and one respiratory gene (*sdh4*). Furthermore, all three rRNA genes were double-copy genes, including *rrn5*, *rrn18*, and *rrn26*. A total of 18 unique tRNA genes were identified based on tRNAscan-SE.

**Table 2:** Gene composition in the mitogenome of *A. esculentus*

Group of genes		Name of genes
Core genes	ATP synthase	*atp1*^2^*, atp4*^2^*, atp6*^*ψ,* 1^*, atp8*^1^*, atp9*^1^
	Cytochrome c biogenesis	*ccmB*^1^*, ccmC*^1^, ccmFc^*, 1^*, ccmFn*^1^
	Ubichinol cytochrome c reductase	*cob*^1^
	Cytochrome c oxidase	*cox1*^2^*, cox2*^1^*, cox3*^2^
	Maturases	*matR*^1^
	Transport membrane protein	*mttB*^1^
	NADH dehydrogenase	nad1^*, 1^, nad2-exon1,2^*, 1^, nad2-exon3,4,5^*, 2^*, nad3*^1^, nad4^*, 2^*, nad4L*^1^, nad5^*, 1^*, nad6*^1^ (×2), nad7^*, 1^*, nad9*^1^
Variable genes	Large subunit of ribosome	*rpl2*^2^*, rpl5*^2^*, rpl10*^1^*, rpl16*^2^
	Small subunit of ribosome	rps3^*, 2^*, rps4*^1^*, rps10*^2^*, rps12*^2^*, rps14*^1^
	Succinate dehydrogenase	*sdh4*^2^
rRNA genes	Ribosomal RNAs	*rrn5 (×2)*^1^*, rrn18 (× 2)*^1^*, rrn26 (× 2)*^2^
tRNA genes	Transfer RNAs	*trnY-GUA*^2^*, trnW-CCA (×2)*^1^*, trnS-UGA*^1^*, trnS-GCU*^2^*, trnQ-UUG*^1^*, trnP-UGG (× 2)*^1*,* 2^*, trnN-GUU*^2^*, trnM-CAU (×5)* ^1^*, trnK-UUU*^1^*, trnH-GUG*^2^*, trnG-GCC*^1^*, trnF-GAA*^2^*, trnE-UUC*^1^*, trnD-GUC (× 2)*^1*,* 2^*, trnC-GCA*^2^*, trnS-GGA*^1^*, trnL-CAA*^1^*, trnV-GAC*^2^
Plastid-derived	partial	*ndhB (×2)* ^1*,* 2^*, psbC*^1^*, psbE*^1^*, psbF*^1^*, psbL*^1^*, ycf2*^1^*, psaB*^2^*, psbM*^2^*, rps12*^2^*, rpl14*^2^
Plastid-derived	complete	*psbJ*^1^*, psaA*^2^*, rps7*^2^

^ψ^ Labeled the pseudogenes; ^*^ Labeled the genes that contain introns; ^1^ genes located in chromosome 1; ^2^ genes located in chromosome 2

**Fig. 2:**
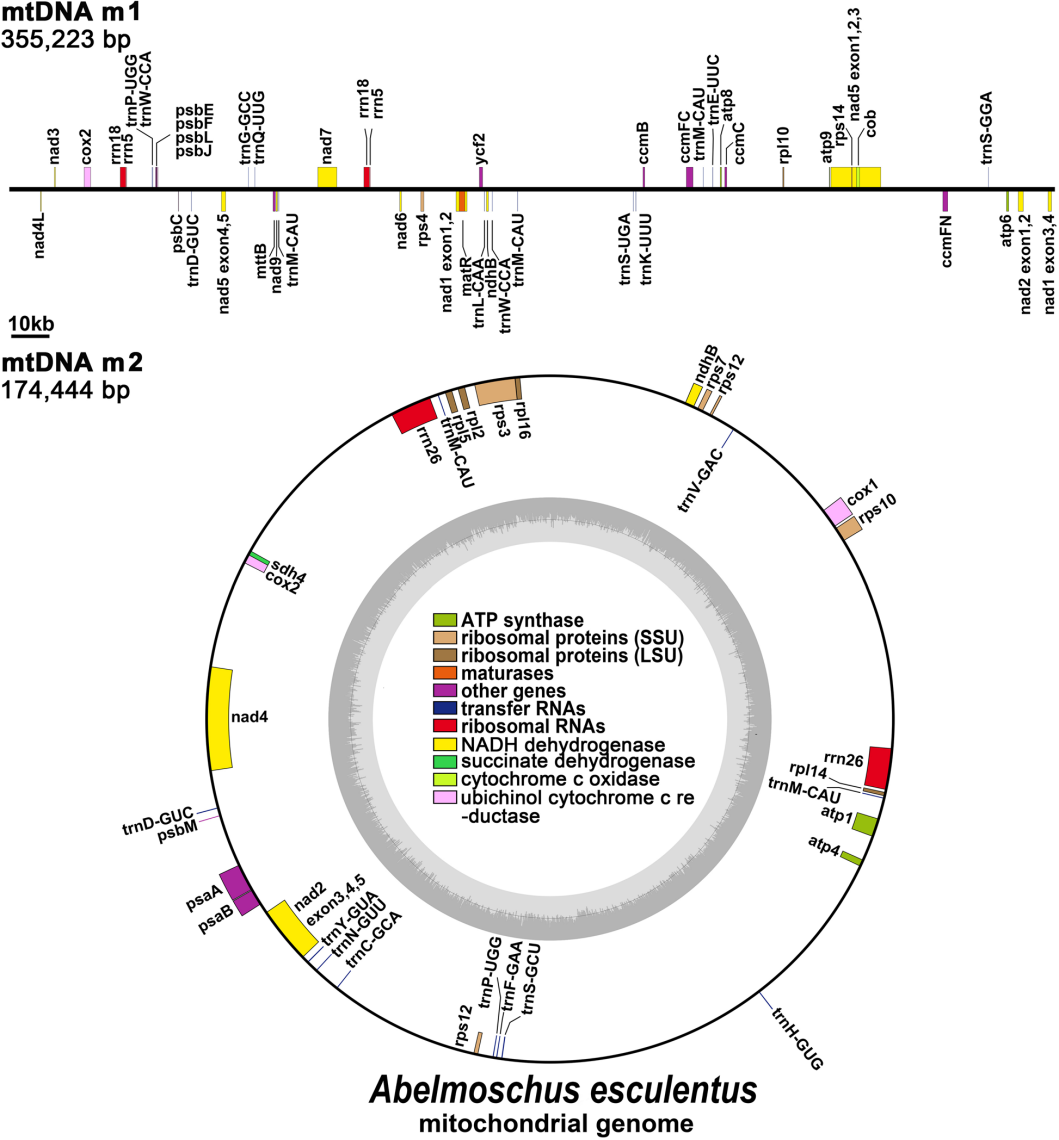
Schematic mitochondrial genome diagram of *A. esculentus*. Genes belonging to different functional groups are color-coded

Surprisingly, we also annotated many plastid genes in the mtDNA, but most of them were just fragments, such as *ndhB*, *psbC*, *psbE*, *psbF*, *psbL*, *ycf2*, *psaB*, *psbM*, *rps12*, and *rpl14*. However, we observed three intact plastid genes, *psbJ*, *psaA*, and *rps7*. This result suggested that there has been considerable sequence migration between okra cpDNA and mtDNA, accompanied by gene transfer, which will be discussed in detail below. Figure 2 shows the mtDNA genome map.

### Homologous recombination mediated by repeats

**Fig. 3:**
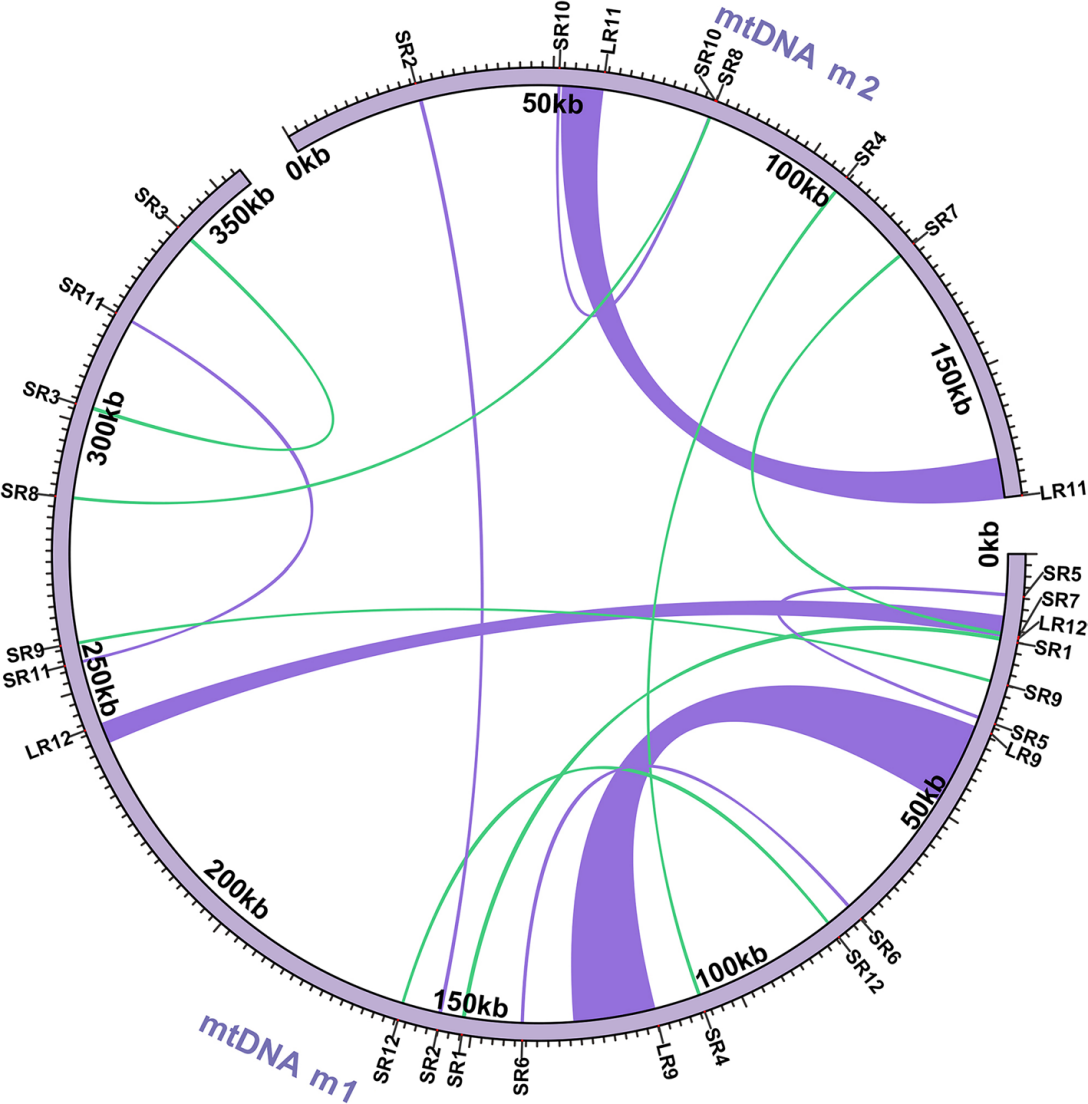
The location of repeats in the mtDNA of *A. esculentus*

We excluded false-positive repeat sequences based on Nanopore long reads (SR11, Fig. S2) and finally identified 14 pairs of repeats involved in mediated genome recombination (Fig. S2, Table 3), including the three pairs of long repeats described earlier. The remaining repeats were all short repeats, the longest being 322 bp. Their positions are shown in Fig. 3.

**Table 3:** Number and proportion of recombinant molecules mediated by 15 pairs of repeats

Repeat	Length (bp)	Location	Reads support major conformation	Reads support alternative conformation
LR9	15,128	chr1: 33,099-48,226	12 (48.00%)	13 (52.00%)
		chr1: 115,888-131,015		
LR11	7388	chr2: 59,291-51,904	41 (69.49%)	18 (30.51%)
		chr2: 174,444-167,057		
LR12	3590	chr1: 15,290-11,701	60 (60.00%)	40 (40.00%)
		chr1: 242,920-239,331		
SR1	322	chr1: 16,039-15,718	173 (91.05%)	17 (8.95%)
		chr1: 151,620-151,941		
SR2	298	chr2: 24,881-25,178	183 (98.91%)	2 (1.08%)
		chr1: 156,132-156,429		
SR3	280	chr1: 302,985-303,264	149 (91.98%)	13 (8.02%)
		chr1: 340,025-339,746		
SR4	229	chr2: 107,341-107,569	162 (98.18%)	3 (1.82%)
		chr1: 107,206-106,978		
SR5	207	chr1: 7671-7465	159 (98.15%)	3 (1.85%)
		chr1: 31,246-31,040		
SR6	204	chr1: 74,344-74,547	162 (99.39%)	1 (0.61%)
		chr1: 140,695-140,898		
SR7	201	chr2: 124,380-124,580	144 (98.63%)	2 (1.37%)
		chr1: 14,938-14,738		
SR8	169	chr2: 80,125-79,957	220 (98.21%)	4 (1.79%)
		chr1: 285,880-286,048		
SR9	146	chr1: 23,908-24,053	193 (97.97%)	4 (2.03%)
		chr1: 258,829-258,684		
SR10	131	chr2: 51,068-51,198	178 (98.34%)	3 (1.66%)
		chr2: 79,866-79,996		
SR11	128	chr1: 255,127-255,254	189 (100.00%)	0 (0.0%)
		chr1: 320,910-321,037		
SR12	125	chr1: 79,449-79,325	207 (99.04%)	2 (0.96%)
		chr1: 163,440-163,564

In our case of okra, three pairs of long repeats mediated recombination with high frequency. The proportions of the two different isomers mediated by the three pairs of long repeats were 48% vs. 52% (LR9), 69.49% vs. 30.510% (LR11), and 60% vs. 40% (LR12). Figure 1 shows the possible conformation mediated by the three long repeats. Both LR9 and LR11 served as mediators for further separation of the two independent molecules. In this case, four independent molecules could exist at the same time. The frequency of LR9-mediated recombination was slightly higher than the main configuration, i.e., 13 long reads covered the LR9 repeats and supported contig2 forming an independent molecule with LR9, while 12 long reads supported it as part of mtDNA m1. However, the length of repeats was so long that the number of long reads available for reference was statistically limited. For the long repeats, the true ratio was probably closer to equal. However, for the remaining short repeats, the major conformation was clearly dominant in the mitochondria. The alternative conformation generated by the short repeats was less than or close to 2%, except for SR1 and SR3, which were nearly 8% (Table 3). Due to the shorter length of these repeats, we were able to map more long reads and obtained a ratio closer to the actual situation.

Notably, the two repeated units of four pairs of short repeats (SR2, SR4, SR7, and SR8) were found to be located on the two molecules. They were able to participate in the recombination of the two molecules at a low frequency, giving them a chance to merge into one complete molecule.

### Intracellular gene transfer (IGT) of *A. esculentus* organelle genomes

**Fig. 4:**
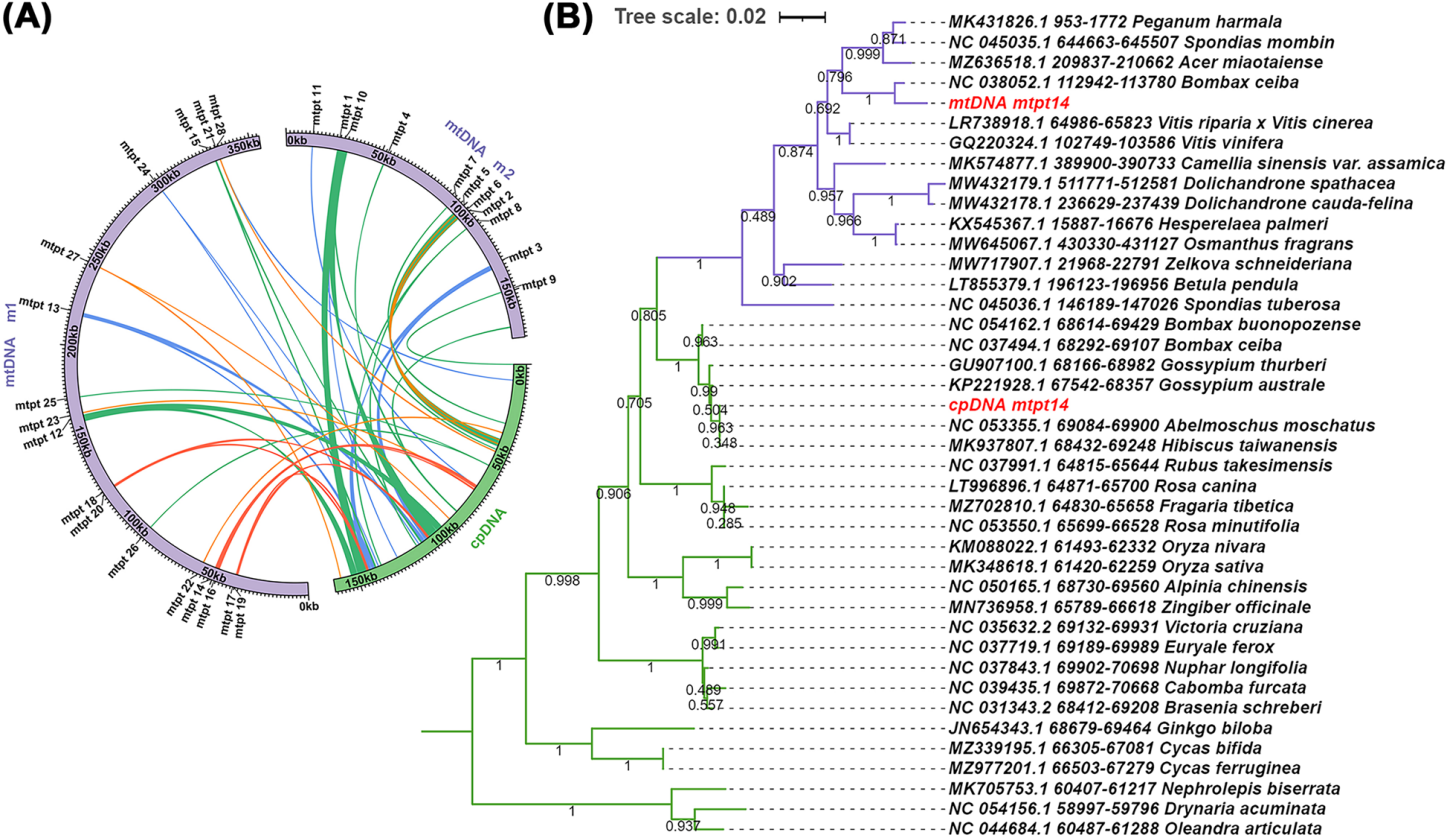
Schematic of homologous sequences identified among the two organelle genomes. **A** The blue arcs represent the mtpts with 100% similarity, the green arcs represent the mtpts has similarity be-tween 90 to 100%, the red arcs represent the mtpts has similarity between 80 to 90%, and the orange arcs represent the mtpts has similarity between less than 80%. **B** Phylogenetic tree base on the partial of mtpt14 sequences identified in cp DNA and mt DNA. The purple branches represent origin from mt DNA and green branches represent origin from cp DNA. The mt DNA mtpt14 and cp DNA mtpt14 are extracted from okra organelle genomes. The other sequences are downloaded from NCBI, the accession number and position are shown in the label

The assembly and annotation of cpDNA revealed that the cpDNA obtained here was almost identical to that previously reported. Therefore, the cpDNA was extremely conserved for okra. In the previous annotation of the organelle genome, we found the presence of gene residues from plastids in the mitochondrial genome, meaning that there was much sequence migration between the two organelles. Here, we searched for homologous sequences among the two organelle genomes based on the BLASTn program to identify potential gene transfer events. A total of 28 homologous sequences were identified (Fig. 4A and Table 4), among which 6 were over 1000 bp in length, and the longest was 5142 bp. The total length of these repeats was 21,231 bp, including 13,340 bp in the repeat region of cpDNA and 311 bp in the repeat region of mtDNA. Therefore, a total of 34,571 bp were homologous with cpDNA, accounting for 21.19% of it, and a total of 21,542 bp were homologous with mtDNA, accounting for 4.07% of it.

**Table 4:** Plastid homologous sequences identified in mitogenome (MTPTs) of *A. esculentus*

Fragments	Aligned length (bp)	Mitogenome		Plastome		contained genes
		Start	End	Start	End	
mtpt1^2^	5142	32,524	27,383	101,579	106,720	*ndhB-exon1; rps7; rps12-exon2,3; trnV-GAC; rrn16S-fra*
		27,383	32,524	144,473	149,614	
mtpt2^2^	3833	103,040	99,223	40,982	44,814	*psaA; psaB-fra*
mtpt3^2^	2039	135,795	133,757	142,208	144,246	*trnI-GAU; rrn16S-fra*
		133,757	135,795	106,947	108,985	
mtpt4^2^	311	53,039	53,349	86,170	86,480	*rpl14-fra*
		168,192	168,502	86,170	86,480	
mtpt5^2^	126	100,379	100,254	41,419	41,544	*psaB-fra*
mtpt6^2^	126	102,603	102,478	43,643	43,768	*psaA-fra*
mtpt7^2^	84	94,520	94,437	32,029	32,112	*trnD-GUC*
mtpt8^2^	82	110,099	110,018	136,381	136,461	*trnN-GUU*
		110,018	110,099	114,732	114,812	
mtpt9^2^	74	149,018	148,945	1	74	*trnH-GUG*
mtpt10^2^	39	29,944	29,906	46,790	46,828	*/*
		29,944	29,906	126,842	126,880	
mtpt11^2^	36	13,796	13,761	69,098	69,133	*/*
mtpt12^1^	3114	162,783	159,670	150,223	153,335	*ycf2-fra*
		159,670	162,783	97,858	100,970	
mtpt13^1^	1596	217,672	216,077	140,003	141,598	*trnA-UGC-exon2; rrn23S-fra*
		216,077	217,672	109,595	111,190	
mtpt14^1^	1410	50,483	49,125	68,905	70,261	*psbJ; psbL-fra; psbF-fra; psbE-fra*
mtpt15^1^	440	330,367	329,936	111,355	111,790	*rrn23S-fra*
		329,936	330,367	139,403	139,838	
mtpt16^1^	447	48,748	48,339	71,113	71,558	*trnW-CCA; trnP-UGG*
mtpt17^1^	430	38,777	38,363	143,683	144,102	*rrn16S-fra*
		38,363	38,777	107,091	107,510	
mtpt18^1^	430	121,566	121,152	143,683	144,102	*rrn16S-fra*
		121,152	121,566	107,091	107,510	
mtpt19^1^	318	38,220	37,908	144,237	144,546	*rrn16S-fra*
		37,908	38,220	106,647	106,956	
mtpt20^1^	318	121,009	120,697	144,237	144,546	*rrn16S-fra*
		120,697	121,009	106,647	106,956	
mtpt21^1^	153	332,554	332,706	47,948	48,089	*trnS-GGA*
mtpt22^1^	157	57,548	57,393	37,270	37,416	*psbC-fra*
mtpt23^1^	126	164,006	164,129	71,090	71,212	*trnW-CCA*
mtpt24^1^	70	297,221	297,152	113,130	113,199	*rrn23S-fra*
		297,152	297,221	137,994	138,063	
mtpt25^1^	79	172,737	172,659	56,243	56,321	*trnM-CAU*
mtpt26^1^	73	91,588	91,516	56,249	56,321	*trnM-CAU*
mtpt27^1^	77	243,859	243,783	91,951	92,025	*trnI-CAU*
		243,783	243,859	159,168	159,242	
mtpt28^1^	30	332,703	332,674	8468	8497	*trnS-GCA-fra*

^1^ labeled mtpts located in mtDNA molecule 1, ^2^ labeled mtpts located in mtDNA molecule 2, “-fra” labeled the gene fragments

We then extracted and annotated these homologous sequences. Most of these fragments migrated from cpDNA to mtDNA, except that a few tRNA genes were highly similar in sequence and we could not determine the direction of migration. Thus, we called these mitochondrial plastid sequences (MTPTs). In addition to tRNAs and rRNAs, fragments homologous to plastid PCGs were identified on 8 MTPTs, including mtpt1 (*ndhB*-exon1; *rps7*; and *rps12*-exon2,3), mtpt2 (*psaA; psaB*), mtpt4 (*rpl14*), mtpt5 (*psaB*), mtpt6 (*psaA*), mtpt12 (*ycf2*), mtpt14 (*psbJ; psbL; psbF;* and *psbE*) and mtpt22 (*psbC*). We noted that three genes were still intact in the mtDNA sequences, including *rps7*, *psaA*, and *psbJ*. The first two genes were 100% similar in sequence.

We noted that 7 of these MTPTs failed to distinguish from the chloroplast homologous sequences during assembly. Most of these fragments were highly similar to cpDNA sequences. For example, mtpt1, the longest homologous fragment, had only 5 mismatches to corresponding cpDNA sequences (Table S1). With the help of long reads, it was confirmed that they migrated from chloroplasts and were integrated into the mtDNA (Fig. S4).

mtpt14 from cpDNA differs from its mtDNA sequence. On mtpt14, in addition to *psbJ*, we also found three gene fragments (*psbL*, *psbF* and *psbE*), which might have been transferred to the mitochondria together as a whole and showed varying degrees of pseudogenization during the evolution of the mitochondrial genome, but only the *psbJ* gene was relatively intact in sequence (Fig. S5). The results of phylogenetic analysis based on the mtpt14 homologous sequences showed that the mitochondrial sequences were clustered into a group (Fig. 4B). We looked closely at the sequence and found that some SNPs and Indels were shared only in mtDNA (Supplementary file 2). This indicated that this homologous sequence has undergone different evolutionary processes along with the two organellar genomes.

### RNA editing sites in the PCGs of organelle genomes

**Fig. 5:**
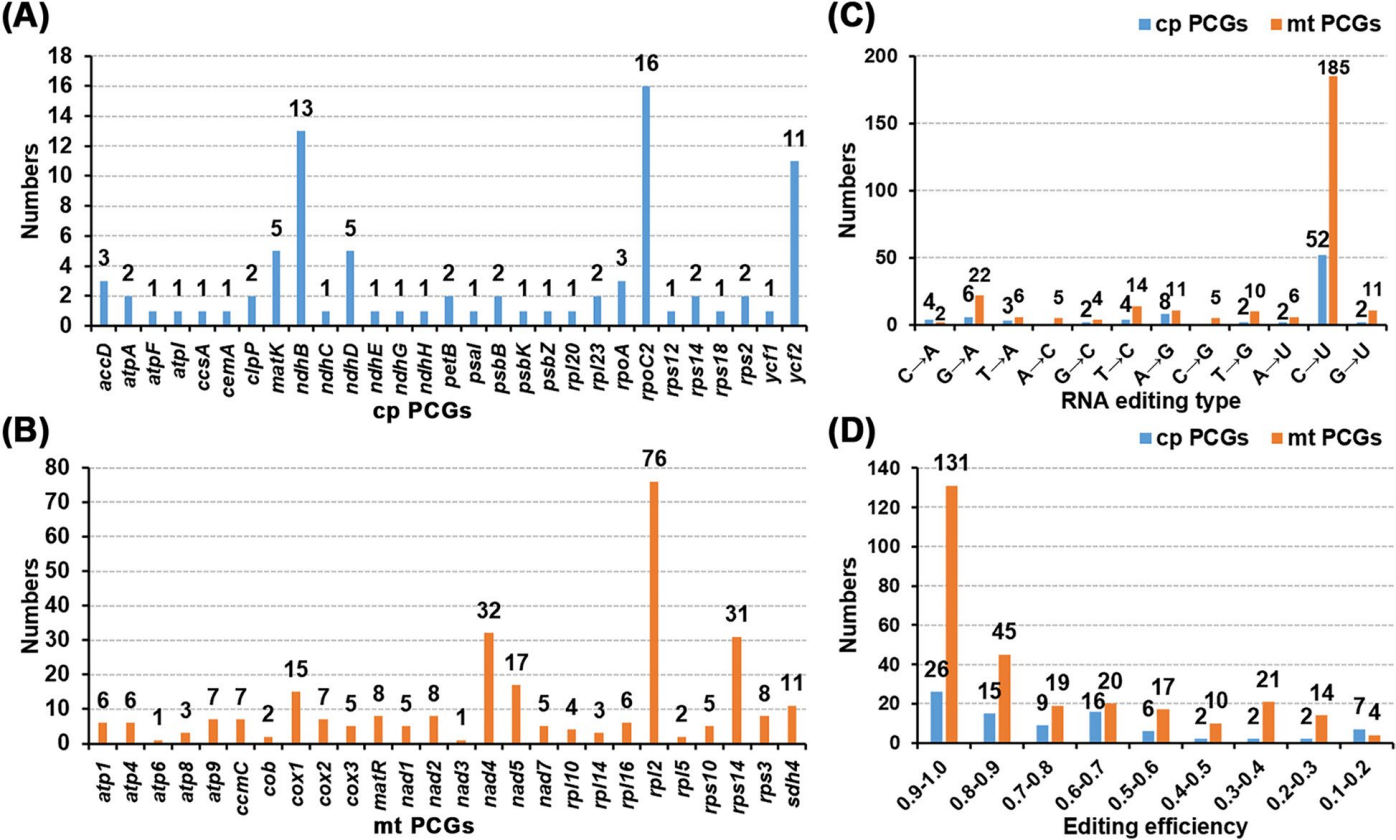
Characteristics of the RNA editing sites identified in PCGs of A. esculentus organelle genomes. **A** The number of RNA editing sites identified in each PCGs of plastid genome; **B** The number of RNA editing sites identified in each PCGs of mitochondrial genome; **C** RNA editing type and their number identified in all PCGs. **D** RNA editing efficiency

RNA editing events are common in plant mitochondrial genomes [20]. This includes single base substitutions and the addition of bases to complete the initiation or termination codon [20–22]. In this study, we focused on RNA editing events in the PCGs of okra organelle genomes. A total of 29 plastid PCGs (Fig. 5A) and 26 mitochondrial PCGs (Fig. 5B) were identified as having undergone RNA editing events. However, the total number of RNA editing events identified in plastid PCGs was only 85 (Table S2) compared with 281 in mitochondrial PCGs (the raw data were uploaded on Figshare, the link is 10.6084/m9.figshare.19608789, Table S3). In plastid PCGs, *rpoC2* had the most RNA editing sites, followed by *ndhB* and *ycf2* with 16, 13 and 11, respectively. In mitochondrial PCGs, *rpl2* had the most RNA editing sites, with 76, followed by *ndh4* and *rps14*, both more than 30.

Furthermore, we identified a total of 12 different types of RNA editing, all of which were detected in mitochondrial PCGs. However, A to C and C to G editing types were not identified in plastid PCGs (Fig. 5C). Among them, C to U editing was the most common in both plastids and mitochondria (52 and 185, respectively). Most of the other types were less than 10. In terms of editing efficiency, most PCGs of plastids and mitochondria had an editing efficiency above 80% (Fig. 5D), and the number of low-frequency editing events was relatively low. A total of 46.62% (131) of editing events in mitochondria had an editing efficiency of more than 90%.

However, it should be noted that the RNA editing sites identified here might be incomplete, and we found that multiple mitochondrial PCGs had low gene expression, such as *ccmB*, *ccmFN*, *mttB*, *nad4 L*, *nad9*, etc. These PCGs lacked adequate coverage, which might be due to their low expression levels or a small amount of sequencing data.

## Discussion

Homologous recombination mediated by repeats is almost universal in plant mitochondrial genomes [23–25]. In addition to acting as a good mediator for genome recombination, these repeats also greatly increase the size of mtDNA [26, 27]. In the assembly of the okra mitochondrial genome, we also found repeats with recombination activity. We confirmed that 14 of these repeats could mediate genome recombination based on long reads. However, it must be noted that some potential repeats involved in recombination have not been discovered. It was previously found in *Nymphaea colorata* [28] that the two units of repeats do not need to be 100% similar. Therefore, some sequences with low similarity might also mediate genome recombination.

It has been reported that the size of the repeats is closely related to the frequency of recombination [20], namely, the frequency of recombination mediated by short repeats tends to be lower than that mediated by long repeats, the isomers mediated by which were closer to equal proportions. For large repeats (e.g., typical inverted repeats observed in cpDNA), it was thought previously that they mediate SSC region recombination in equal proportions [29]. The long repeats we found in the okra mitochondrial genome also have a high frequency of recombination. For short repeats, they all had low recombination frequency, which is consistent with those previously reported [28, 30].

The mtDNA m1 of okra has a branching structure. In terms of coverage, both contig4 and contig10 were single-copy, and both ends overlapped with LR12 and contig6. However, for contig 6, its other end only overlapped with LR9. Therefore, there were two different paths (contig6-contig10-LR12 and contig6-contig4-LR12). However, it was not a repeat region (Fig. 1). In our previous assembly based on Oxford Nanopore data, these two paths’ results were also obtained. Another node in question was contig7, which overlapped both ends of contig3 on one side, but the other side only overlapped with LR12, thus creating an awkward structure. This result suggested that the mtDNA of okra most likely has a multibranched conformation or that there could be different mtDNA molecules in different copies of the mitochondrial genome, which explained why we could not assemble a circular molecule. The polymorphisms in the conformation of the plant mitochondrial genome has always puzzled us. As a previous study on lettuce showed, plant mtDNA should be presented as multiple sequence units showing their variable and dynamic connection rather than as circles [12, 13]. Our results also supported the representation that mtDNA should be considered a dynamic genome. In okra’s case, at least, this structure is a more complete description of a mtDNA.

Horizontal gene transfer (HGT) has been widely discussed, especially in parasitic plants. Adam [31] reported host-to-parasite horizontal gene transfer (hpHGT) events of several genes. These host-derived plastidial genes were found in the mitochondrial genome of the parasite plant *Aphyllon epigalium*. However, in addition to hpHGT, intracellular gene transfers (IGTs) have also been widely reported and have been an interesting topic. Gene transfer between cpDNA, mtDNA and nuclear genomes had previously been identified. Many plastidial genes have been reported to be found in mitochondria. For example, the plastid-derived *rpl32* gene has been transferred into the nucleus of the subfamily Thalictroideae [32]. The *atpI* gene in the *Aeginetia indica* mitogenome was acquired from another angiosperm’s chloroplast genome [33], and IGT events of multiple ribosomal proteins were also found in *Geranium* [34]. Here, we found three complete genes in the mitogenomes that migrated from the cpDNA of okra, including *psaA*, *rps7* and *psbJ*, as well as several plastid-derived gene fragments. However, as previously reported, these genes transferred from plastids might not function in mitochondria, and they might undergo pseudogenization as the mitochondrial genome evolves [33, 35]. In our study, a typical example was the *psbJ* gene, which has a total length of 123 bp, but the two genes we annotated in plastids and mitochondria had 12 mismatches, accounting for nearly 10% of the total length (Table S4). We mapped transcriptome data to these two *psbJ* genes, all of which were transcripts of the plastid *psbJ* gene, and no transcriptional evidence was detected for the mitochondrial *psbJ* gene. Based on the phylogenetic analysis of mtpt14, we hypothesized that mtpt14 may be an ancient fragment of plastid migration, and this migration event was shared by many plant mitochondrial genomes. However, with the evolution of mitochondrial genomes, some plant lineages may have lost this gene cluster derived from plastids. Furthermore, considering the difference in the evolutionary rate between mtDNA and cpDNA, it is difficult to determine exactly when this sequence was transferred from plastids to mitochondria. More mtDNA sequencing should be performed in the future to address this question.

## Conclusions

In this study, we completed the sequencing and assembly of okra organelle genomes and obtained a high-quality organelle genome. Although the chloroplast genome of okra has been previously published, we obtained the complete mitochondrial genome, which enabled us to make a comprehensive comparison between the organelle genomes of okra, thus providing a broader perspective for studying gene transfer between mitochondria and plastid. The use of a mixture of long reads and short reads made it possible to accurately assemble the plant mitochondrial genomes with limited homology. At the same time, the long reads also facilitated the structural analysis of these complex organelle genomes, which enabled us to describe the organelle genomes, especially the dynamic transformation of the plant mitochondrial genome, more intuitively than the previous limited description. Deciphering the organelle genome of okra can provide invaluable information for future investigations of the genome structure and mechanism of replication of Malvales organelle genomes.

## Materials and methods

### Plant materials

The okra (*A. esculentus*) seeds were planted and germinated in small plastic pots and grown in a temperature incubator held at 25 °C with a 16-hr/8-hr light/dark cycle for 2 weeks. We collected well-grown young leaf tissue for DNA extraction. The remaining parts were preserved in the Herbarium of Southwest University, and the voucher number was SWU-QK01.

### DNA extraction and sequencing

Total genomic DNA was extracted by using the CTAB method [36]. The same DNA sample was used for Illumina sequencing and Oxford Nanopore sequencing. For Illumina sequencing, the experimental procedures were carried out according to the standard protocol provided by Illumina: the DNA library with an insert size of 350 bp was constructed using the NEBNext® library building kit [37] and was sequenced by using the HiSeq Xten PE150 sequencing platform at BioMaker (Wuhan, China). Sequencing produced 15.62 Gb of clean data (52.29 Mb clean reads). Clean data were obtained by using Trimmomatic [38]. For Oxford Nanopore sequencing, gTube was used to break the genomic DNA into approximately 8 kb on average, and long-read sequencing followed the protocol in the SQK-LSK109 genomic sequencing kit (ONT, Oxford, UK). The purified library was loaded into an R9.4 Spot-On Flow Cell (ONT) and Oxford Nanopore GridION × 5 sequencing were carried out for 48 h at BioMaker (Wuhan, China). In total, 9.71 Gb of sequence reads (1,454,069 reads) were obtained. The clean read N50 was 17.40 kb.

### Assembly and annotation of organelle genomes

First, we used GetOrganelle v1.7.5.1 [39] to complete the assembly of the plastid genome (cpDNA) by referring to the parameters recommended by the author. For the mitochondrial genome (mtDNA) assembly, the Oxford Nanopore long reads were assembled into contigs using Nextdenovo with default parameters. Mitochondrial contigs were identified in each draft assembly by the BLASTn program [40] using the mitochondrial genome sequences of *Gossypium arboretum* (accession number: NC_035073.1) as a reference. As a result, there were two self-loop and three linear contig candidates with abundant matched hits. We then assembled the long reads using Smartdenovo [41] with default parameters, obtaining three self-loop and three linear candidate contigs. During our assembly of the mitochondrial genomes, we found that several pairs of repeats might mediate genome recombination, since these repeats were thought to have multiple connections during SPAdes [42] assembly. This result puzzled us, and we thought there might be a complex configuration of the mtDNA that interfered with the assembly. However, given the large number of foreign DNA fragments inserted into the mtDNA of plants, these multiple connections might be “false-positive-positives”; they might not be real, just artificial structures. Subsequently, we performed a de novo assembly of Illumina short-read data using SPAdes and obtained a preliminary draft mtDNA, a complex multibranched and closed-loop conformation (Fig. S5A). We then manually simplified the graph using Bandage [43] software by removing the chloroplast- and nuclear-derived nodes (Fig. S5B). During this process, some chloroplast nodes were retained, as they might be mitochondrial plastid DNA (MTPT). Thereafter, previous long-read assembly results were used to eliminate the interference of the repeats to restore the true mtDNA structure as much as possible. Finally, with the help of long reads, we obtained two independent molecules, and they were the dominant configurations of okra mtDNA (Fig. S5C).

The cpDNA was annotated using CPGAVAS2 [44] with the reference of 2544 plastomes. The two molecules of mtDNA were annotated using GeSeq [45] with the reference mtDNA of *G. arboretum* (accession number: NC_035073.1). The protein-coding genes (PCGs) were manually checked and edited using Apollo [46] if there were some problems. The genome map was drawn using OGDRAW [47]. All transfer RNA genes were confirmed by using tRNAscan-SE [48] with default settings.

### Detection of genome recombination

In a previous mtDNA assembly, we found multiple repeats present in the draft mitochondrial genome (LR9, LR11, LR12 and SR1-SR12). Although we obtained the mitochondrial genome using long-read data, our assembly might only represent the dominant configuration of okra mtDNA. Given the structural variability of mtDNA, these repeats may be involved in mediating genome recombination, resulting in nondominant configurations. We mapped long reads to these repeats to detect any evidence of genome recombination. Specifically, for each repeat, there were two paths representing the major conformations (m1 and m2) and two paths representing the secondary conformations (s1 and s2), and we mapped the long reads to the 4 conformations. The flanking region of each repeat was also extended by an additional 1 kb region to ensure that the mapped long reads completely spanned the repeat region, and only reads long enough to completely cover the repeat sequences were counted as reads supporting this configuration. Two paths supporting the same conformation (m1 and m2, s1 and s2) only counted the number of reads of the one with the largest number. Particularly, for the nondominant configuration, we carefully checked each long read using Tablet [49] to eliminate ambiguous reads.

### Analysis of intracellular gene transfer (IGT)

Due to the lack of a published nuclear genome for okra, only the two organelle genomes could be used for the identification of intracellular sequence migration at present. To identify the homologous sequences that might be transferred among the organelles, we compared the cpDNA of okra with the mtDNA using the BLASTn program with the following parameters: -evalue 1e-5, −word_size 9, −gapopen 5, −gapextend 2, −reward 2, −penalty-3, and -dust no. The BLASTn results were visualized using TBtools [50]. The identified transferred DNA fragments were also extracted according to their genome position and then annotated using GeSeq. We noted that most of these homologous sequences in the mitochondrial and chloroplast genomes, known as MTPTs, were not 100% similar in sequence. The plastid-derived and mitochondria-derived proteins could be distinguished in the Kmer-based assembly. However, 6 MTPTs were found during mitochondrial assembly (Fig. S5B), which could not be distinguished by Kmer-based assembly. We also used long reads to verify migration events for these MTPTs. When there was a long read supporting an MTPT flanked by mtDNA, this could indicate that this MTPT has been absorbed and integrated by the mitochondrial genome.

### Identification of RNA editing sites

To identify RNA editing sites that occur at protein-coding genes (PCGs) in organelle genomes, we downloaded three sets of transcriptome data from NCBI (SRR15808319; SRR15808320; SRR15808321). In addition, to exclude the interference of natural variation, we also downloaded the WGS data (SRR5812498) to search for single nucleotide polymorphisms (SNPs) located in organelle PCGs. We mapped all of the downloaded data to protein-coding sequences extracted from the organelle genomes to identify RNA editing sites and SNPs. Here, we calculated the base composition and coverage of each site of each PCG in BAM files based on a custom script. For high-copy chloroplast PCGs, a minimum of 20× coverage and 10% or more read support were required to be considered RNA editing sites or SNPs. For mitochondrial PCGs of low copy number and low expression, the coverage was relaxed to 10×. Finally, sites that excluded SNPs were considered high-quality RNA editing sites in PCGs of the organelle genome of okra.

### Phylogenetic inference

We conducted a BLAST search on the NCBI website for the regions homologous to mtpt14 in okra. We found that these plastid-derived homologous fragments were present in multiple mitochondrial genomes and were approximately 850 bp in length (Fig. S4). We downloaded these aligned sequences and added additional homologous sequences from cpDNA of other plant lineages to construct a phylogenetic tree. The corresponding nucleotide sequences were aligned using MAFFT (v7.450) [51]. Bayesian inferences (BI) analysis was performed using MrBayes (v3.2.6) [52] with the Markov chain Monte Carlo method for 200,000 generations and sampling trees every 100 generations. The first 20% of trees were discarded as burn-in, with the remaining trees being used for generating a consensus tree.

## Supplementary Information

**Additional file 1.**

**Additional file 2.**

## Data Availability

The assembled organelle genome sequences have been deposited in NCBI (https://www.ncbi.nlm.nih.gov/) with accession number: OL348387.1 (mtDNA 1); OL348388.1 (mtDNA 2); OL348389.1 (cpDNA). All data generated in this study are available at the corresponding author upon reasonable request.

## Abbreviations

PCG: Protein-coding genes
DNA: Deoxyribonucleic acid
SNPs: Single Nucleotide Polymorphism
tRNA: Transfer RNA
rRNA: Ribosomal RNA
cpDNA: Chloroplast genome
mtDNA: Mitochondrial genome
MTPT: Mitochondrial plastid DNA sequence
BI: Bayesian inferences
SNP: Single nucleotide polymorphisms
IGT: Intracellular Gene Transfer
HGT: Horizontal gene transfer
