# Supplementary material for: The complete mitochondrial genome of okra (Abelmoschus esculentus): using nanopore long reads to investigate gene transfer from chloroplast genomes and rearrangements of mitochondrial DNA molecules
Source: BMC Genomics. 2022 Jun 29;23:481. doi: 10.1186/s12864-022-08706-2 (PMC9245263; doi:10.1186/s12864-022-08706-2)
Supplement: Supplementary file 1 — Additional file 1. [file 12864_2022_8706_MOESM1_ESM.pdf]

Figure S1. Comparison of okra mitochondrial genome assembly based on Illumina data and Oxford nanopore data. **A.** Original assembly results based on Illumina data. **B.** Graph after manual removed the plastid- and nuclear-derived nodes. We assembled The Oxford nanopore data using Nextdenovo and compared the assembly results to Illumina assembly by BLASTn program. The nodes colored with blue, orange, and yellow were correspond to the three linear contigs assembled by long-reads, and the nodes colored with dark purple and light purple were correspond to the two looped contigs. In the smartdenovo-based assembly result, the difference is that the blue nodes can form a circular structure. The red nodes represent the repeats, and the green nodes represents the MTPTs with high coverages. **C.** The repeats were numbered by length. **D.** Graph after solved the repeats and validated the migration of MTPTs.

A

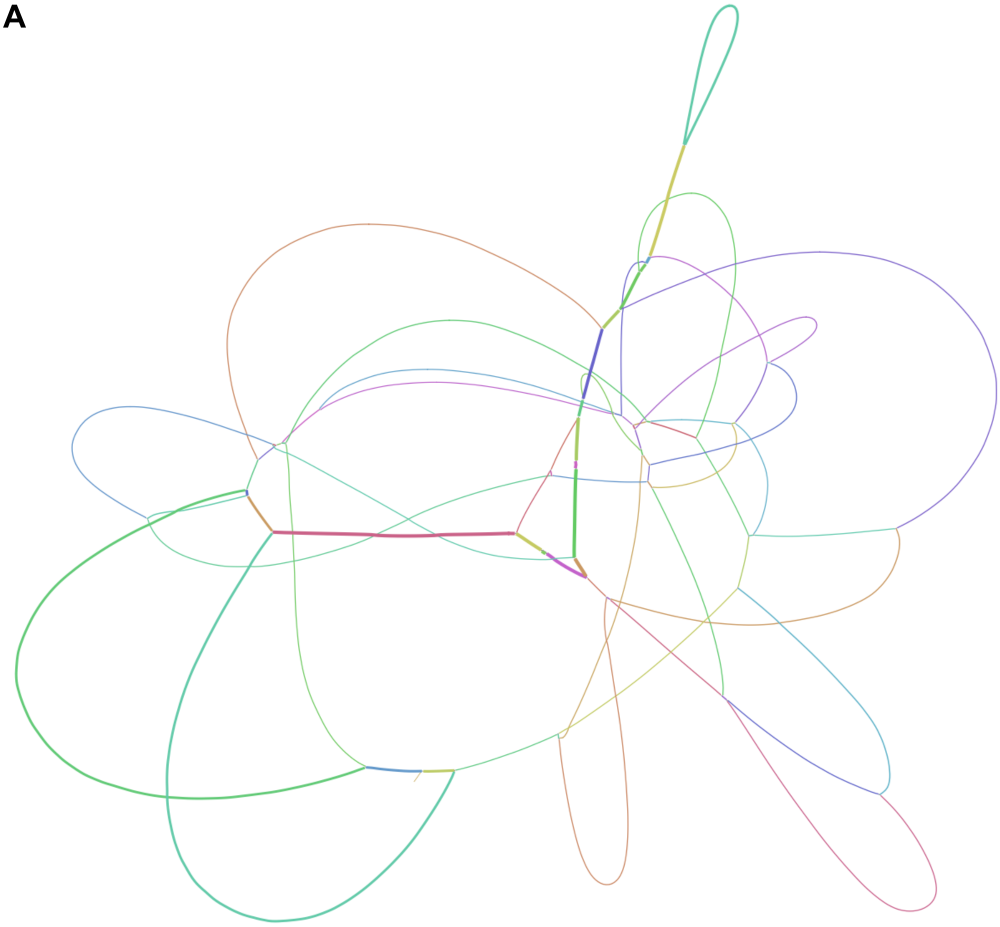

B

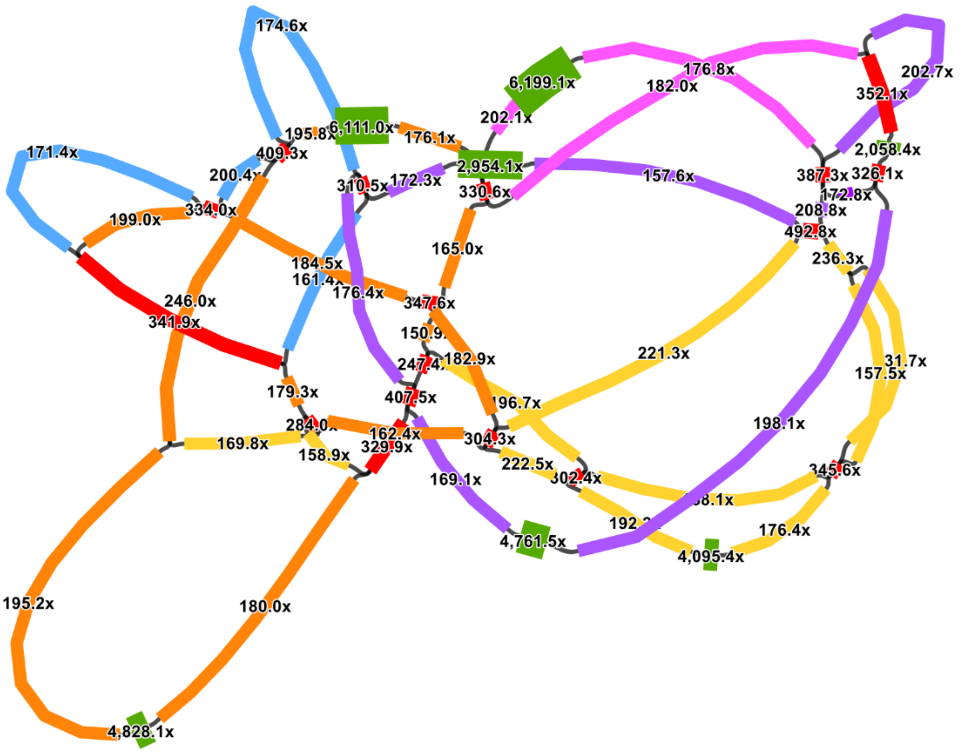
174.6x
171.4x
195.8x
6,111.0x
176.1x
409.3x
200.4x
334.0x
199.0x
310.5x
172.3x
2,954.1x
330.6x
6,199.1x
202.1x
182.0x
176.8x
157.6x
352.1x
202.7x
2,058.4x
387.3x
326.1x
172.8x
208.8x
492.8x
236.3x
165.0x
184.5x
176.4x
347.6x
246.0x
341.9x
182.9x
221.3x
31.7x
157.5x
179.3x
407.5x
196.7x
162.4x
304.3x
169.8x
158.9x
329.9x
198.1x
222.5x
302.4x
345.6x
169.1x
176.4x
4,761.5x
4,095.4x
195.2x
180.0x
4,828.1x

C

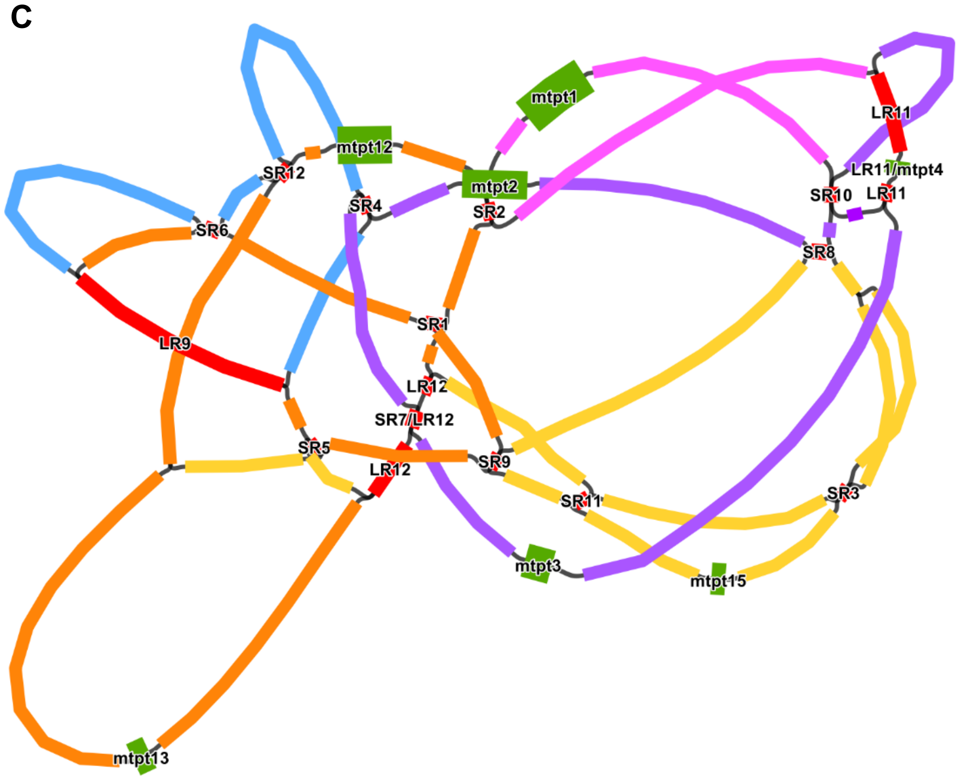
mtpt1
mtpt12
SR12
SR4
mtpt2
SR2
SR6
LR11
LR11/mtpt4
SR10
LR11
SR8
SR1
LR9
LR12
SR7/LR12
SR5
SR9
LR12
SR11
SR3
mtpt3
mtpt15
mtpt13

D

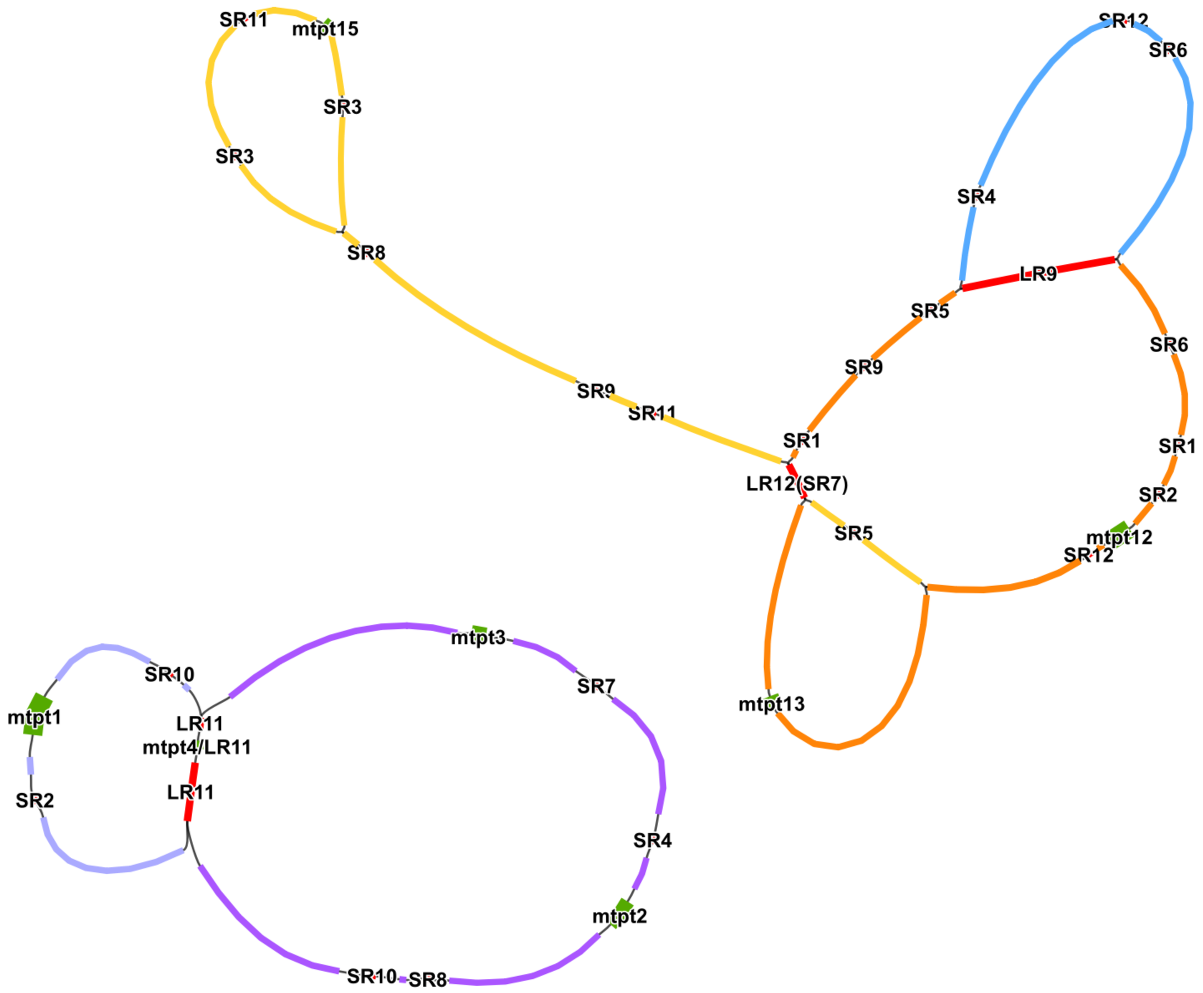
SR11
mtpt15
SR3
SR3
SR8
SR9
SR11
SR12
SR6
SR4
LR9
SR5
SR6
SR9
SR1
SR1
LR12(SR7)
SR2
SR5
mtpt12
SR12
mtpt13
SR10
mtpt3
SR7
mtpt1
LR11
mtpt4/LR11
LR11
SR2
SR4
mtpt2
SR10
SR8

Figure S2. Using long-reads to resolved the repeats. For each repeat, there were two paths representing the major conformations (m1 and m2) and two paths representing the secondary conformations (s1 and s2), **A-O** were LR9, LR11, LR12 and SR1 to SR12 respectively. Some reads were marked with dark blue to conveniently distinguish if they spanned the repeats, and only one of them was marked when reads exceeded 100. Two paths supporting the same conformation (m1 and m2, s1 and s2) only count the path with most reads.

A

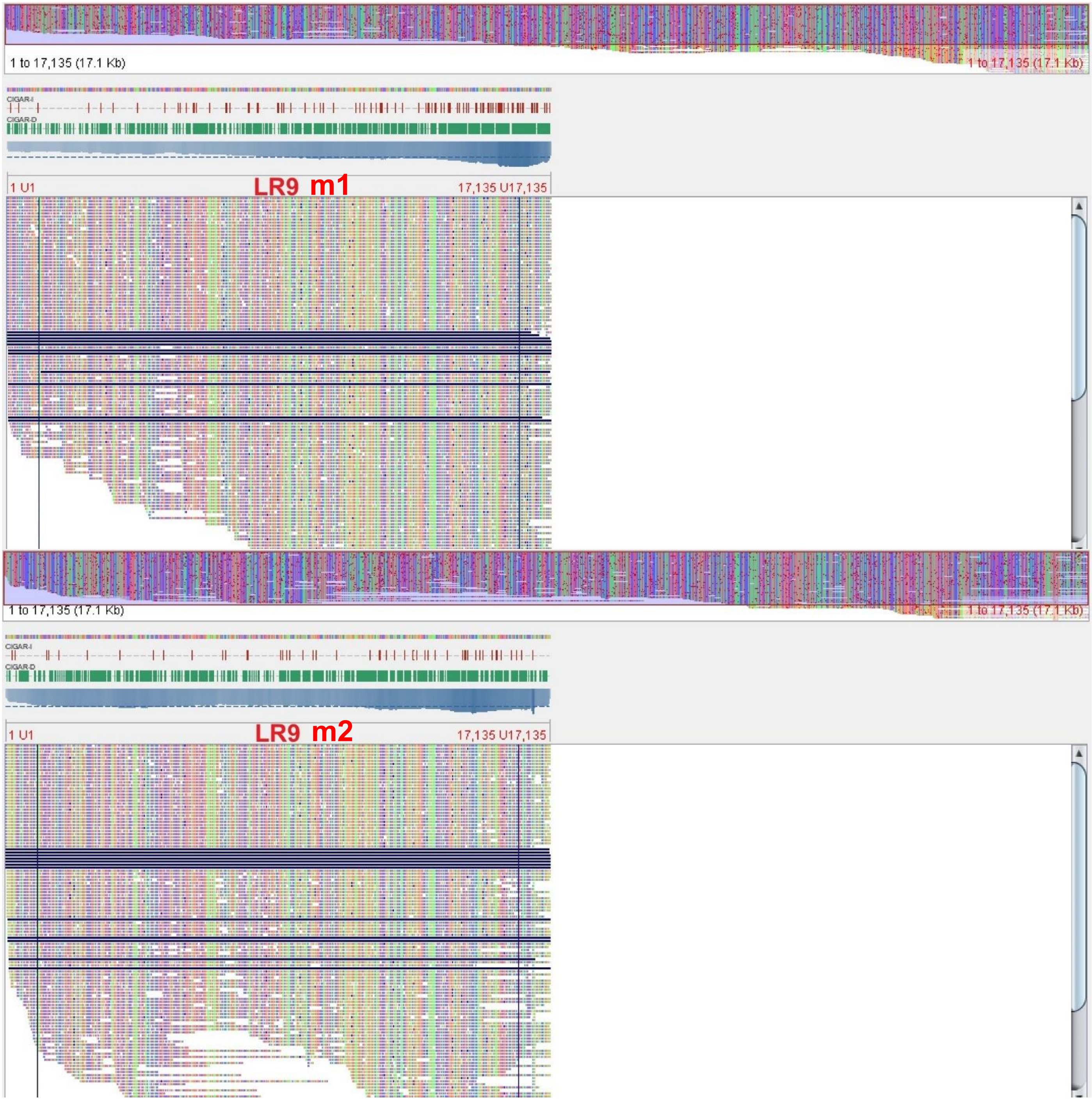

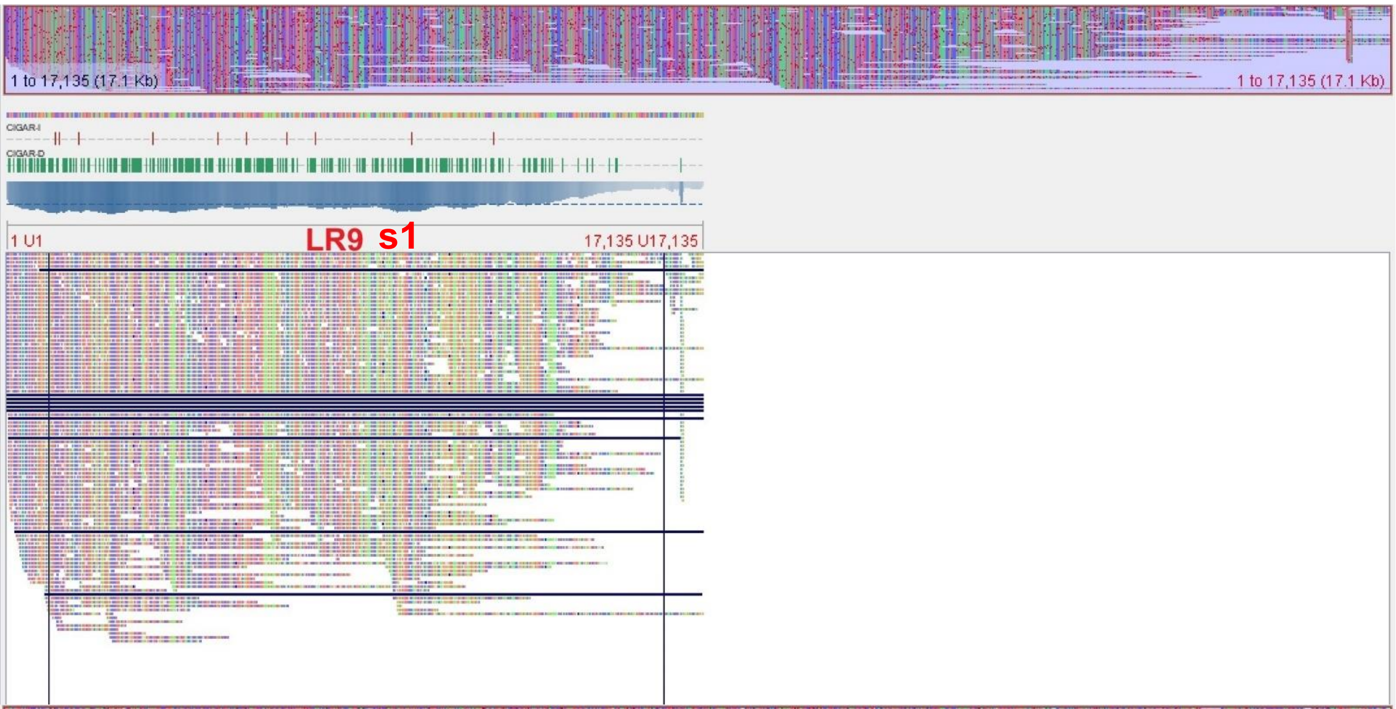
1 to 17,135 (17.1 Kb)
1 to 17,135 (17.1 Kb)
CIGAR-I
CIGAR-D
1 U1
LR9 s1
17,135 U17,135

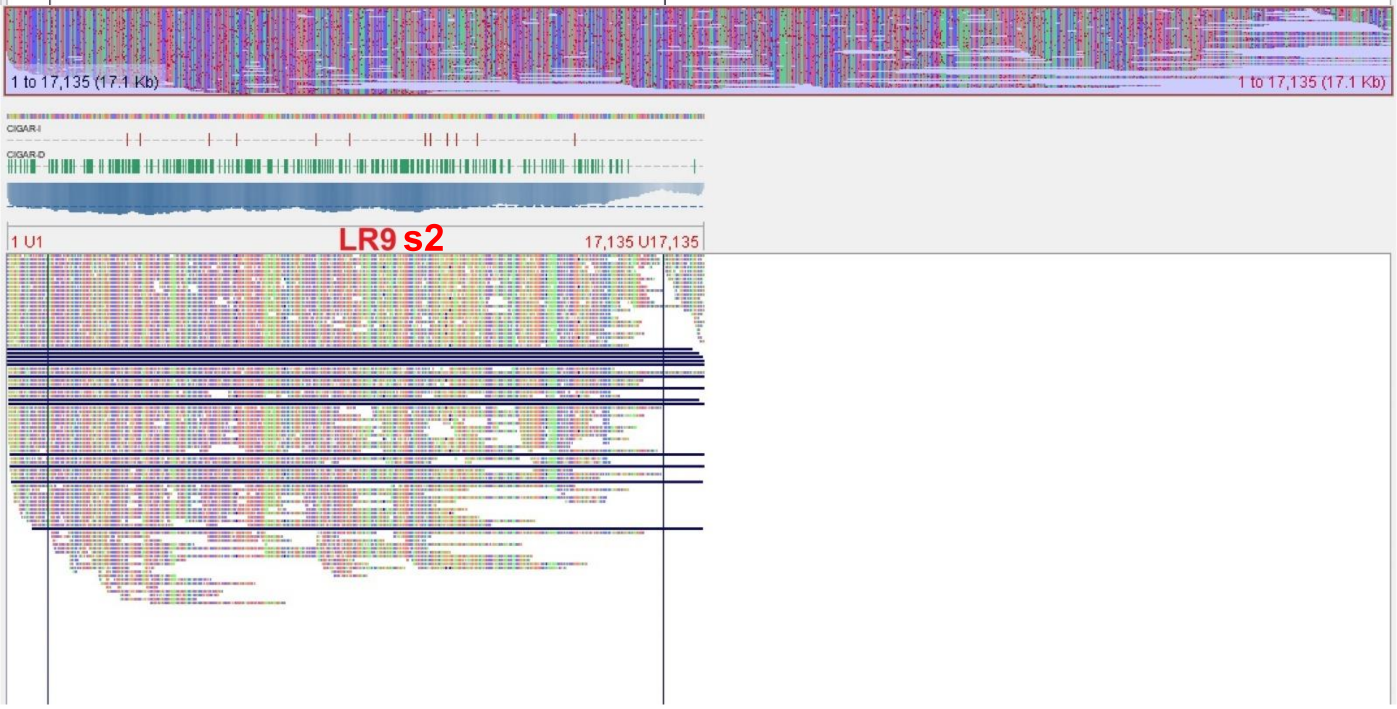
1 to 17,135 (17.1 Kb)
1 to 17,135 (17.1 Kb)
CIGAR-I
CIGAR-D
1 U1
LR9 s2
17,135 U17,135

B

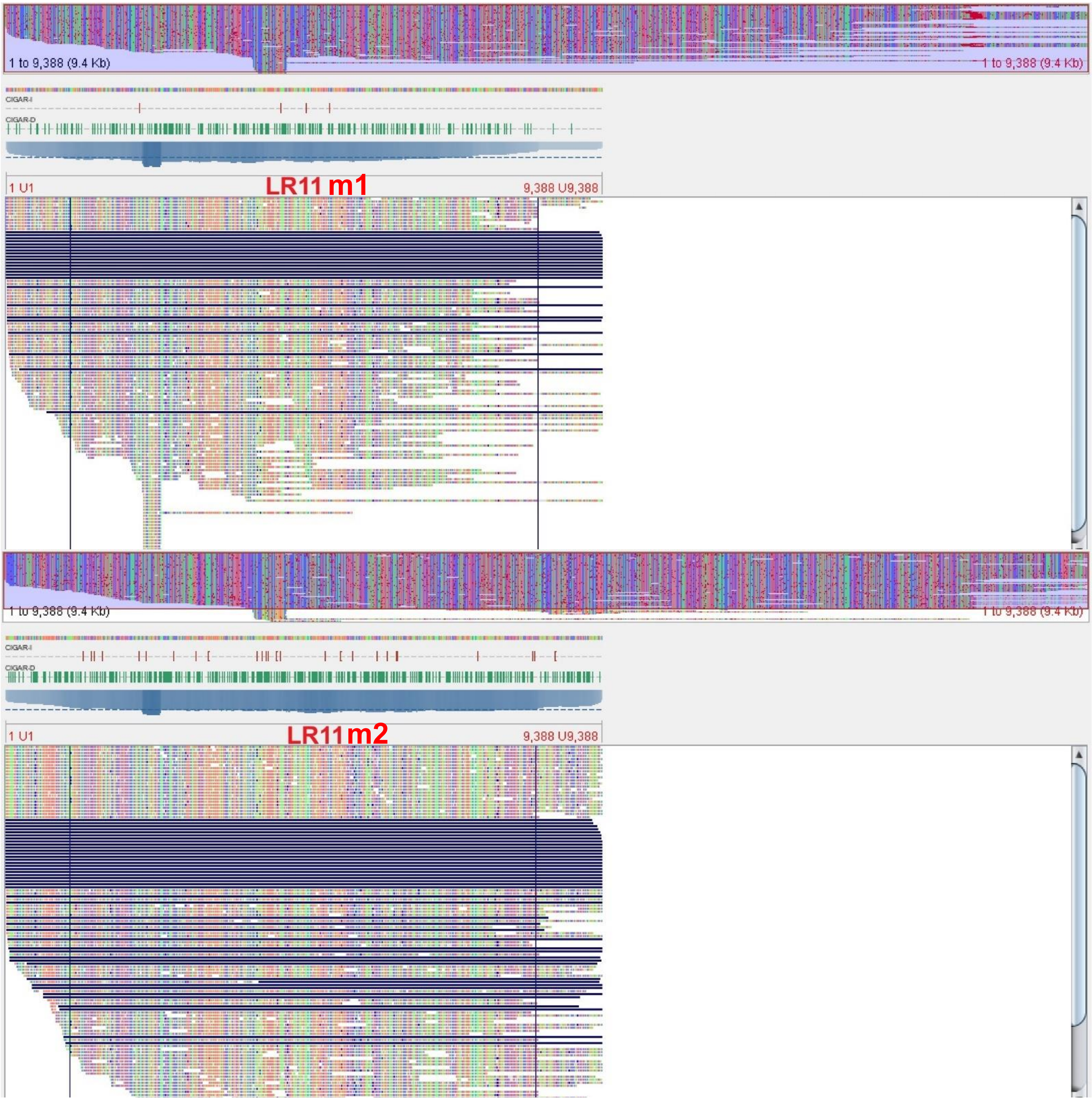
1 to 9,388 (9.4 Kb)
CIGAR-I
CIGAR-D
1 U1
LR11 m1
9,388 U9,388
1 to 9,388 (9.4 Kb)
CIGAR-I
CIGAR-D
1 U1
LR11m2
9,388 U9,388

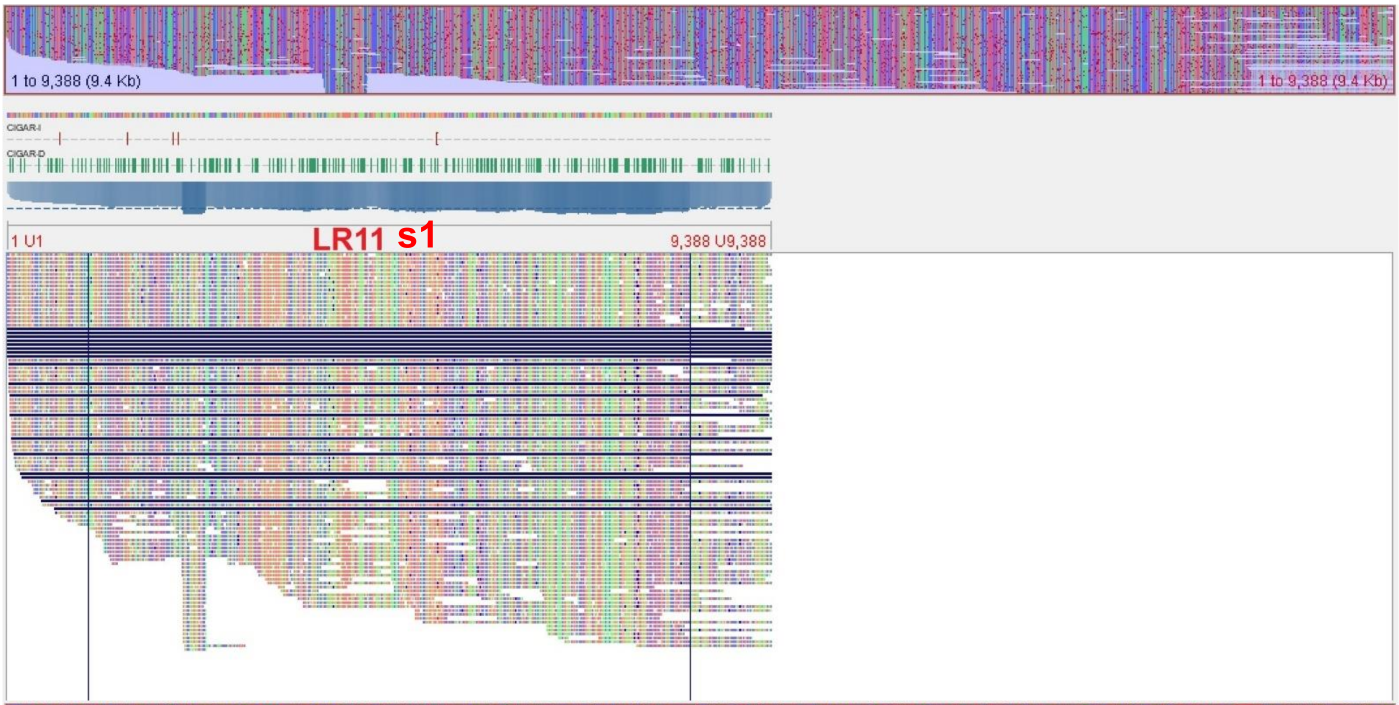
1 to 9,388 (9.4 Kb)
CIGAR-I
CIGAR-D
1 U1
LR11 s1
9,388 U9,388

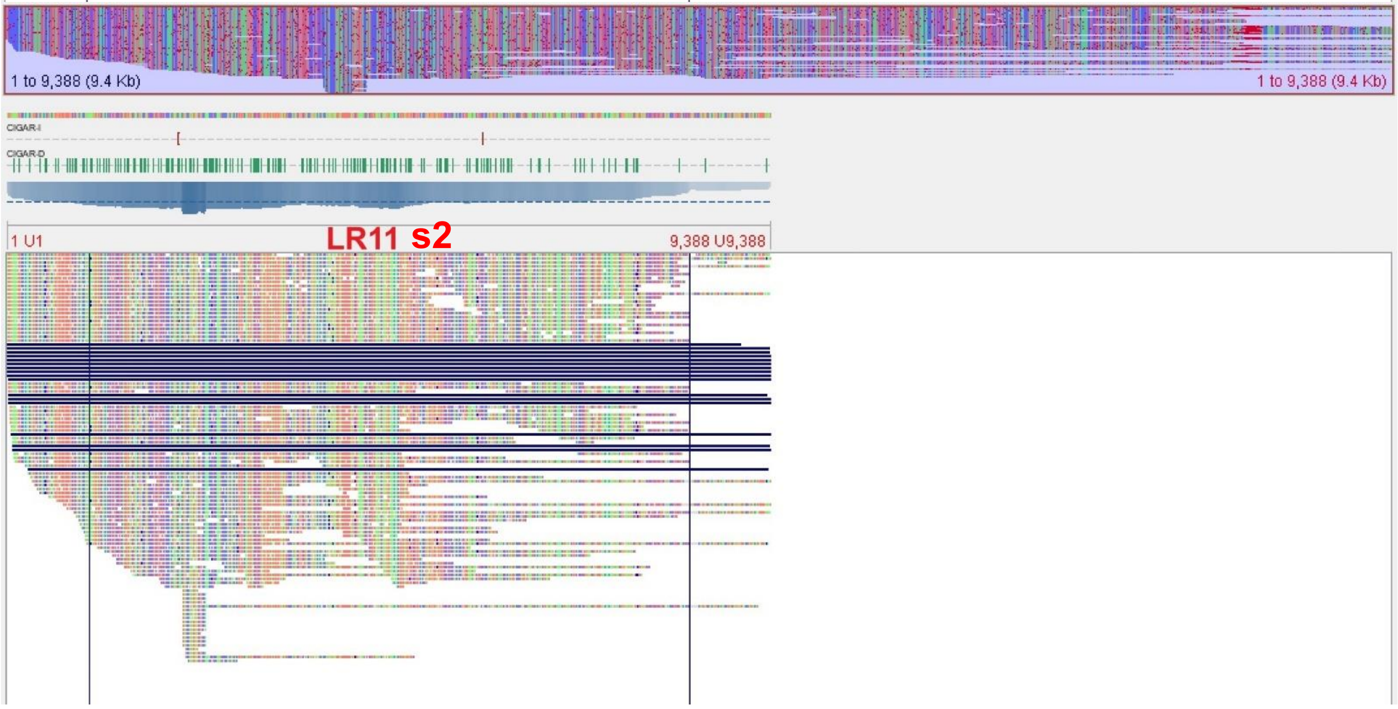
1 to 9,388 (9.4 Kb)
CIGAR-I
CIGAR-D
1 U1
LR11 s2
9,388 U9,388

C

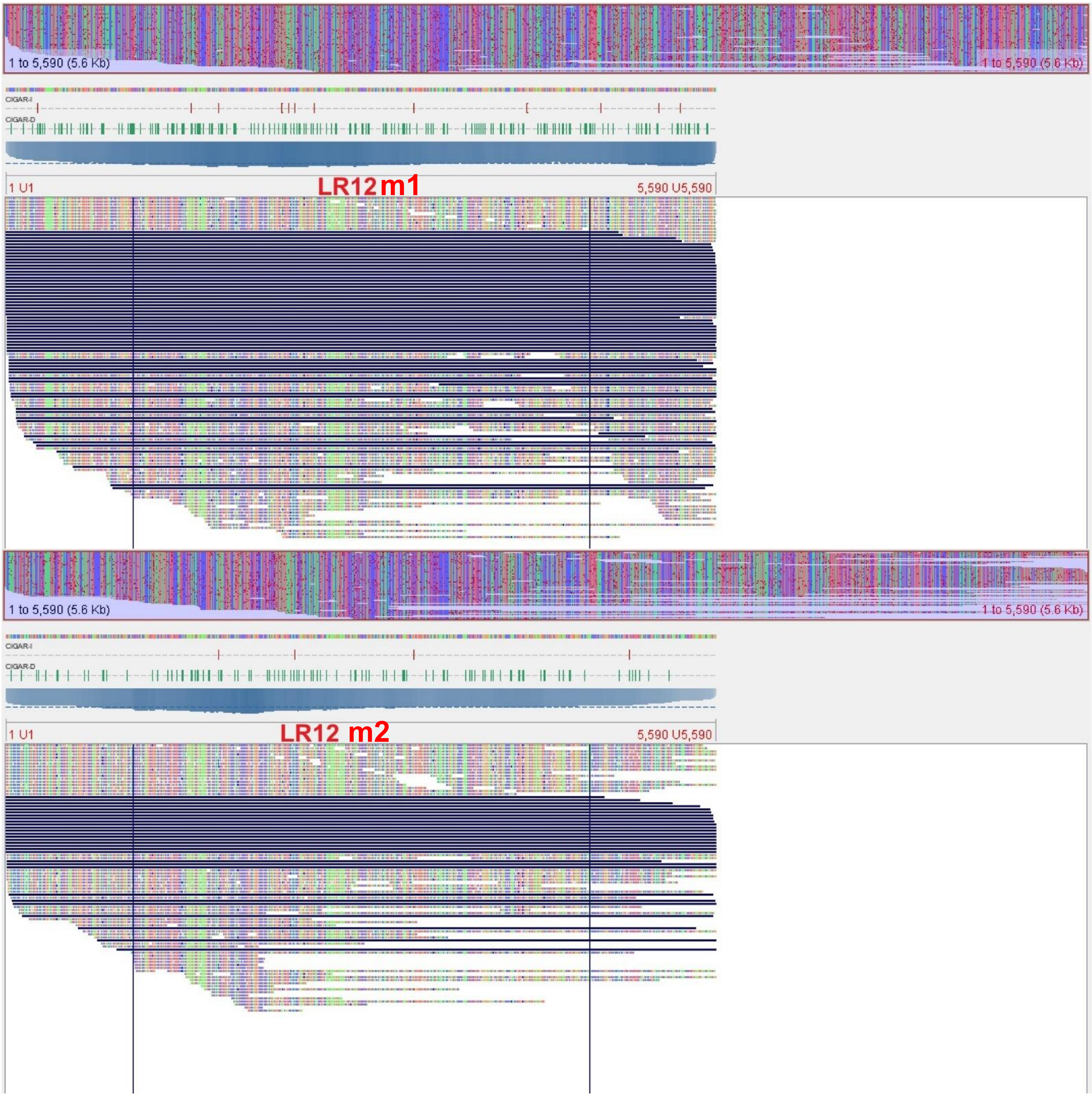
1 to 5,590 (5.6 Kb)
CIGAR-I
CIGAR-D
1 U1
LR12 m1
5,590 U5,590
1 to 5,590 (5.6 Kb)
CIGAR-I
CIGAR-D
1 U1
LR12 m2
5,590 U5,590

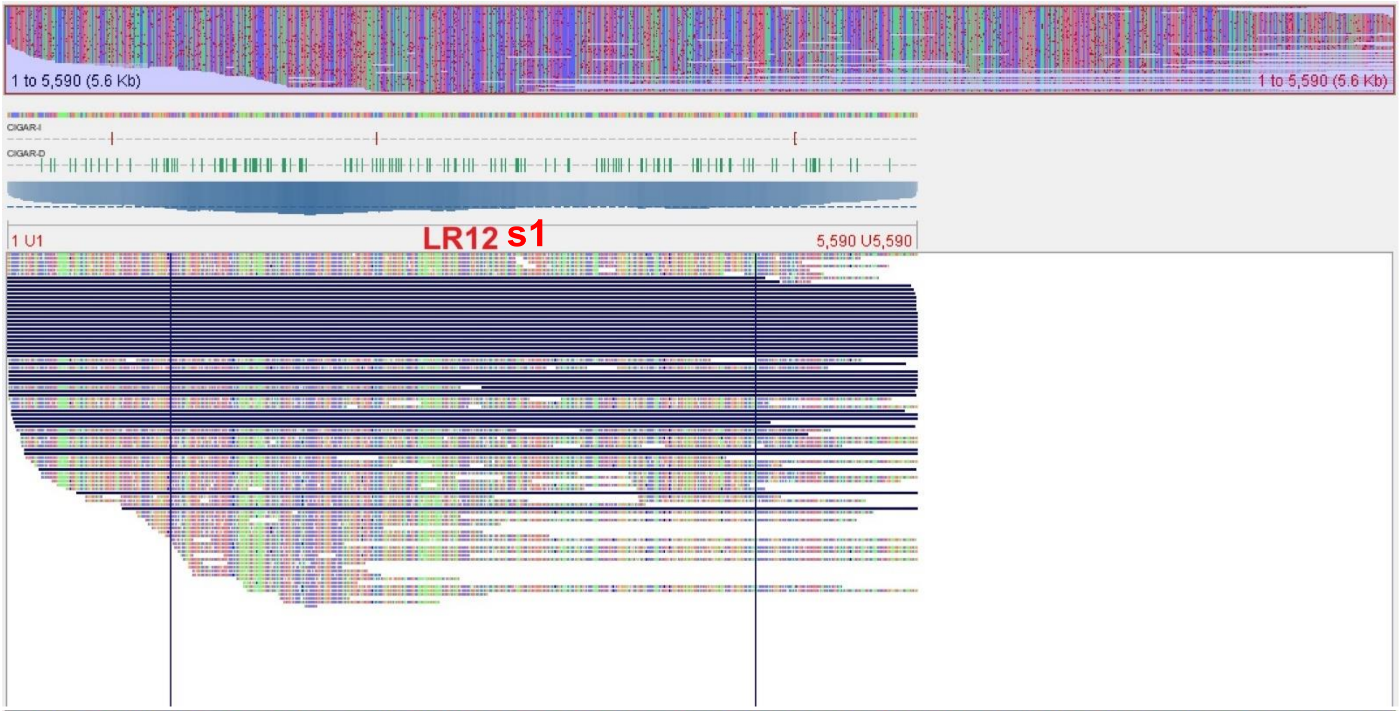
1 to 5,590 (5.6 Kb)
1 to 5,590 (5.6 Kb)
CIGAR-I
CIGAR-D
1 U1
LR12 s1
5,590 U5,590

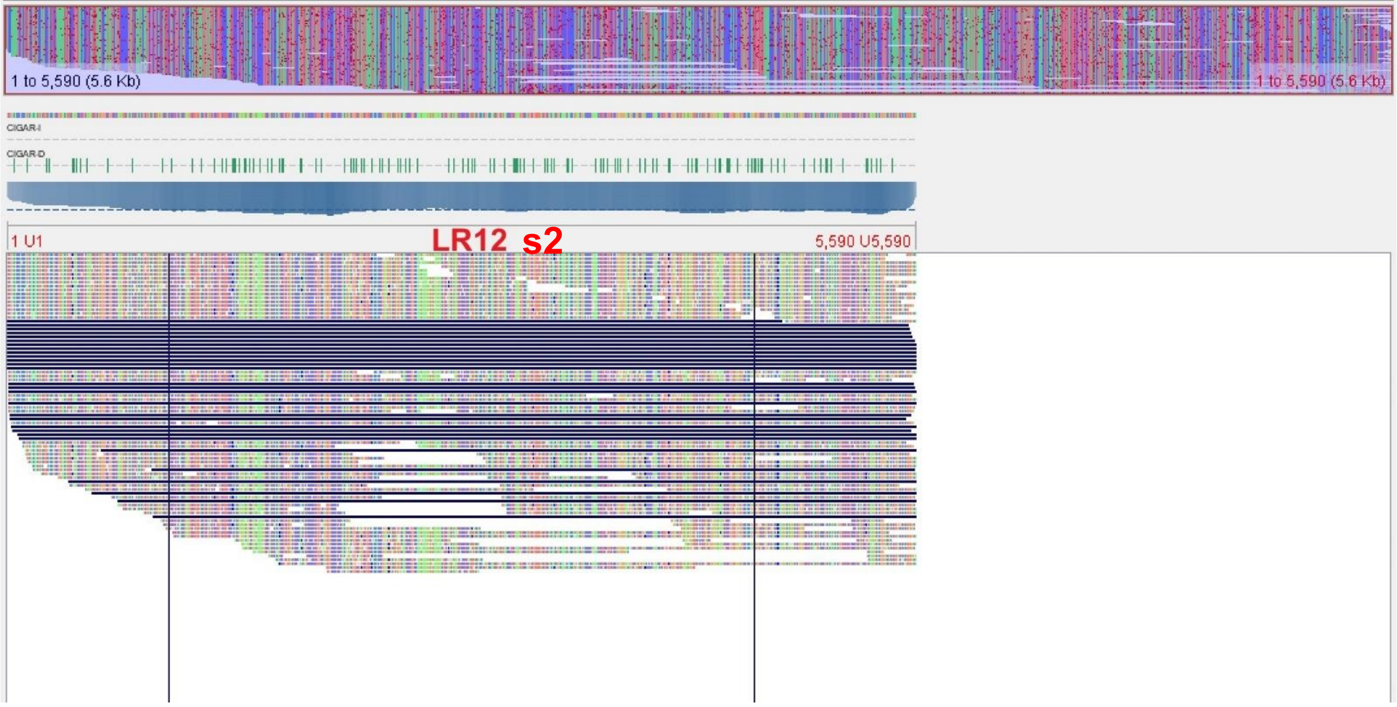
1 to 5,590 (5.6 Kb)
1 to 5,590 (5.6 Kb)
CIGAR-I
CIGAR-D
1 U1
LR12 s2
5,590 U5,590

D

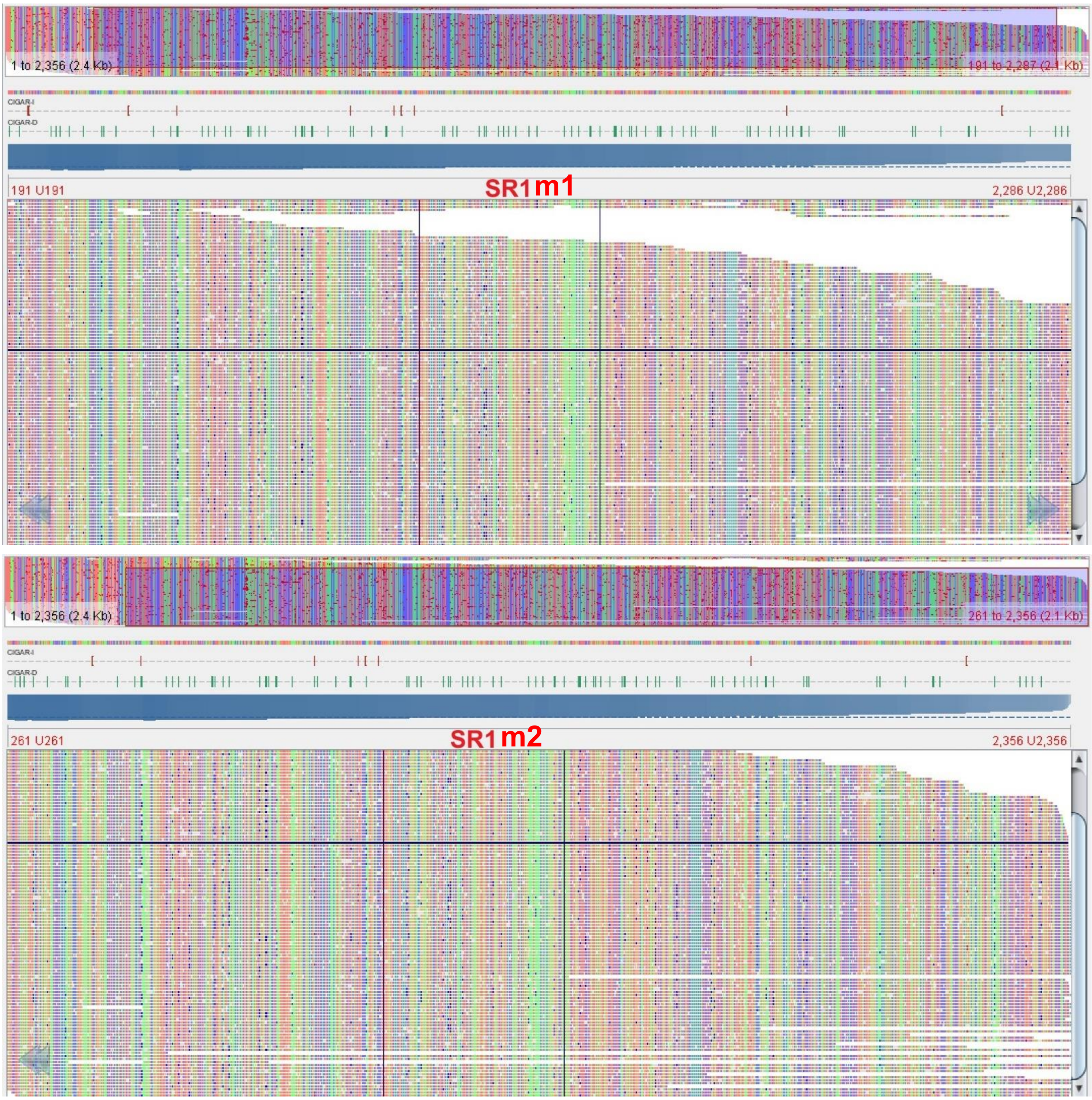
1 to 2,356 (2.4 Kb)
191 to 2,287 (2.1 Kb)
CIGAR-I
CIGAR-D
191 U191
SR1m1
2,286 U2,286
1 to 2,356 (2.4 Kb)
261 to 2,356 (2.1 Kb)
CIGAR-I
CIGAR-D
261 U261
SR1m2
2,356 U2,356

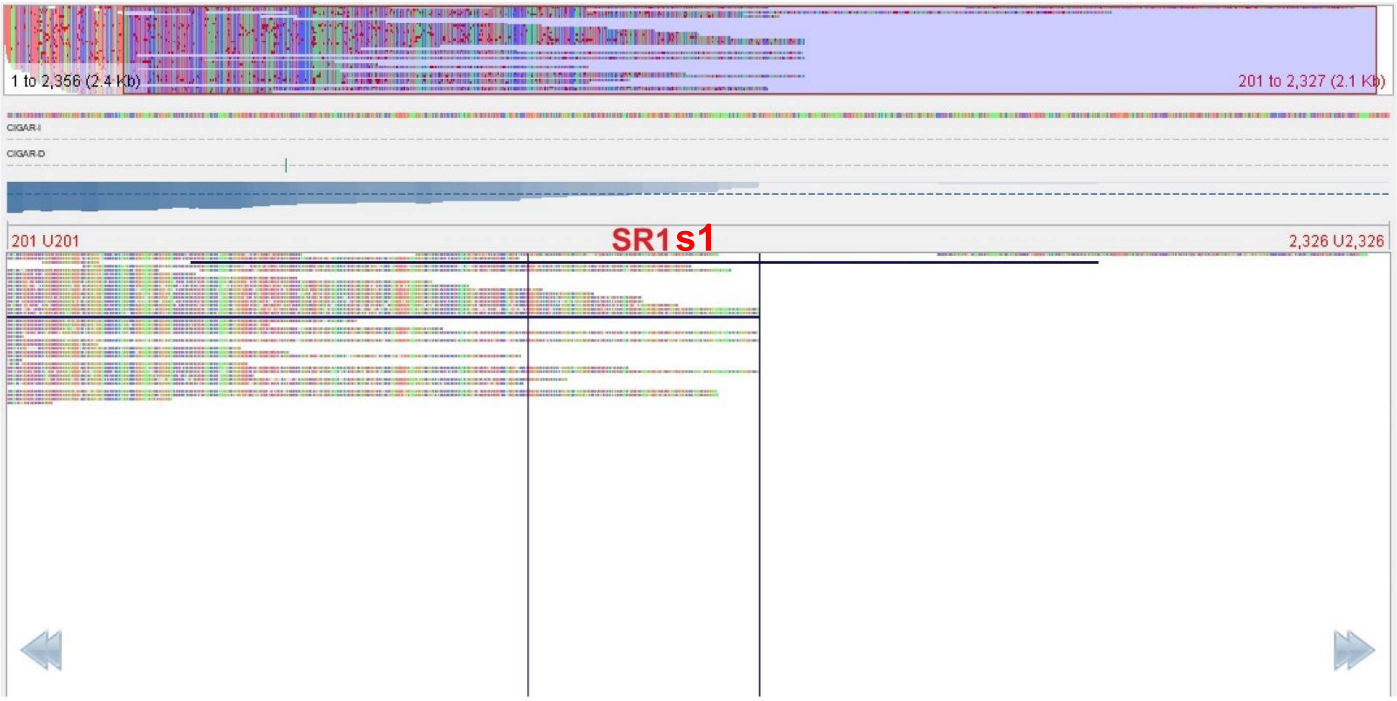
1 to 2,356 (2.4 Kb)
201 to 2,327 (2.1 Kb)
CIGAR-I
CIGAR-D
201 U201
SR1s1
2,326 U2,326

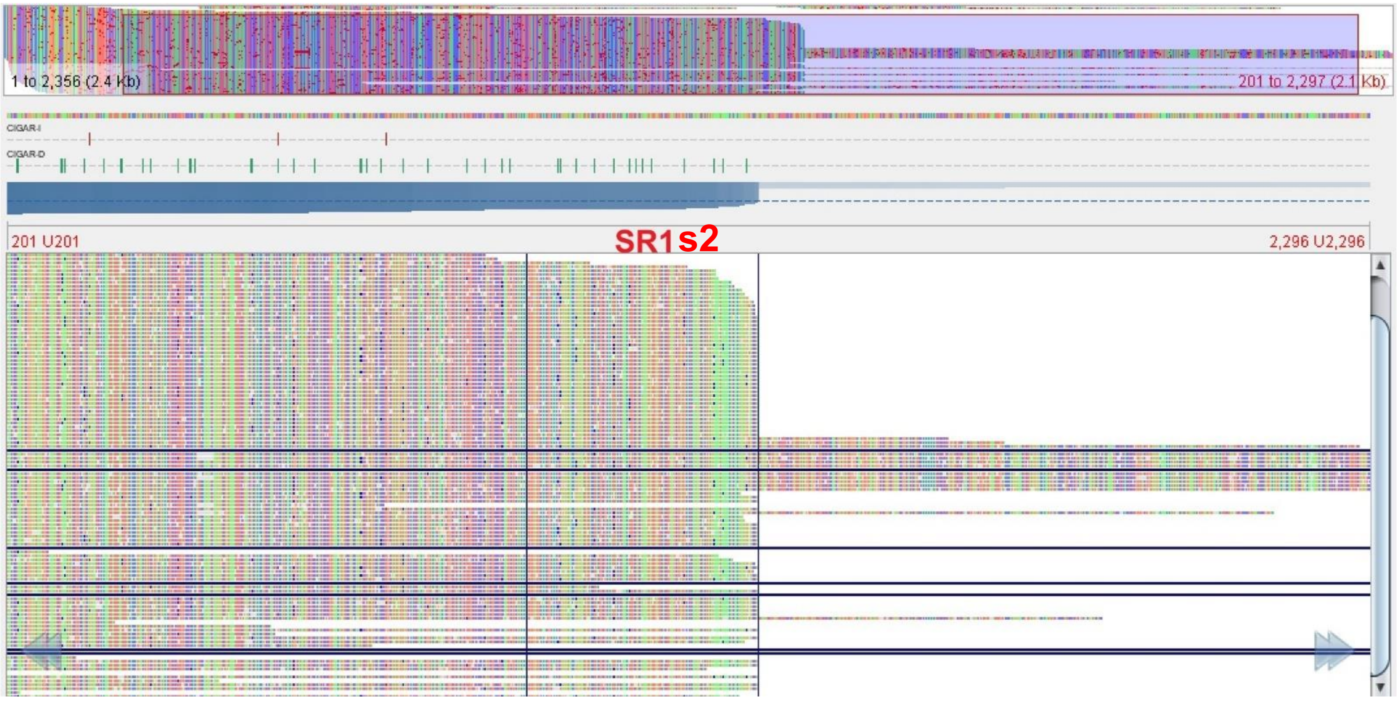
1 to 2,356 (2.4 Kb)
201 to 2,297 (2.1 Kb)
CIGAR-I
CIGAR-D
201 U201
SR1s2
2,296 U2,296

E

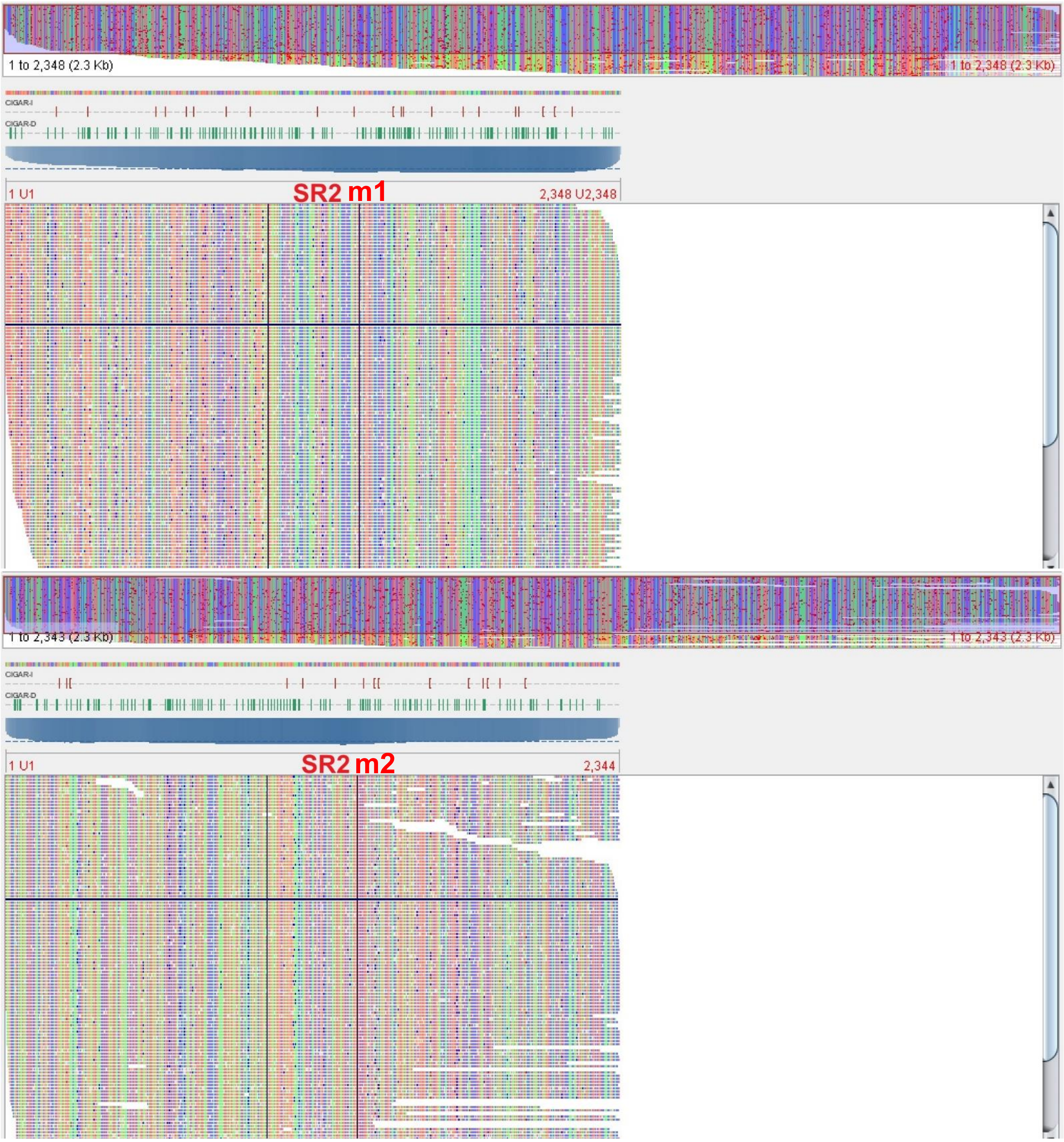
1 to 2,348 (2.3 Kb)
CIGAR-I
CIGAR-D
1 U1
SR2 m1
2,348 U2,348
1 to 2,343 (2.3 Kb)
CIGAR-I
CIGAR-D
1 U1
SR2 m2
2,344

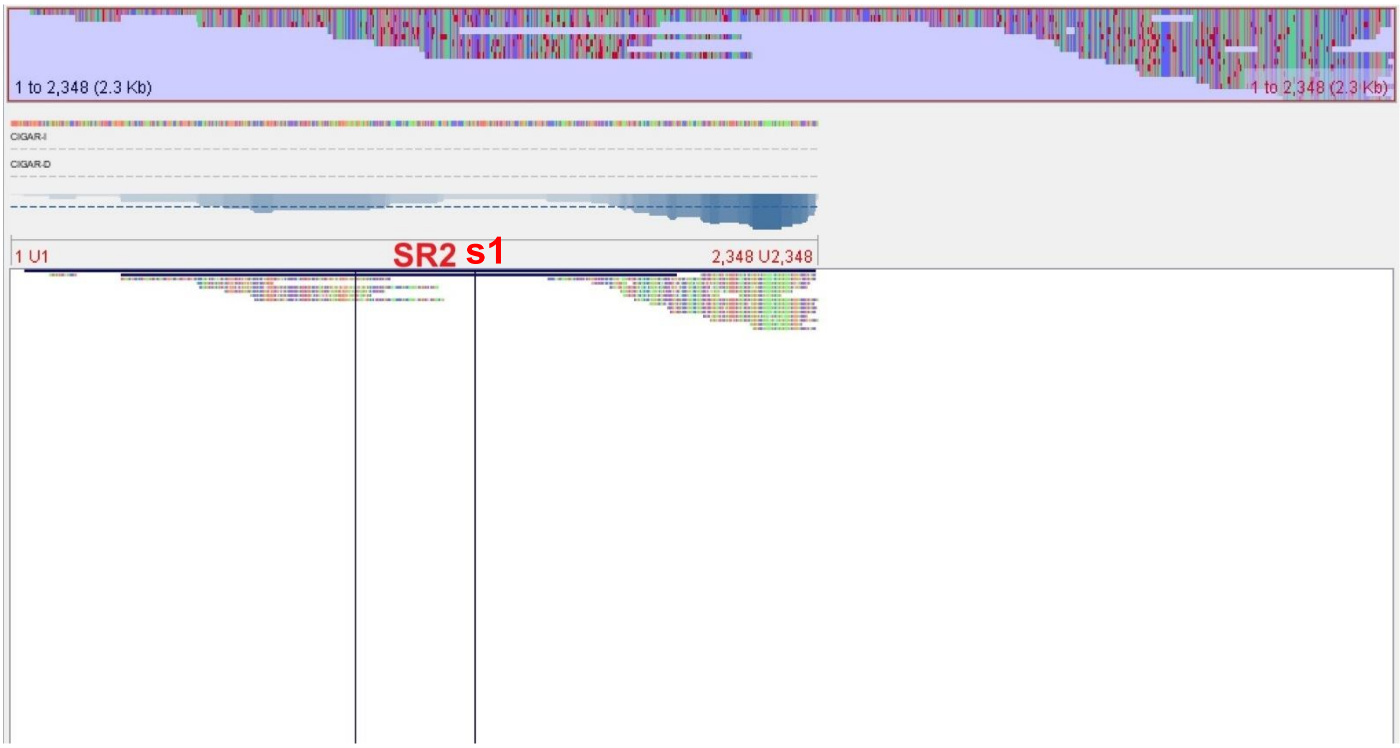
1 to 2,348 (2.3 Kb)
CIGAR-I
CIGAR-D
1 U1
SR2 s1
2,348 U2,348

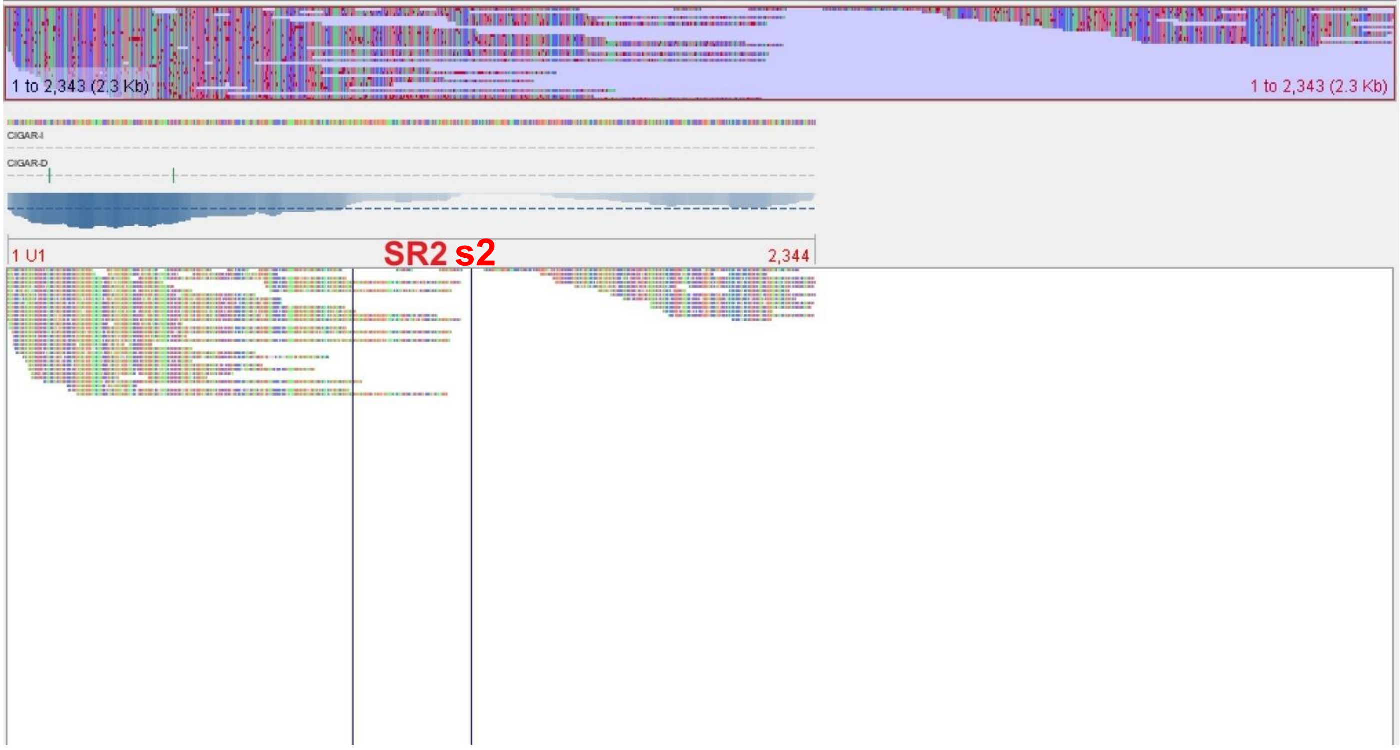
1 to 2,343 (2.3 Kb)
CIGAR-I
CIGAR-D
1 U1
SR2 s2
2,344

F

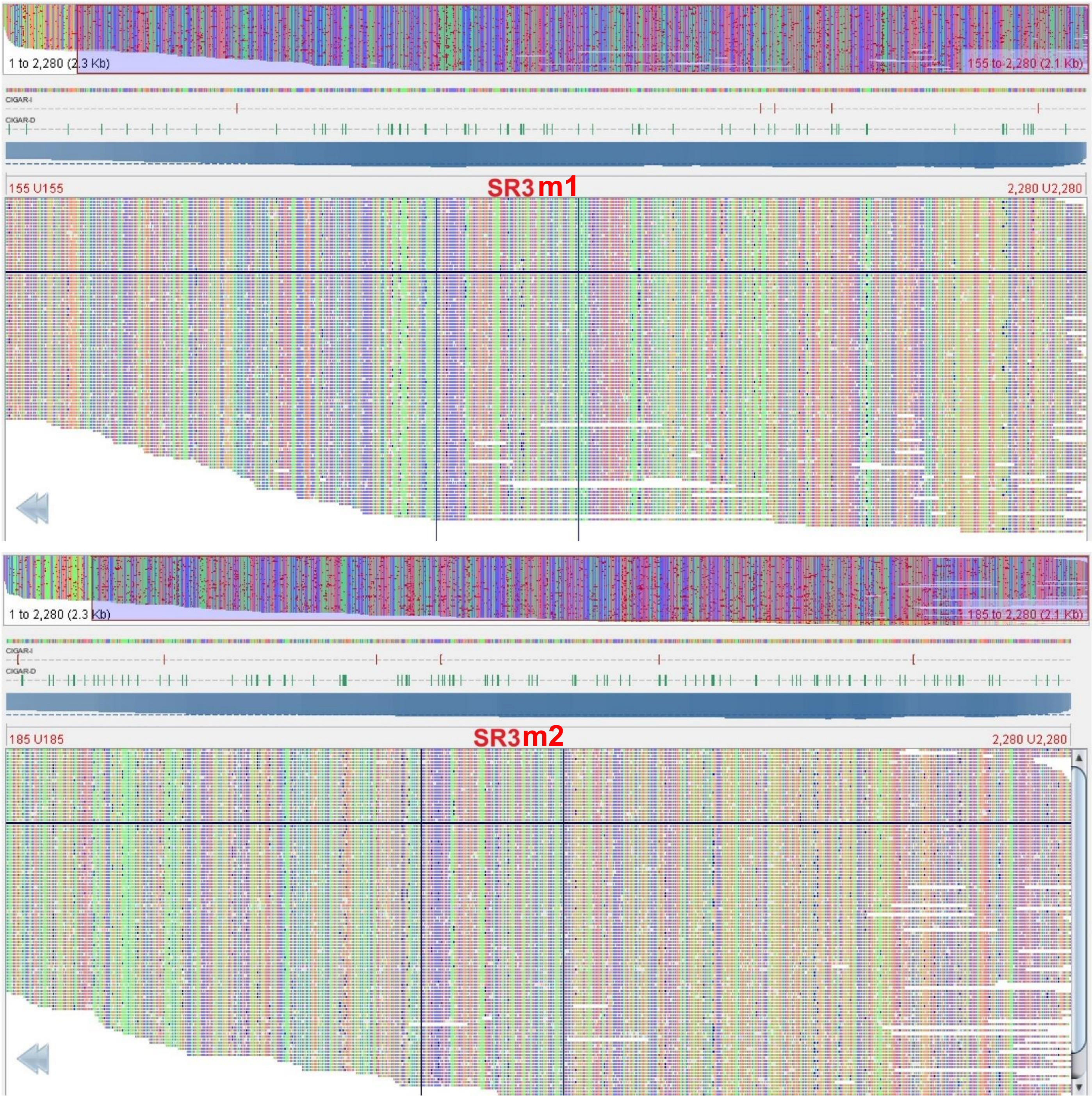

1 to 2,280 (2.3 Kb)
155 to 2,280 (2.1 Kb)
CIGAR-I
CIGAR-D
155 U155
SR3m1
2,280 U2,280
1 to 2,280 (2.3 Kb)
185 to 2,280 (2.1 Kb)
CIGAR-I
CIGAR-D
185 U185
SR3m2
2,280 U2,280

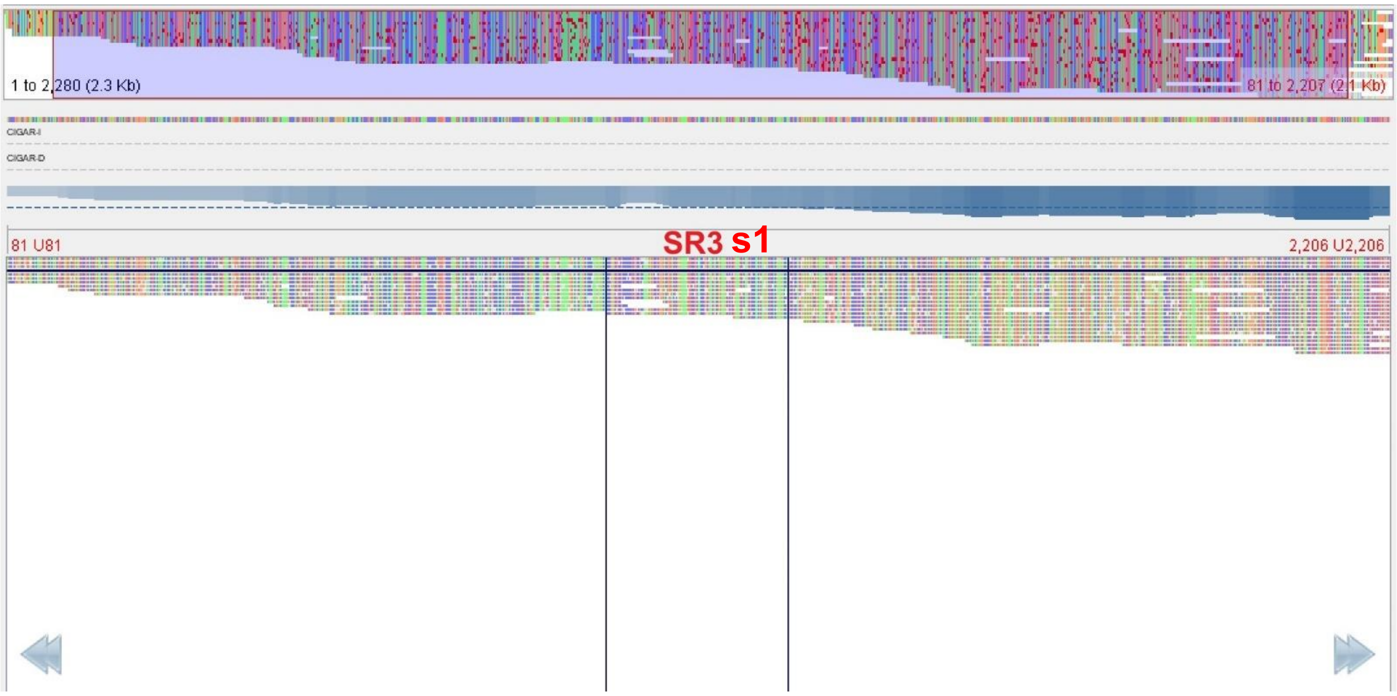
1 to 2,280 (2.3 Kb)
81 to 2,207 (2.1 Kb)
CIGAR-I
CIGAR-D
81 U81
SR3 s1
2,206 U2,206

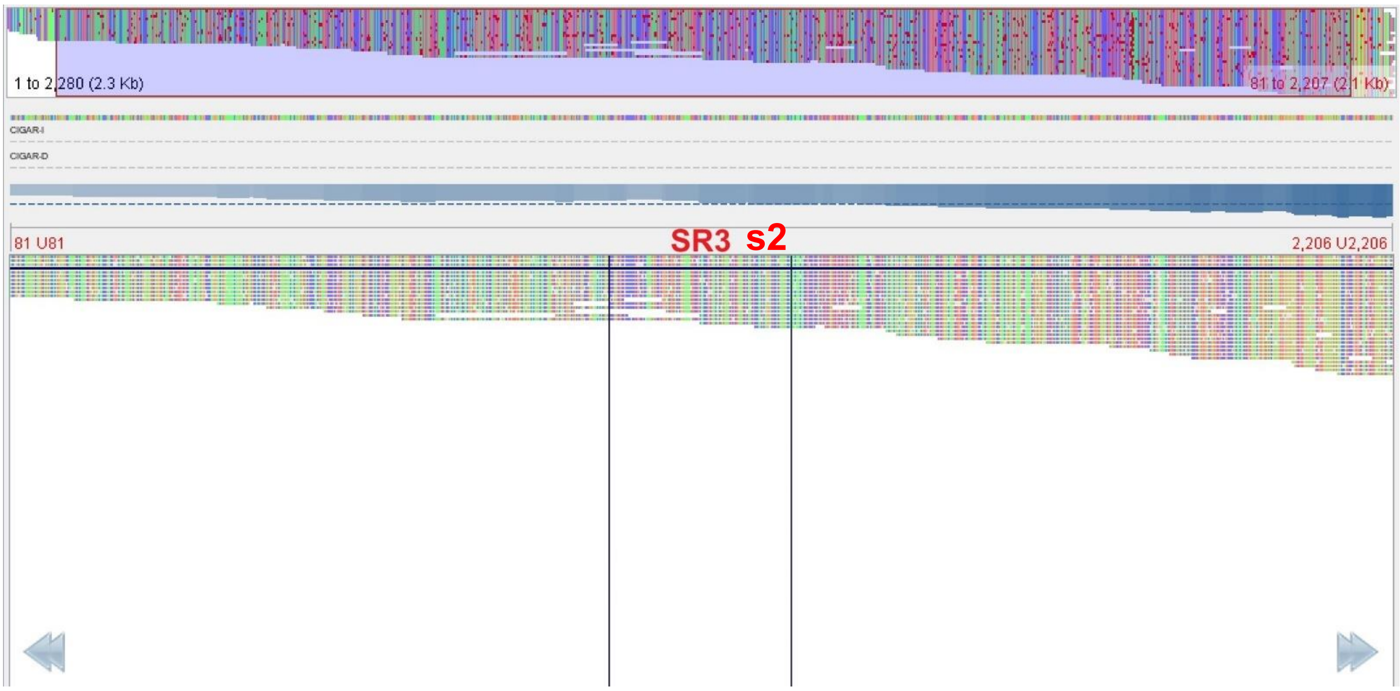
1 to 2,280 (2.3 Kb)
81 to 2,207 (2.1 Kb)
CIGAR-I
CIGAR-D
81 U81
SR3 s2
2,206 U2,206

G

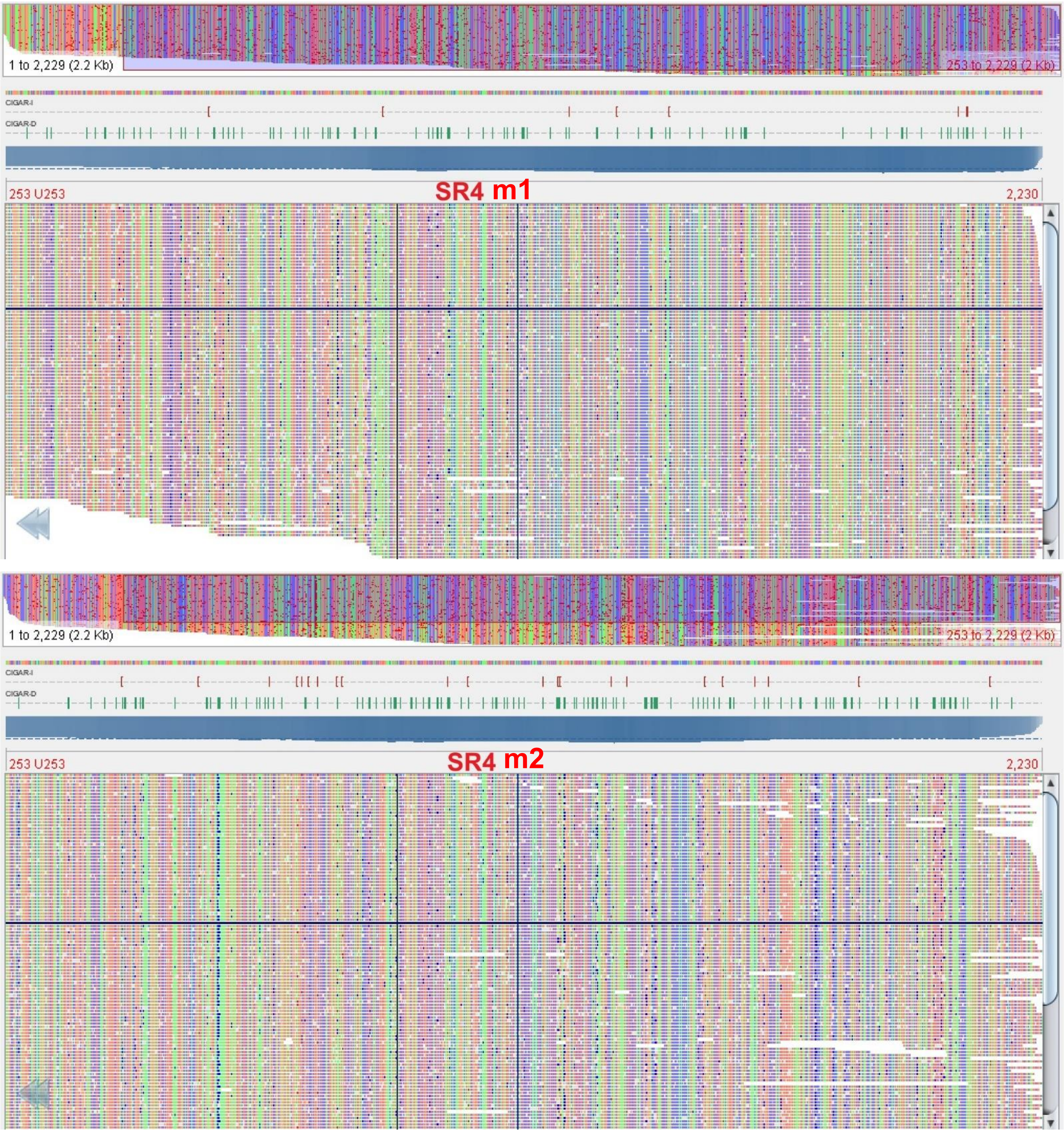
1 to 2,229 (2.2 Kb)
253 to 2,229 (2 Kb)
CIGAR-I
CIGAR-D
253 U253
SR4 m1
2,230
1 to 2,229 (2.2 Kb)
253 to 2,229 (2 Kb)
CIGAR-I
CIGAR-D
253 U253
SR4 m2
2,230

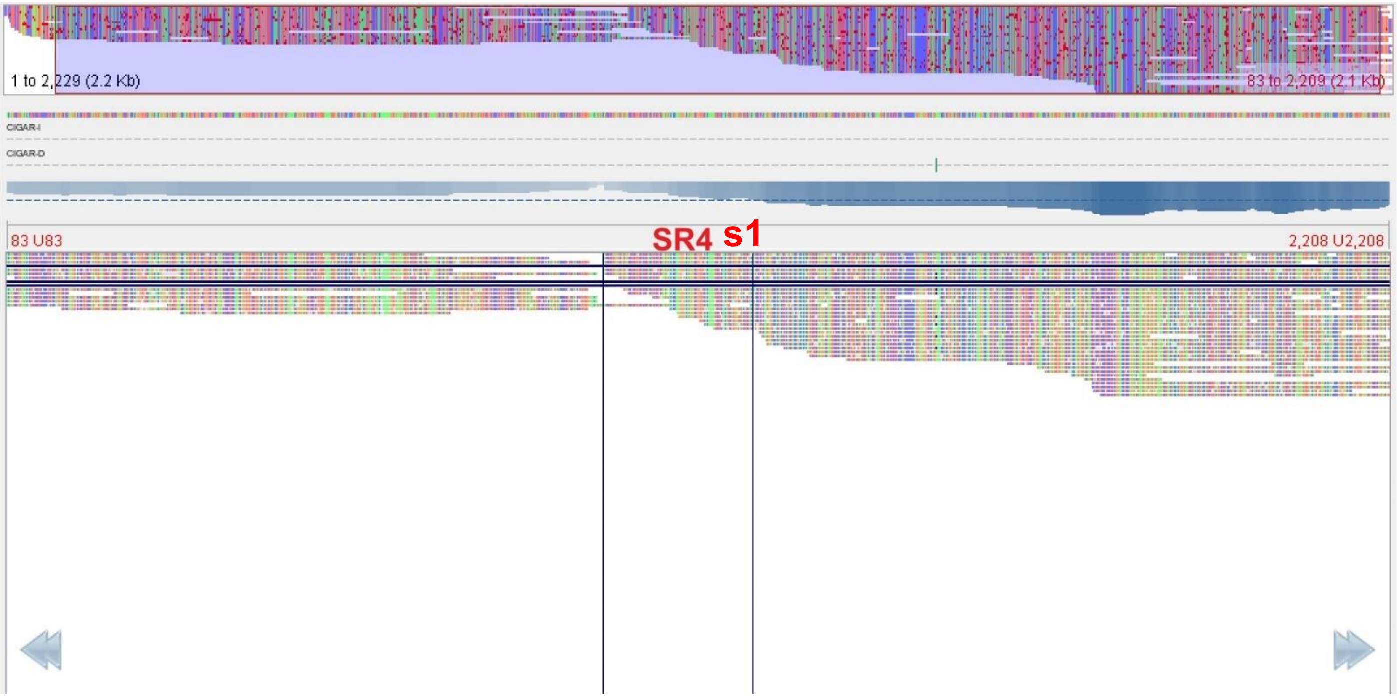
1 to 2,229 (2.2 Kb)
83 to 2,209 (2.1 Kb)
CIGAR-I
CIGAR-D
83 U83
SR4 s1
2,208 U2,208

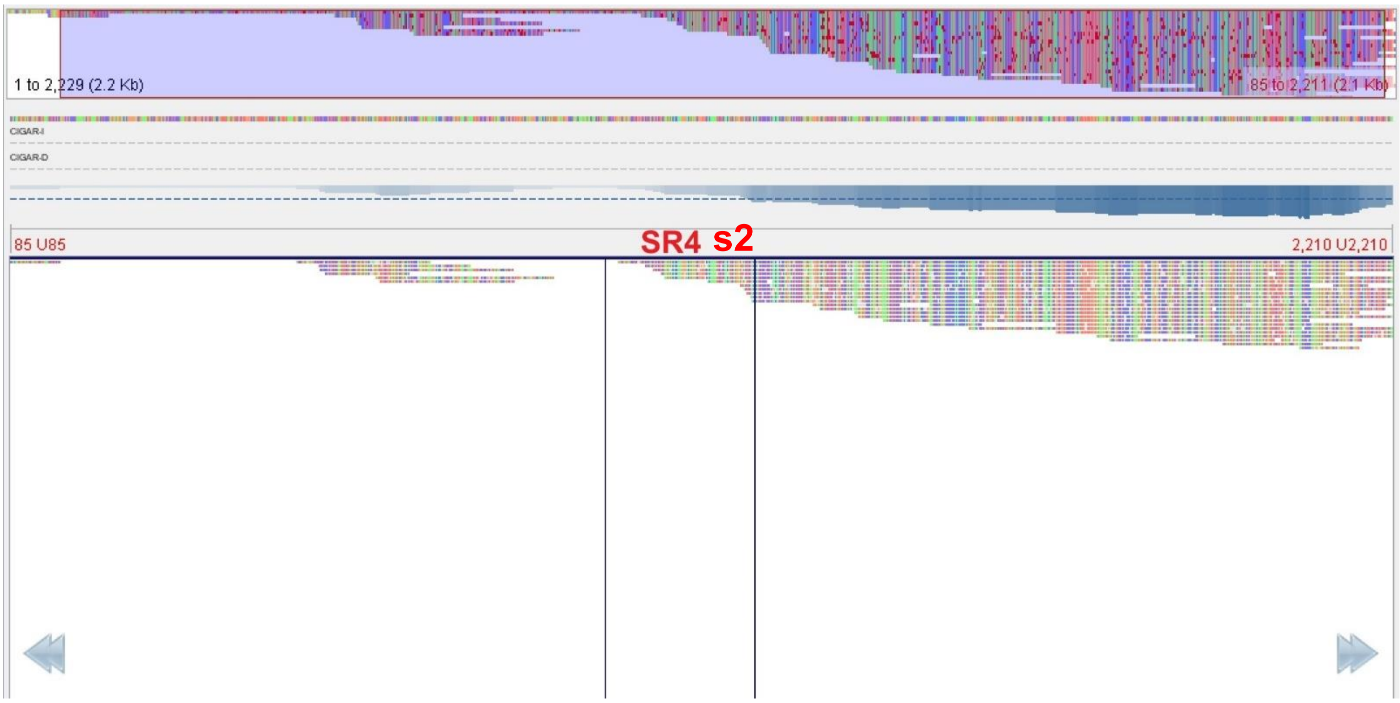
1 to 2,229 (2.2 Kb)
85 to 2,211 (2.1 Kb)
CIGAR-I
CIGAR-D
85 U85
SR4 s2
2,210 U2,210

H

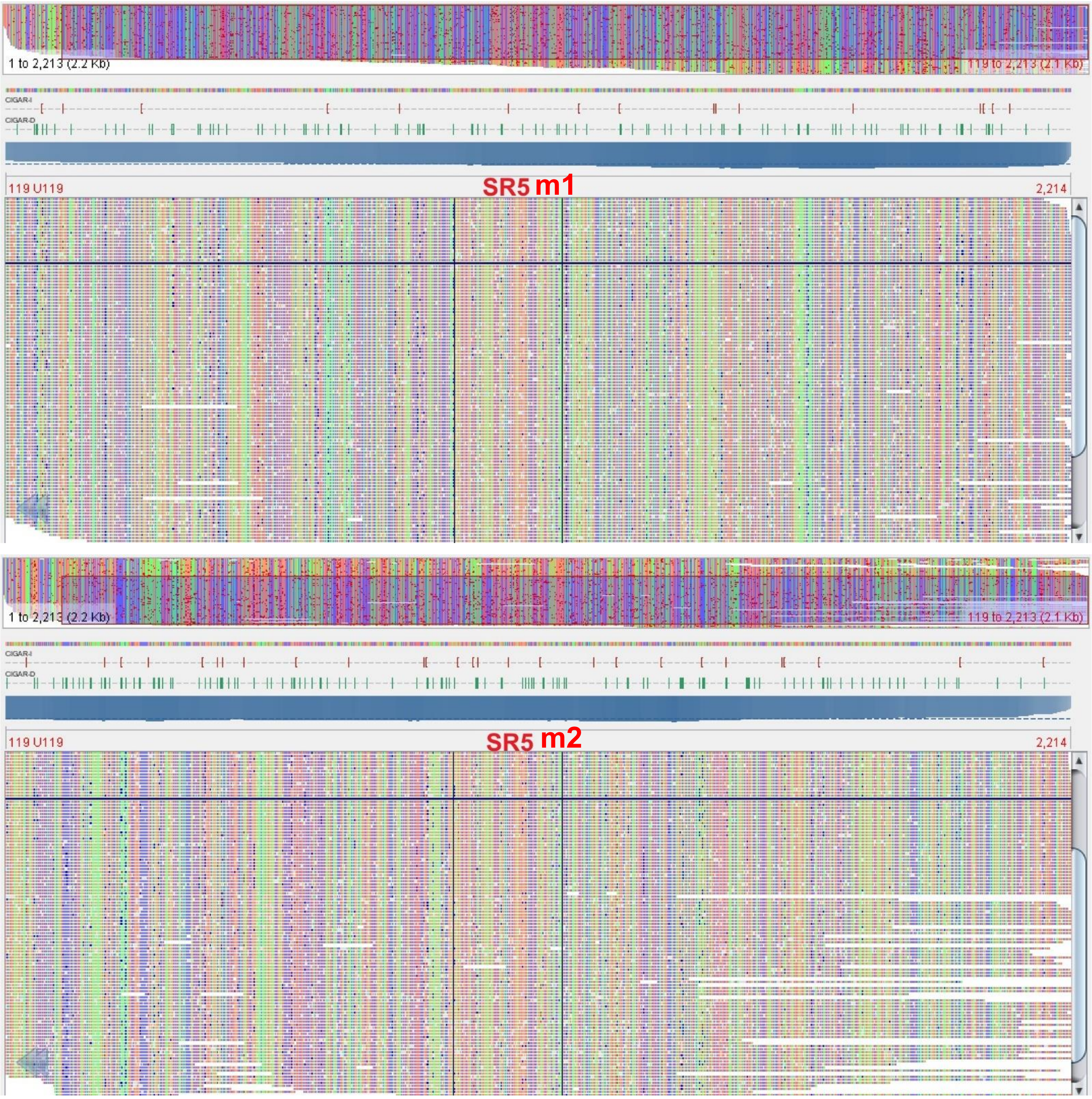

1 to 2,213 (2.2 Kb)
119 to 2,213 (2.1 Kb)
CIGAR-I
CIGAR-D
119 U119
SR5 m1
2,214
1 to 2,213 (2.2 Kb)
119 to 2,213 (2.1 Kb)
CIGAR-I
CIGAR-D
119 U119
SR5 m2
2,214

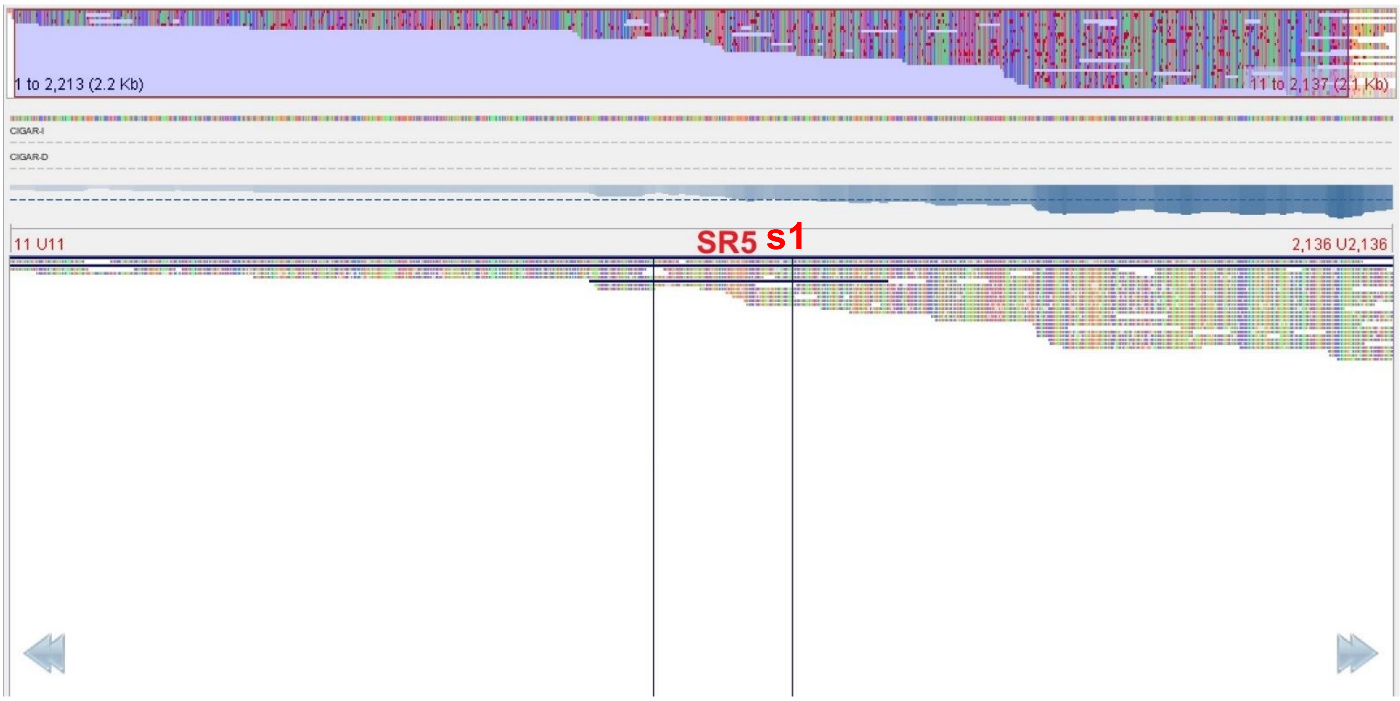
1 to 2,213 (2.2 Kb)
11 to 2,137 (2.1 Kb)
CIGAR-I
CIGAR-D
11 U11
SR5 s1
2,136 U2,136

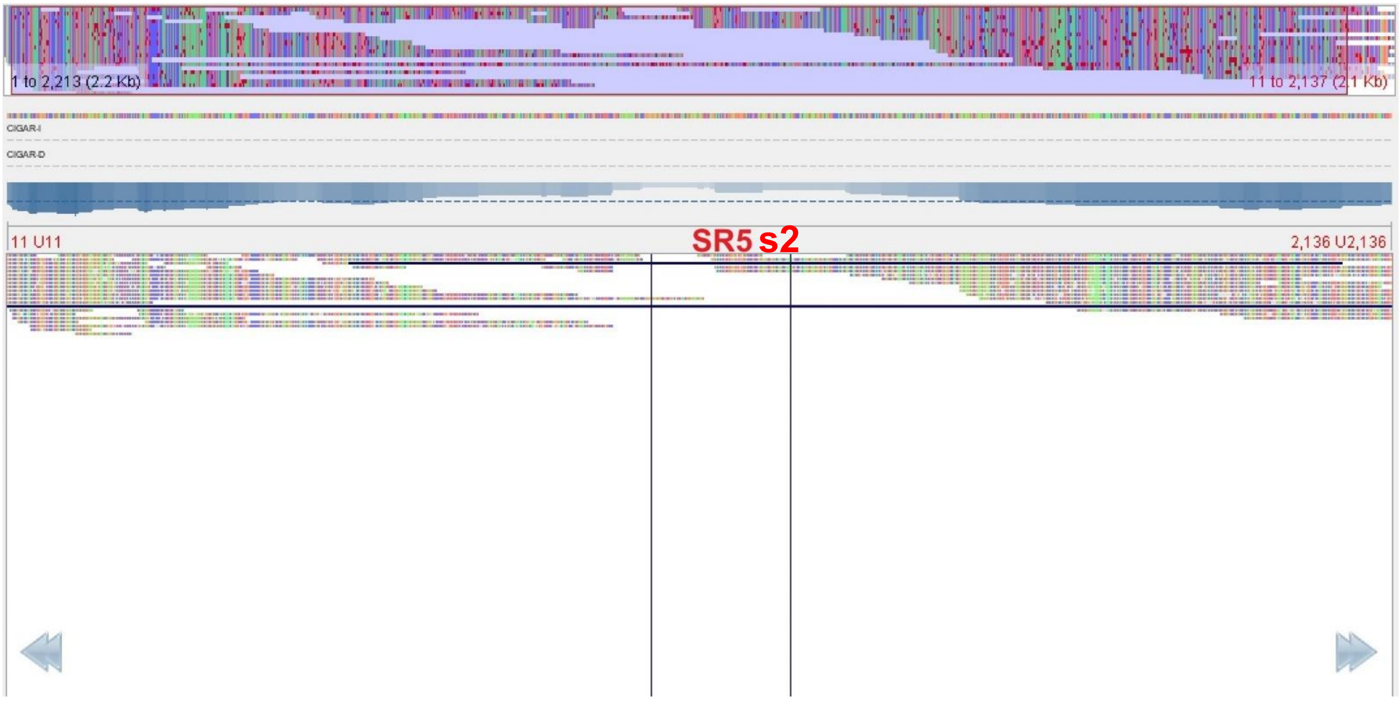
1 to 2,213 (2.2 Kb)
11 to 2,137 (2.1 Kb)
CIGAR-I
CIGAR-D
11 U11
SR5 s2
2,136 U2,136

I

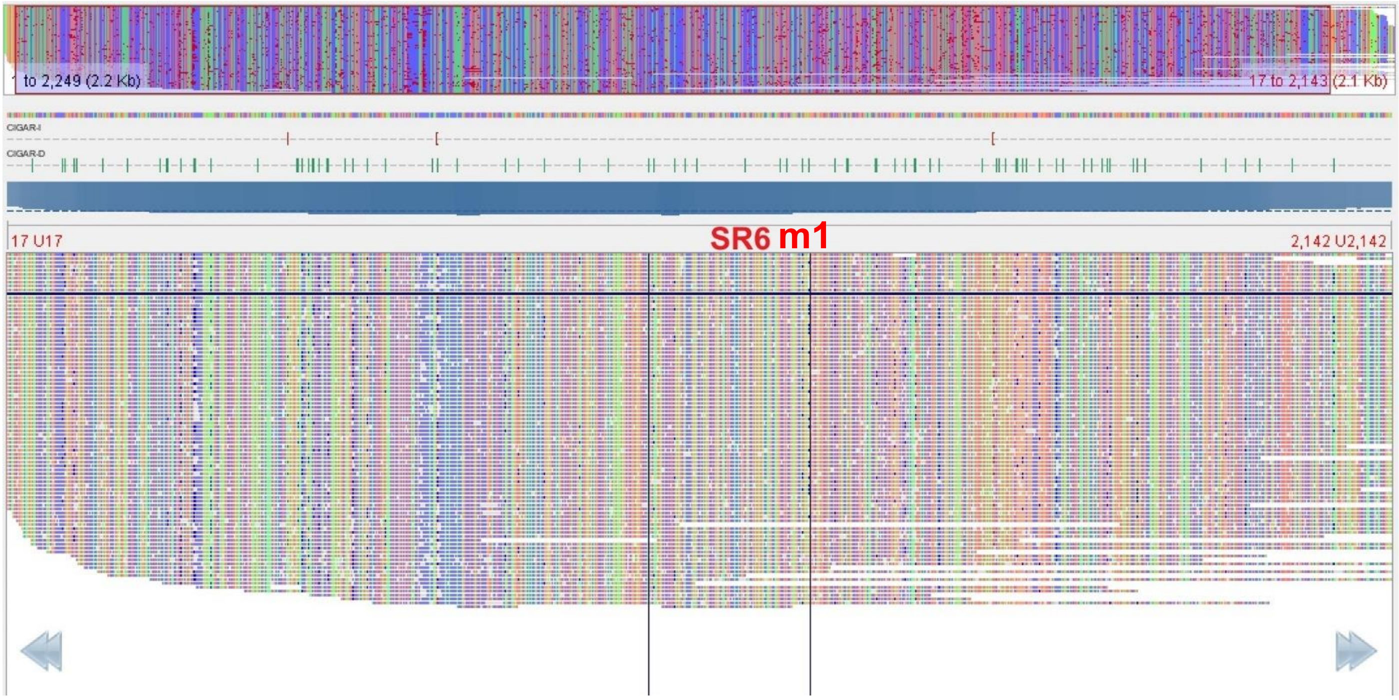
1 to 2,249 (2.2 Kb)
17 to 2,143 (2.1 Kb)
CIGAR-I
CIGAR-D
17 U17
SR6 m1
2,142 U2,142

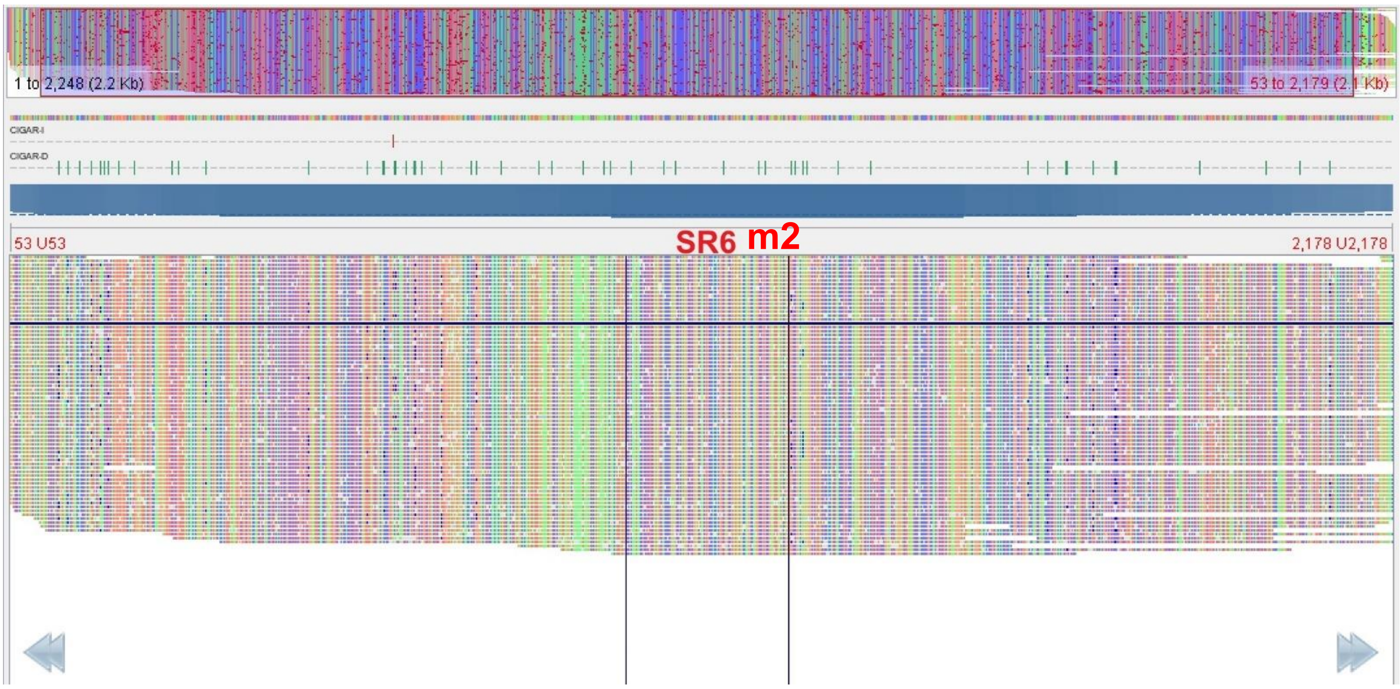
1 to 2,248 (2.2 Kb)
53 to 2,179 (2.1 Kb)
CIGAR-I
CIGAR-D
53 U53
SR6 m2
2,178 U2,178

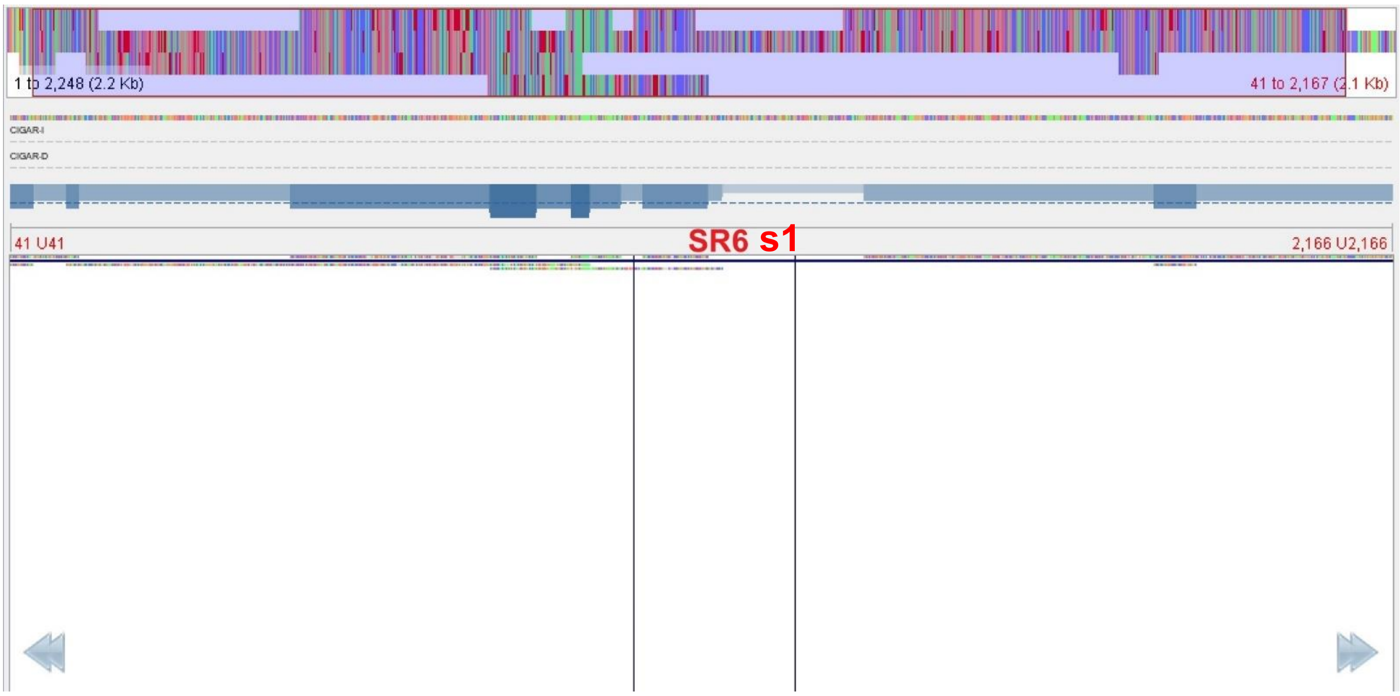
1 to 2,248 (2.2 Kb)
41 to 2,167 (2.1 Kb)
CIGAR-I
CIGAR-D
41 U41
SR6 s1
2,166 U2,166

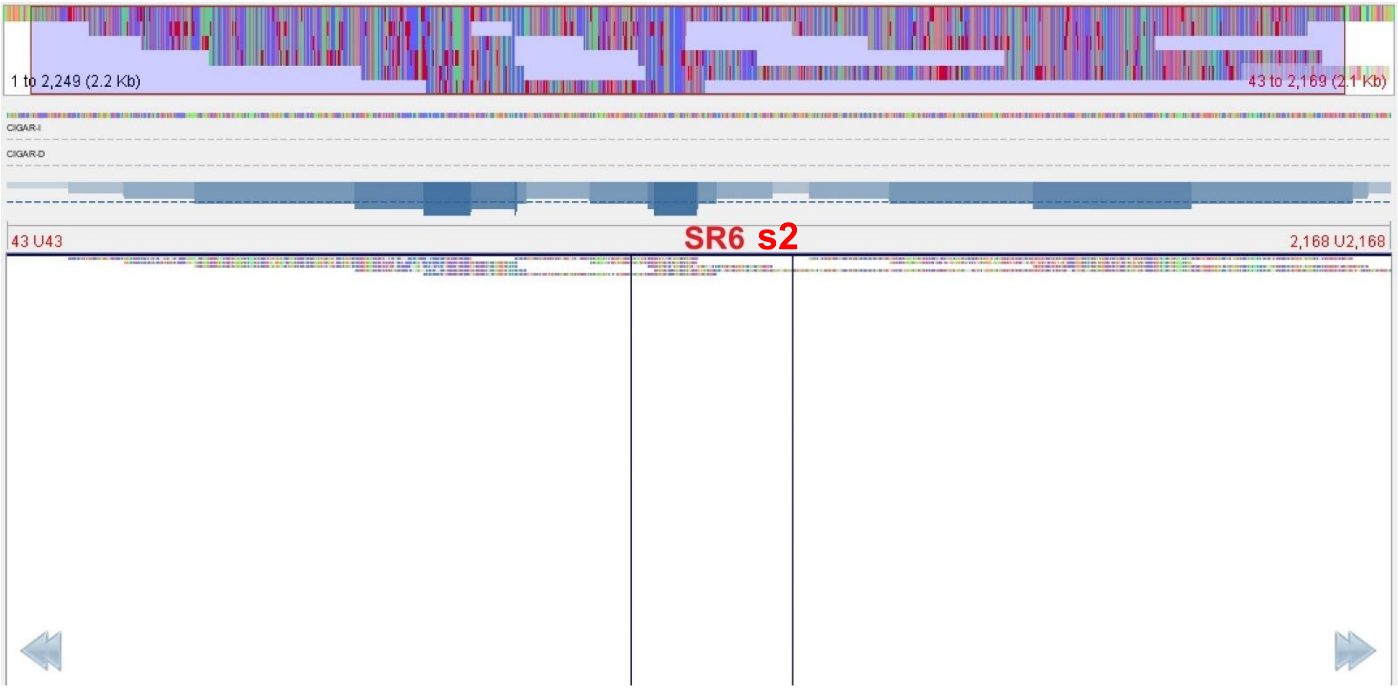
1 to 2,249 (2.2 Kb)
43 to 2,169 (2.1 Kb)
CIGAR-I
CIGAR-D
43 U43
SR6 s2
2,168 U2,168

J

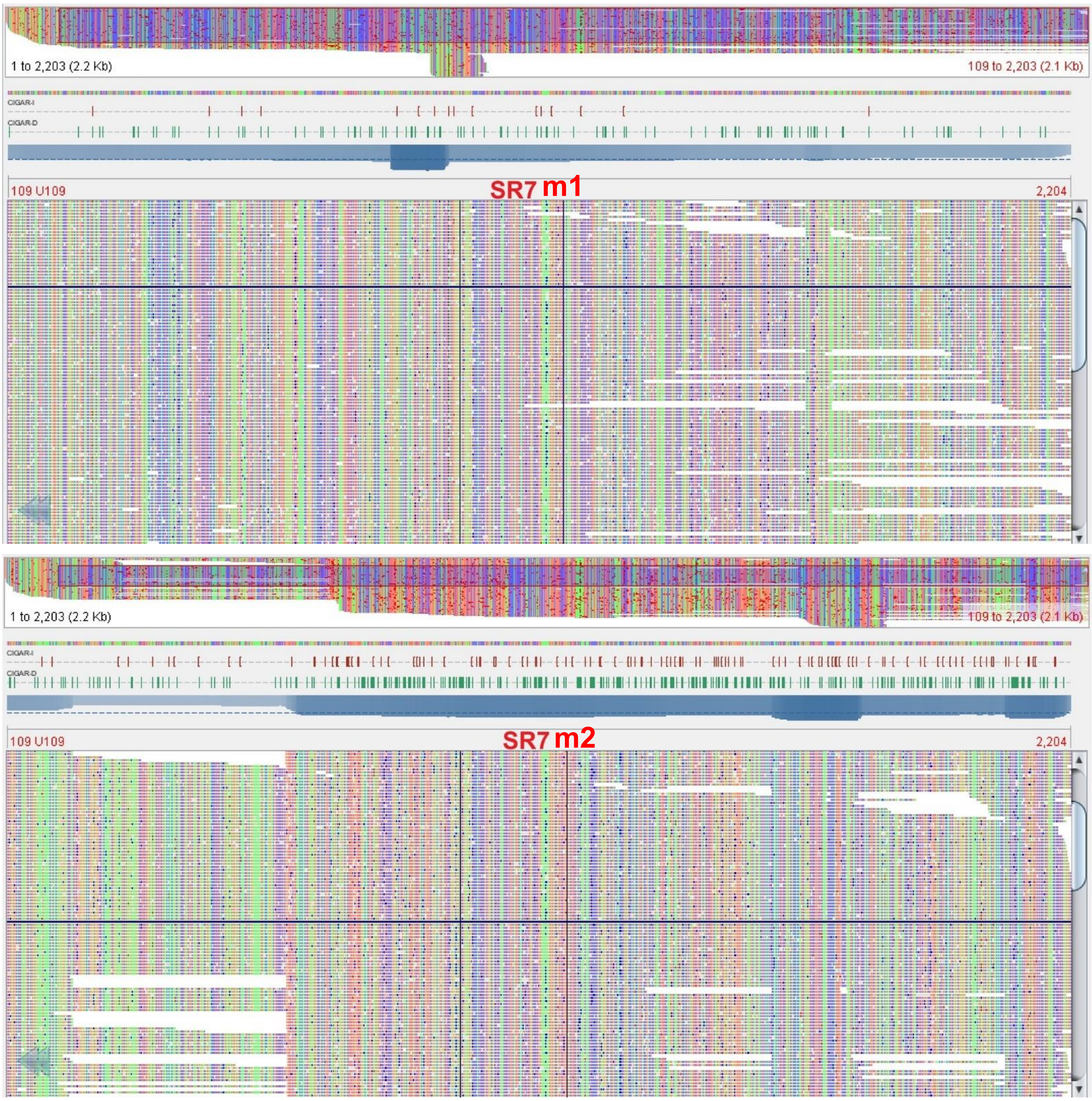
1 to 2,203 (2.2 Kb)
109 to 2,203 (2.1 Kb)
CIGAR-I
CIGAR-D
109 U109
SR7 m1
2,204
1 to 2,203 (2.2 Kb)
109 to 2,203 (2.1 Kb)
CIGAR-I
CIGAR-D
109 U109
SR7 m2
2,204

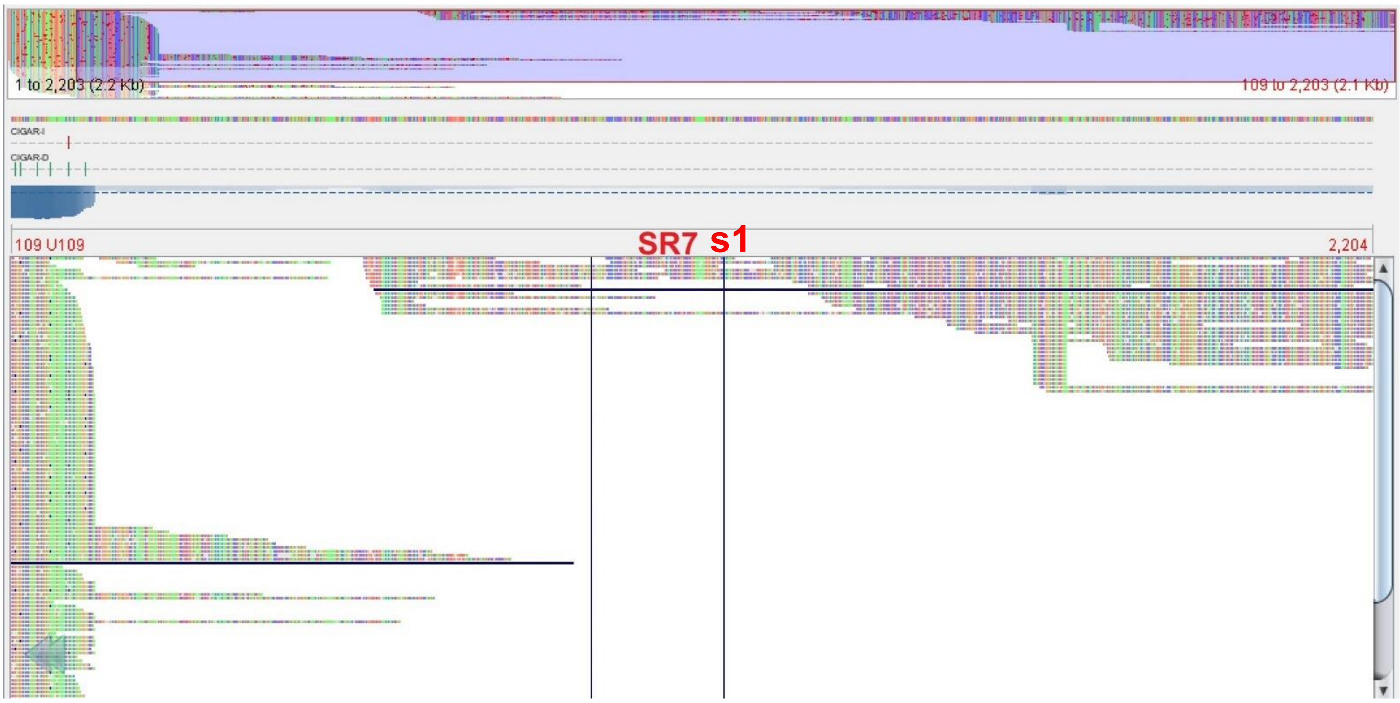
1 to 2,203 (2.2 Kb)
109 to 2,203 (2.1 Kb)
CIGAR-I
CIGAR-D
109 U109
SR7 s1
2,204

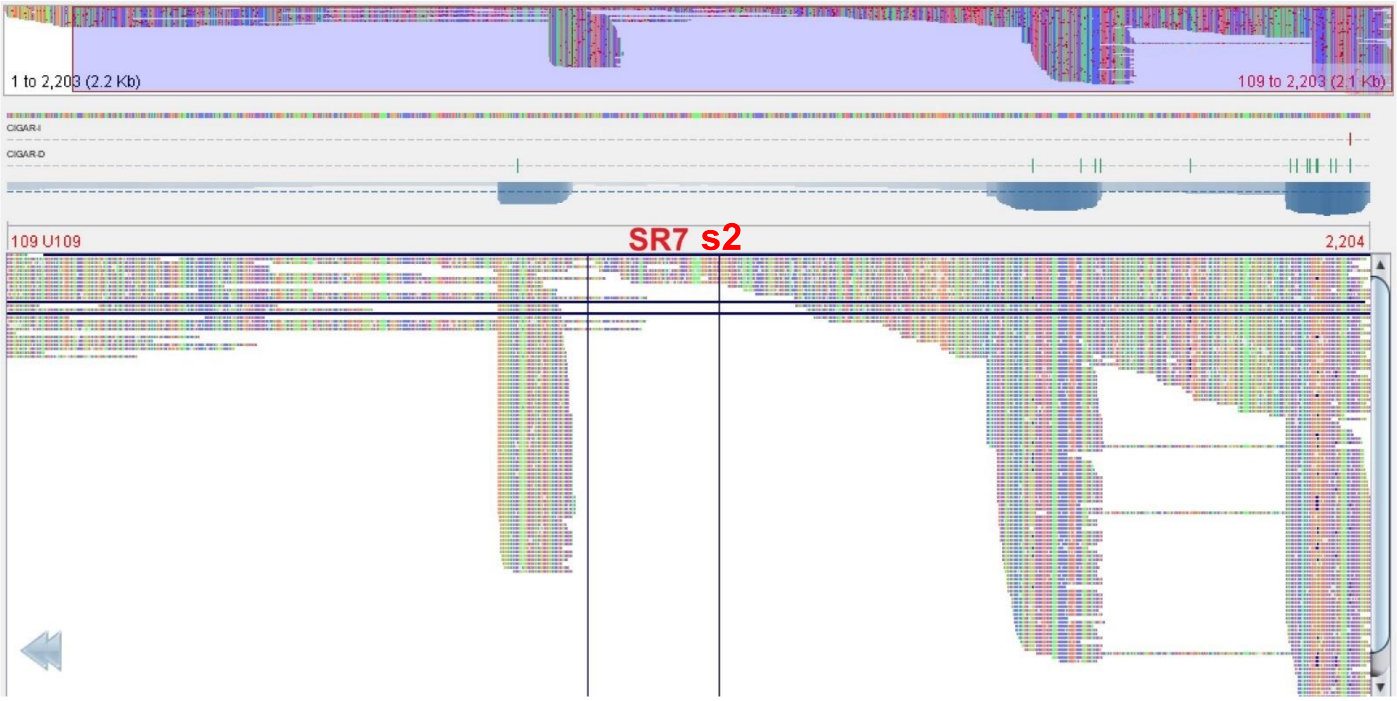
1 to 2,203 (2.2 Kb)
109 to 2,203 (2.1 Kb)
CIGAR-I
CIGAR-D
109 U109
SR7 s2
2,204

K

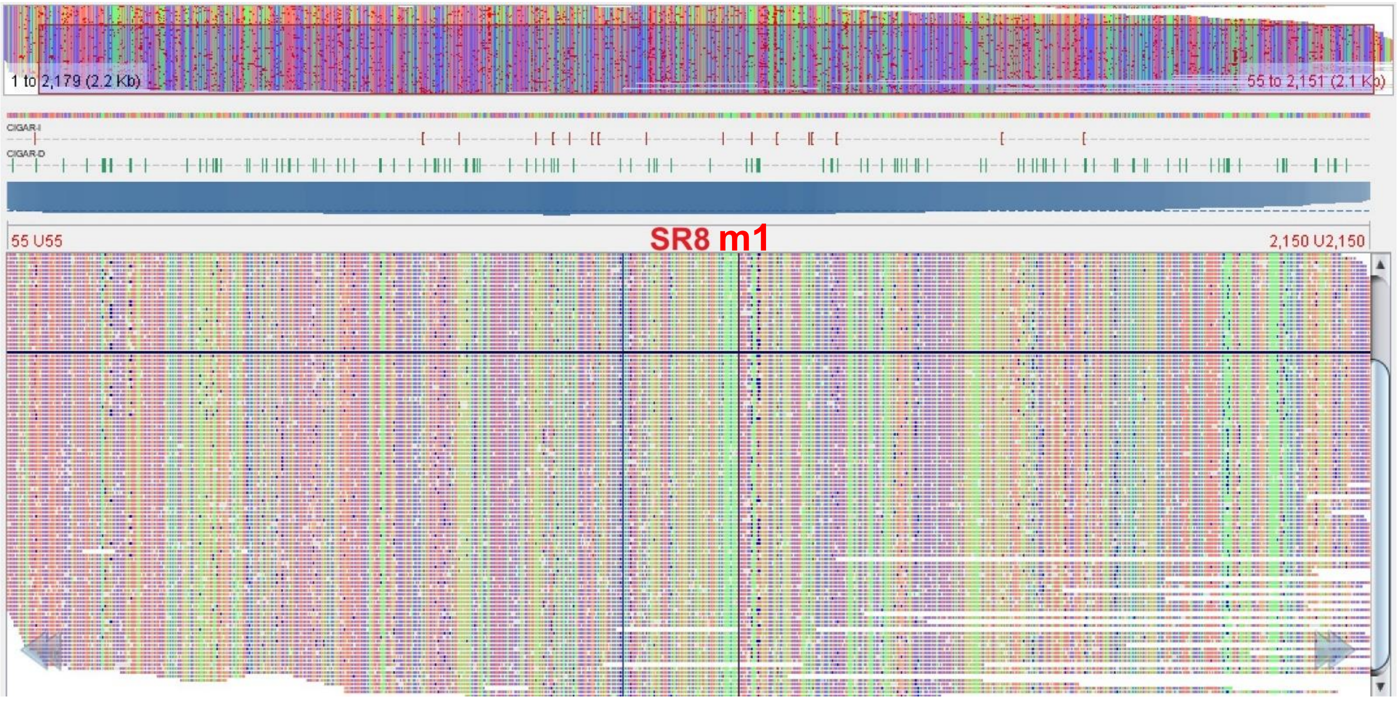
1 to 2,179 (2.2 Kb)
55 to 2,151 (2.1 Kb)
CIGAR-I
CIGAR-D
55 U55
SR8 m1
2,150 U2,150

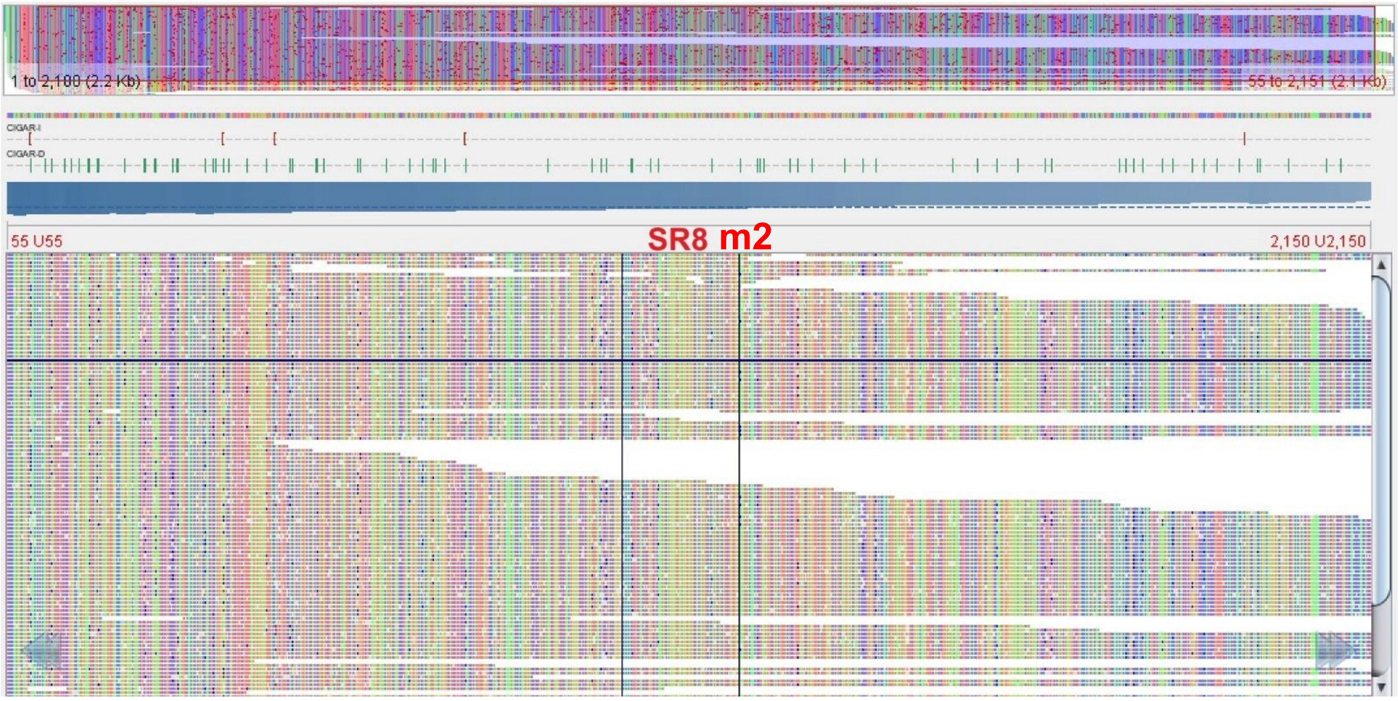
1 to 2,180 (2.2 Kb)
55 to 2,151 (2.1 Kb)
CIGAR-I
CIGAR-D
55 U55
SR8 m2
2,150 U2,150

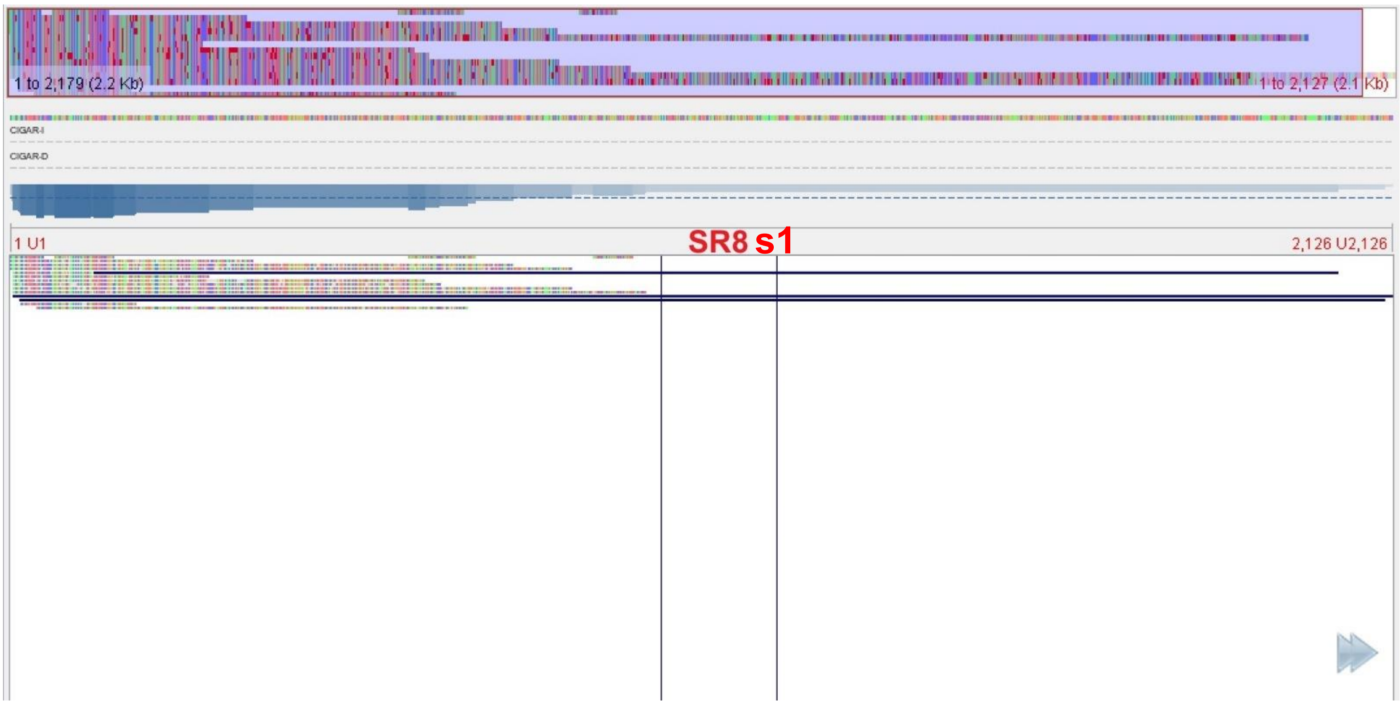
1 to 2,179 (2.2 Kb)
1 to 2,127 (2.1 Kb)
CIGAR-I
CIGAR-D
1 U1
SR8 s1
2,126 U2,126

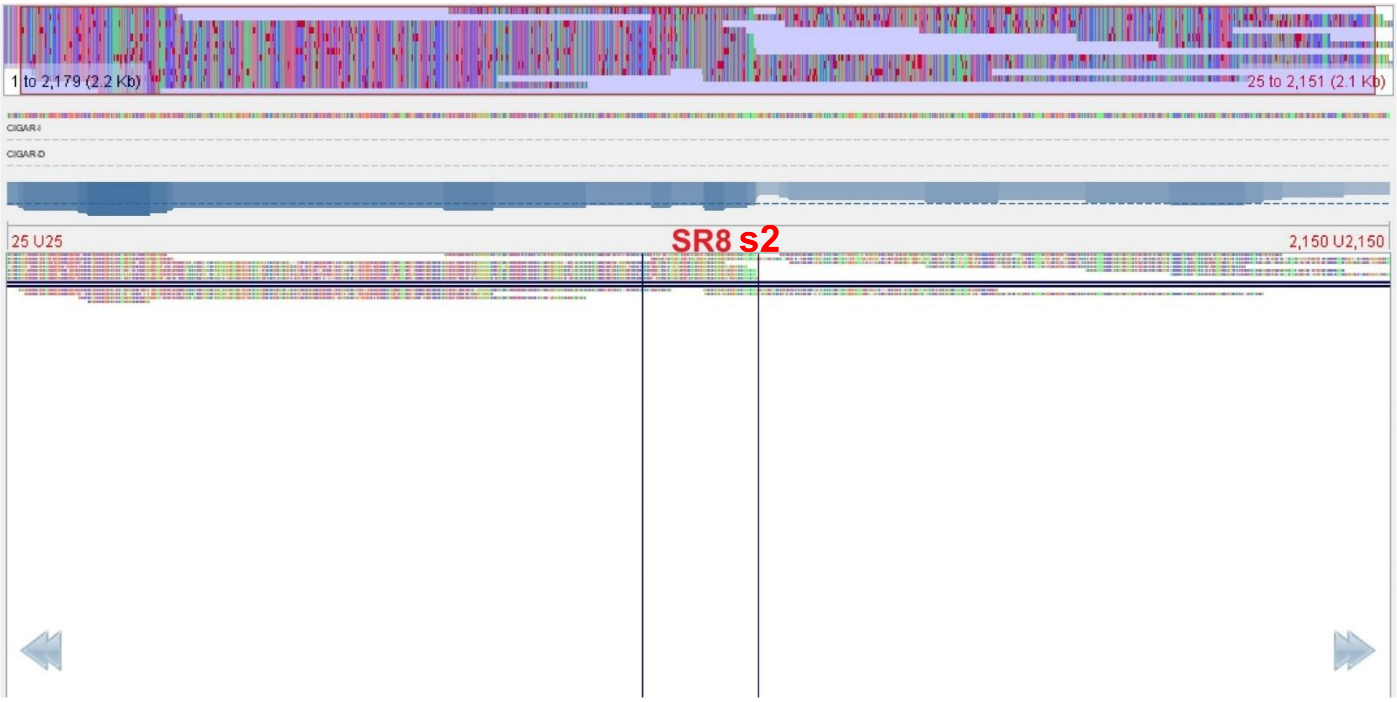
1 to 2,179 (2.2 Kb)
25 to 2,151 (2.1 Kb)
CIGAR-I
CIGAR-D
25 U25
SR8 s2
2,150 U2,150

L

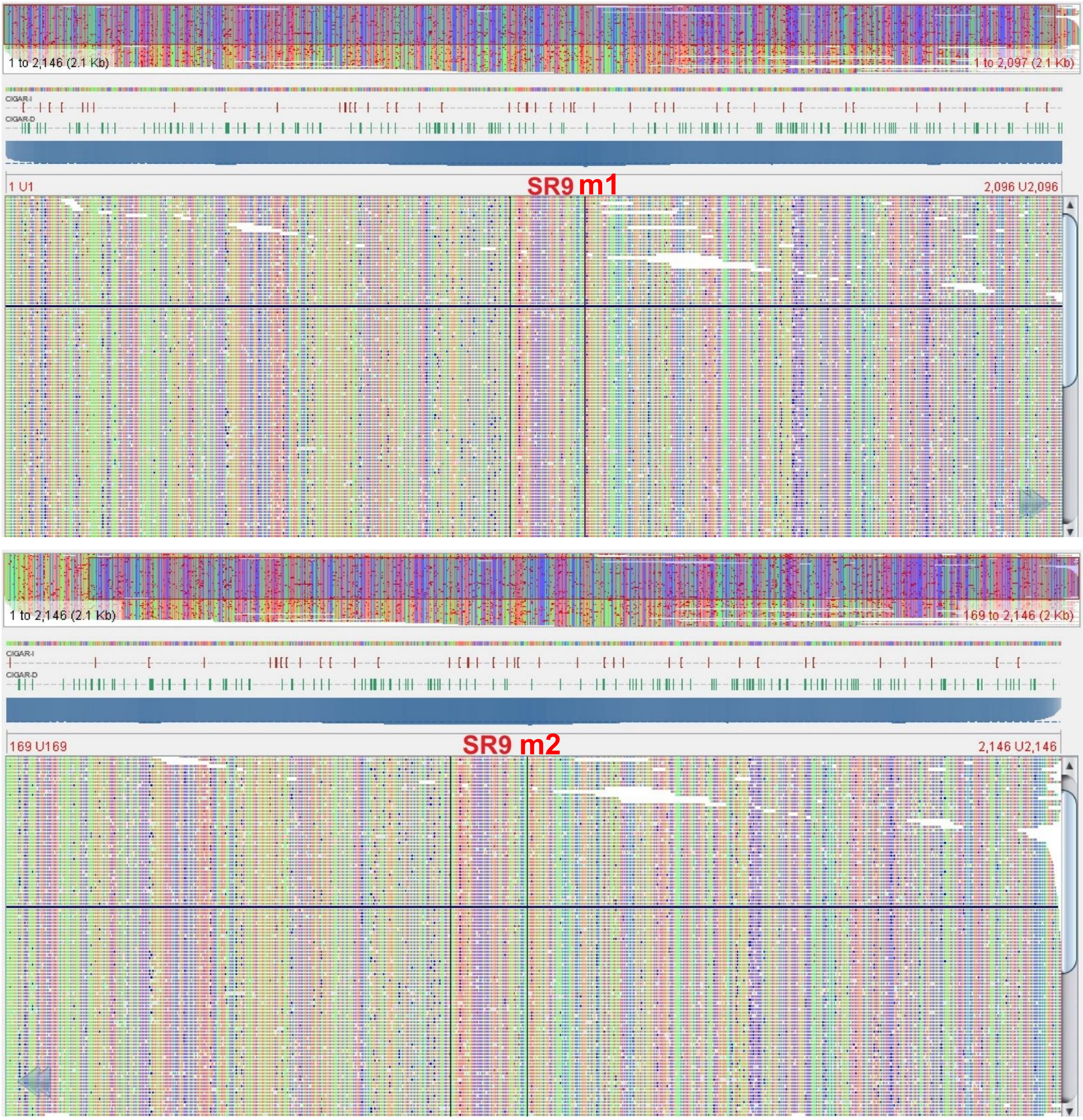
1 to 2,146 (2.1 Kb)
1 to 2,097 (2.1 Kb)
CIGAR-I
CIGAR-D
1 U1
SR9 m1
2,096 U2,096
1 to 2,146 (2.1 Kb)
169 to 2,146 (2 Kb)
CIGAR-I
CIGAR-D
169 U169
SR9 m2
2,146 U2,146

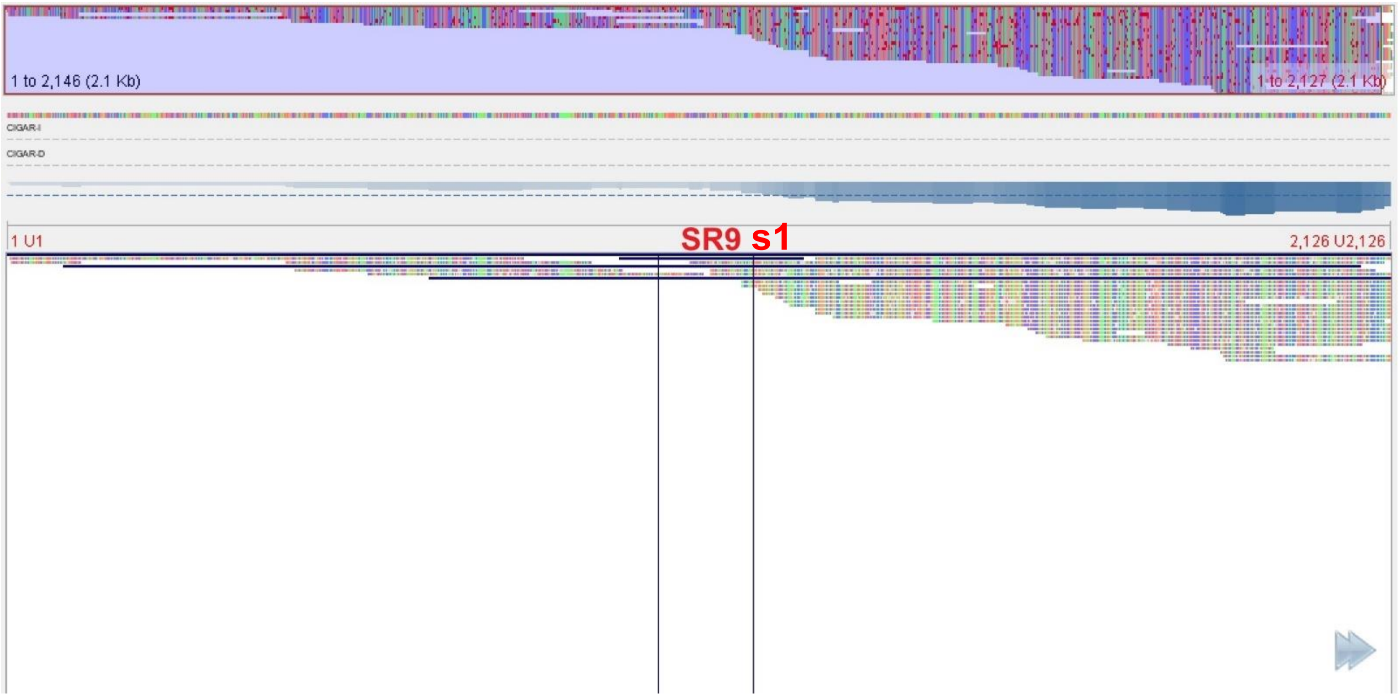
1 to 2,146 (2.1 Kb)
1 to 2,127 (2.1 Kb)
CIGAR-I
CIGAR-D
1 U1
SR9 s1
2,126 U2,126

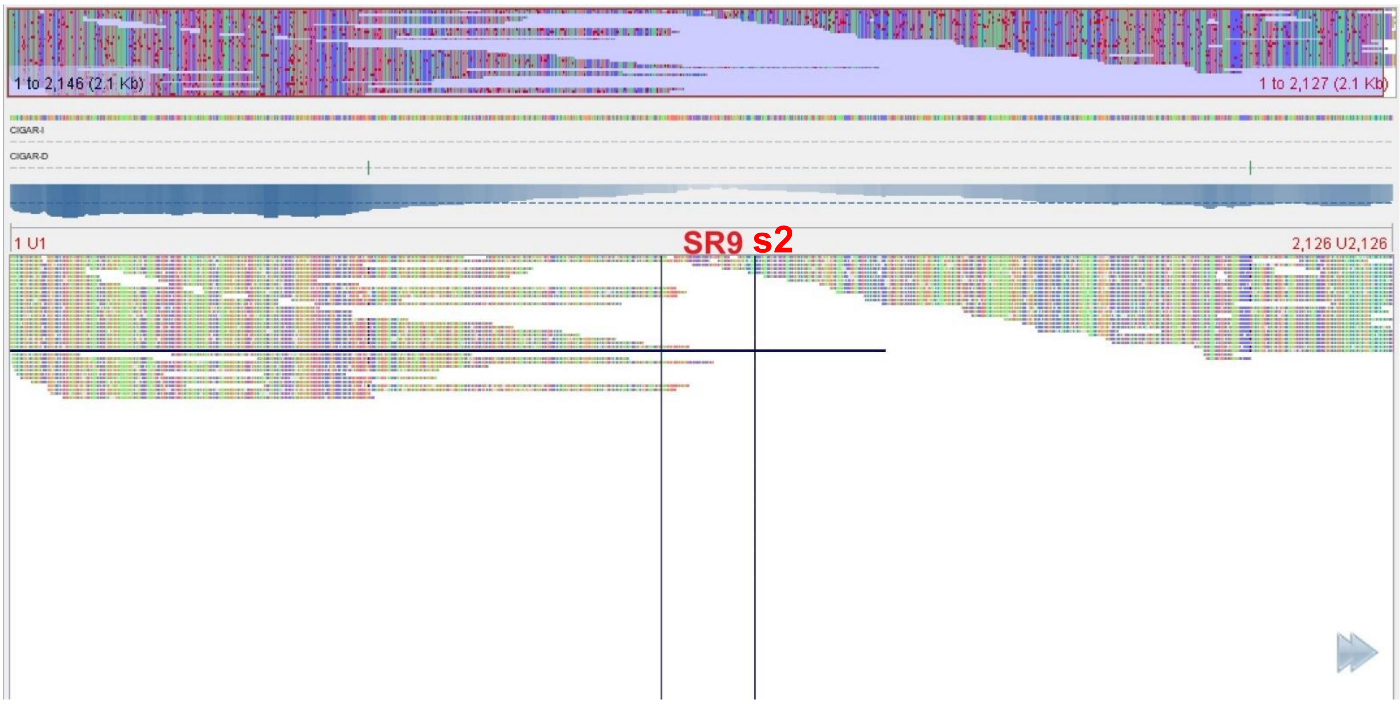
1 to 2,146 (2.1 Kb)
1 to 2,127 (2.1 Kb)
CIGAR-I
CIGAR-D
1 U1
SR9 s2
2,126 U2,126

M

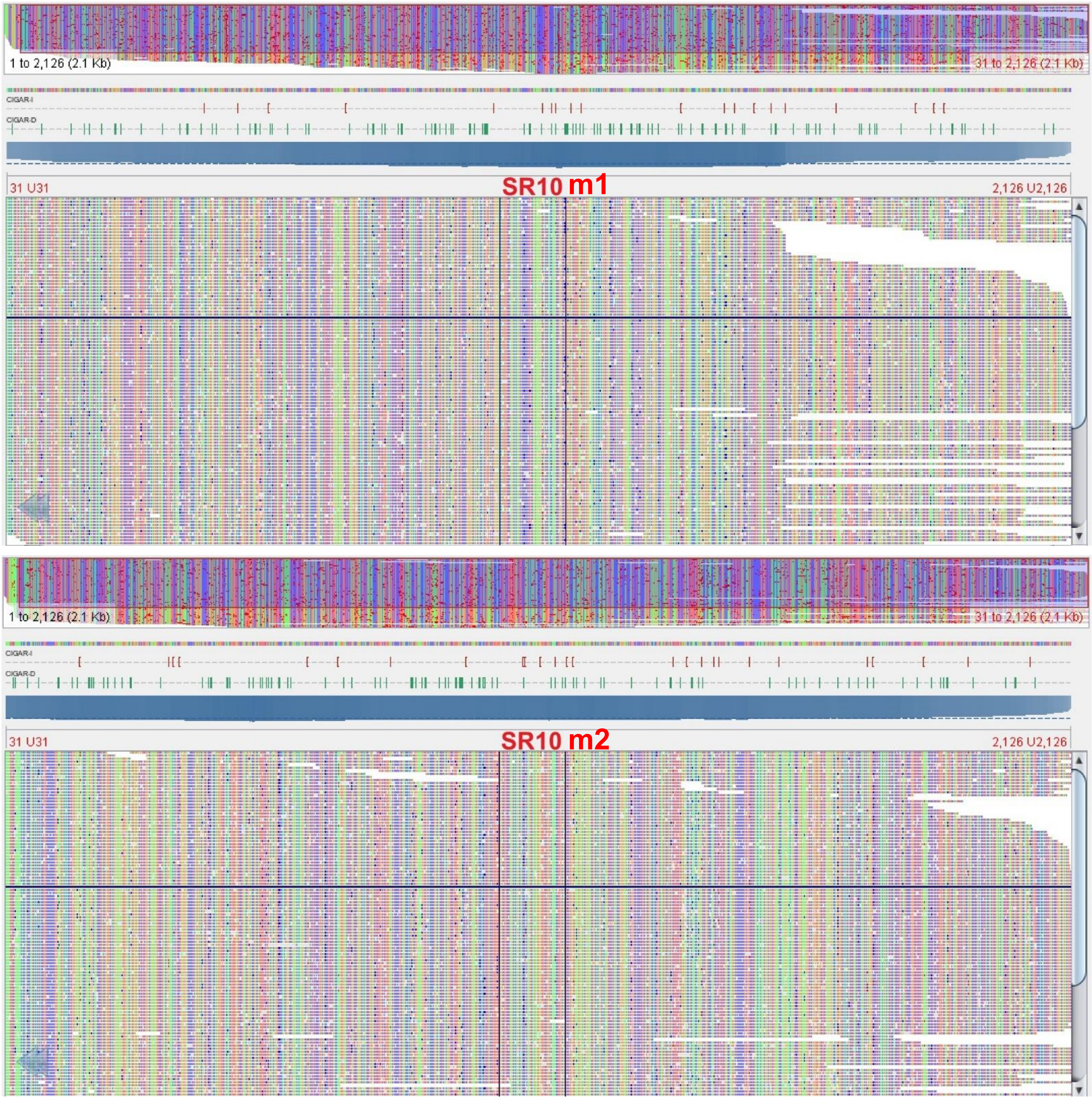
1 to 2,126 (2.1 Kb)
31 to 2,126 (2.1 Kb)
CIGAR-I
CIGAR-D
31 U31
SR10 m1
2,126 U2,126
1 to 2,126 (2.1 Kb)
31 to 2,126 (2.1 Kb)
CIGAR-I
CIGAR-D
31 U31
SR10 m2
2,126 U2,126

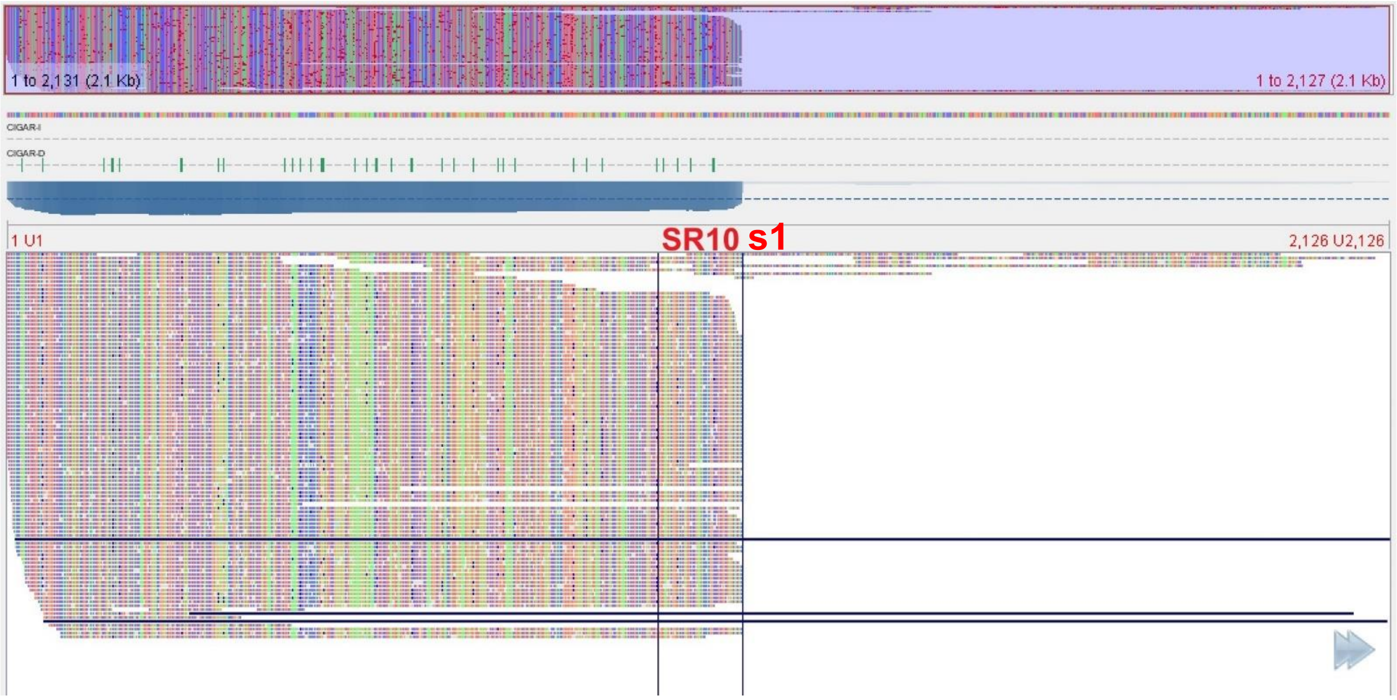
1 to 2,131 (2.1 Kb)
1 to 2,127 (2.1 Kb)
CIGAR-I
CIGAR-D
1 U1
SR10 s1
2,126 U2,126

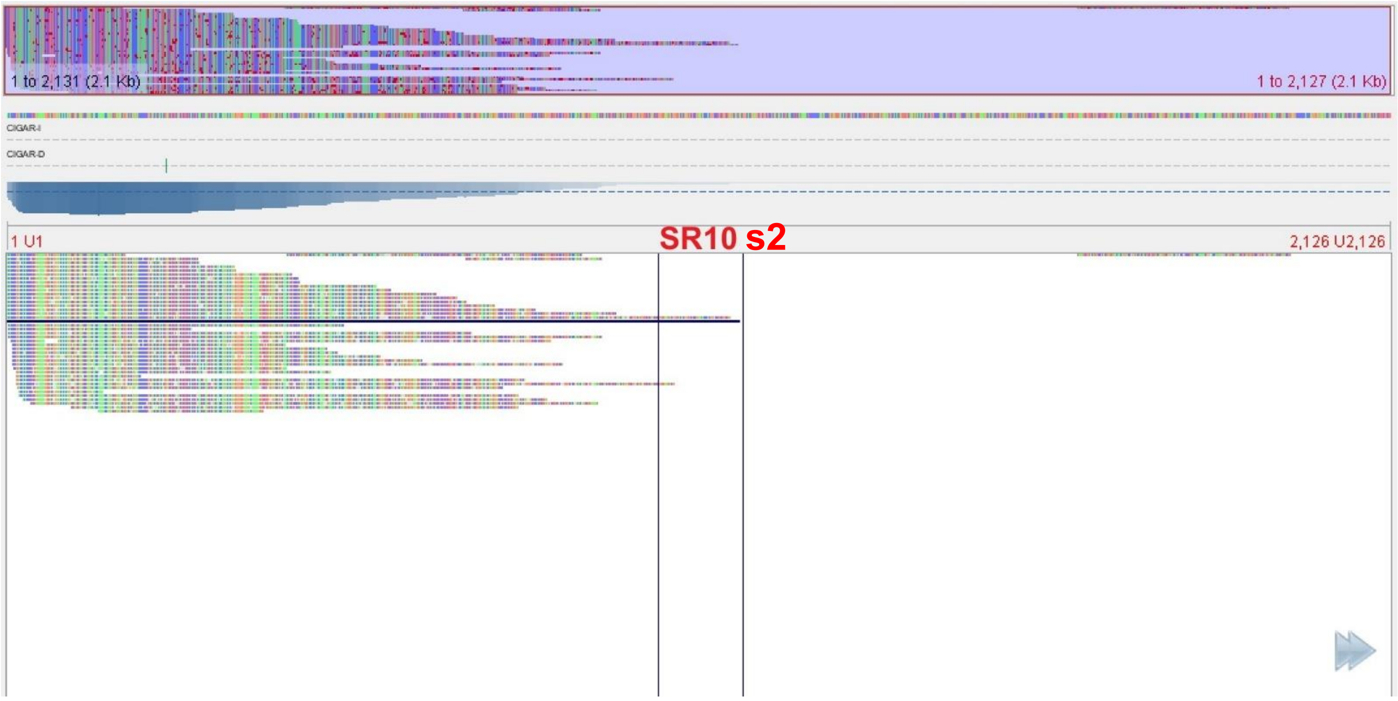
1 to 2,131 (2.1 Kb)
1 to 2,127 (2.1 Kb)
CIGAR-I
CIGAR-D
1 U1
SR10 s2
2,126 U2,126

N

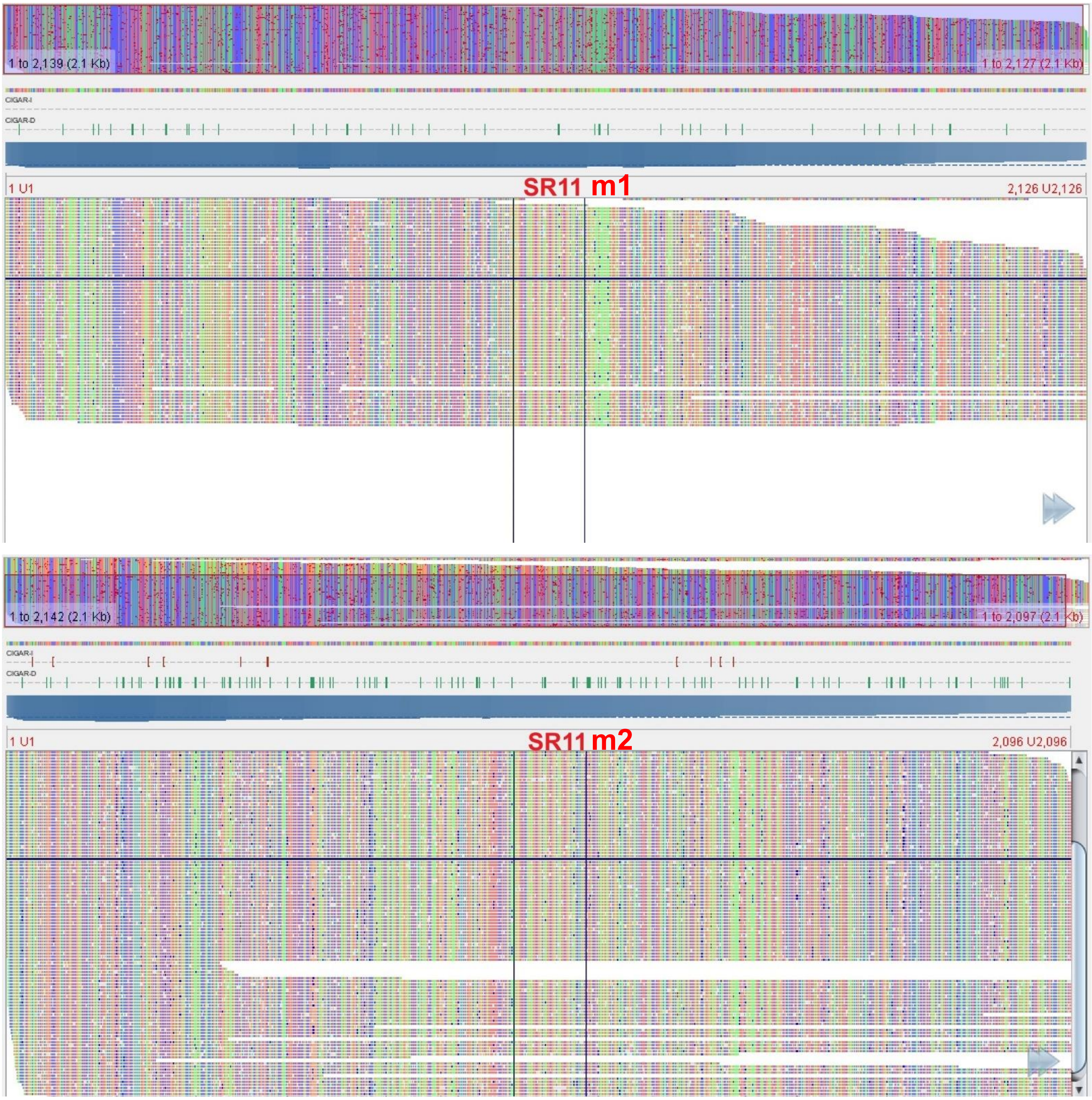
1 to 2,139 (2.1 Kb)
1 to 2,127 (2.1 Kb)
CIGAR-I
CIGAR-D
1 U1
SR11 m1
2,126 U2,126
1 to 2,142 (2.1 Kb)
1 to 2,097 (2.1 Kb)
CIGAR-I
CIGAR-D
1 U1
SR11 m2
2,096 U2,096

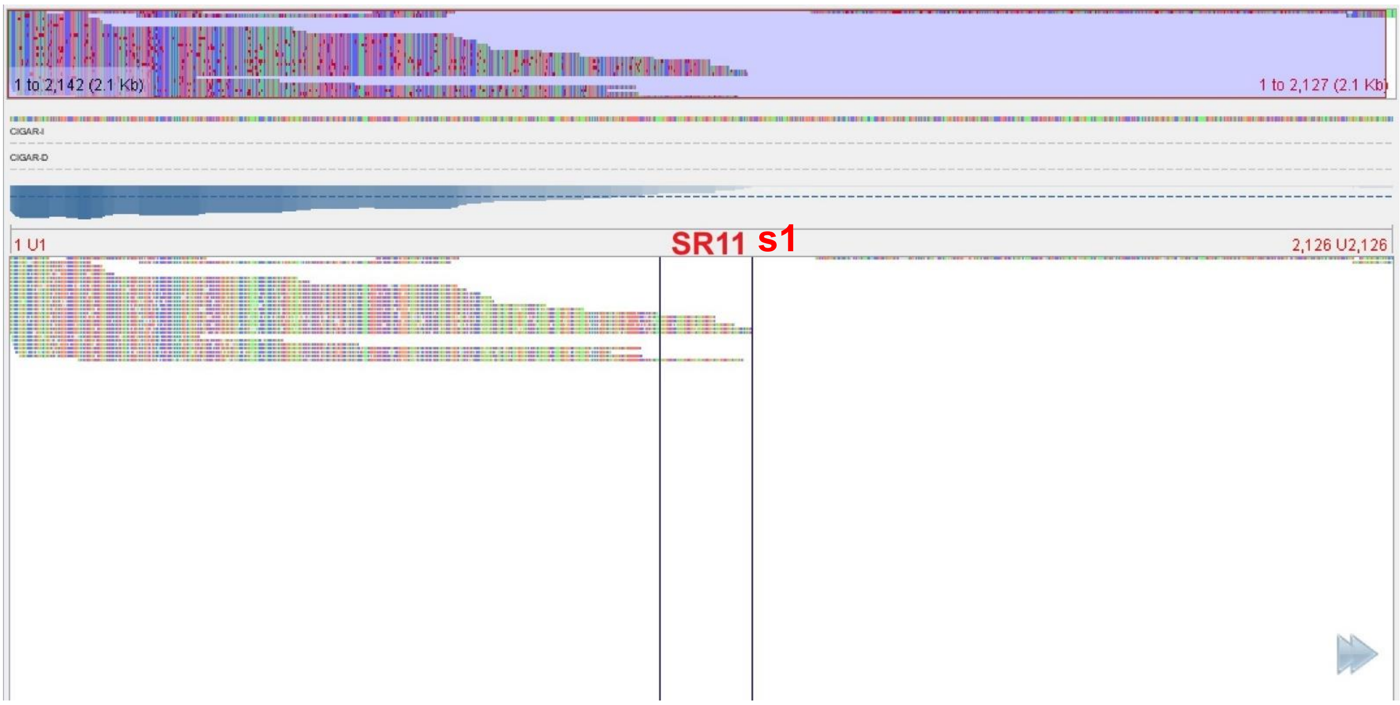
1 to 2,142 (2.1 Kb)
1 to 2,127 (2.1 Kb)
CIGAR-I
CIGAR-D
1 U1
SR11 s1
2,126 U2,126

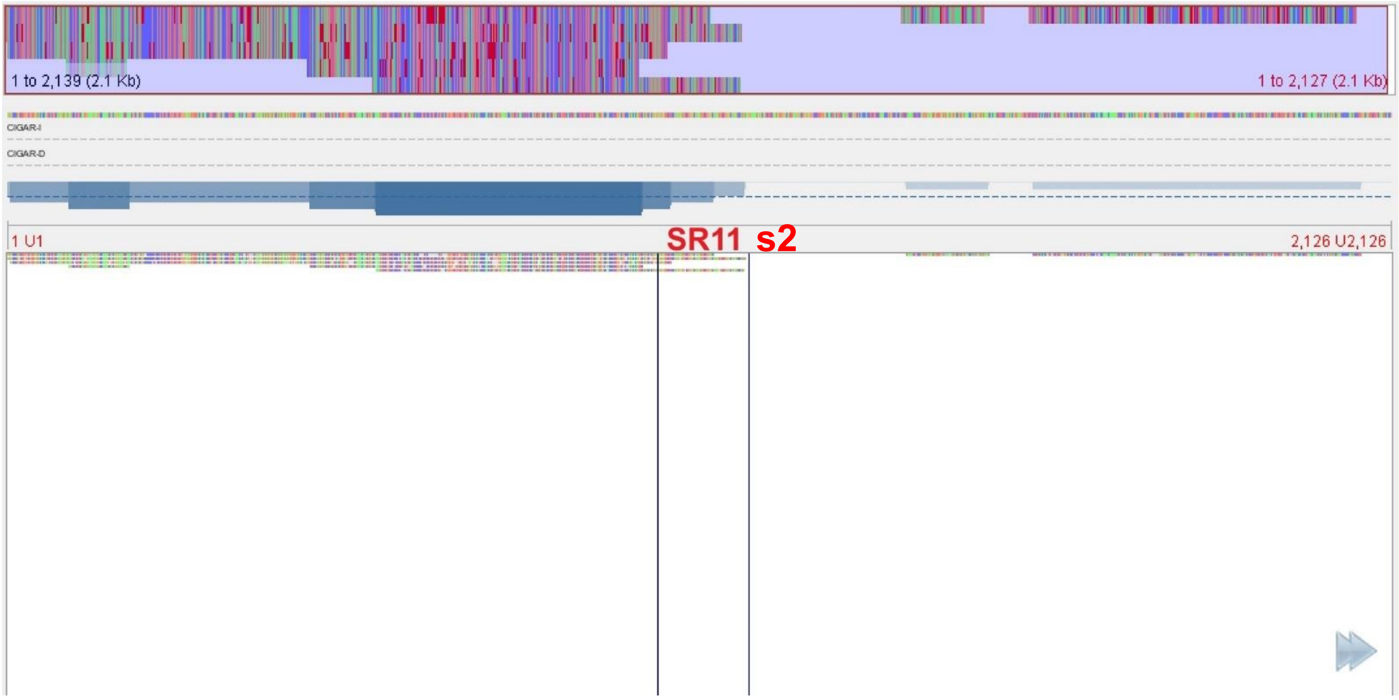
1 to 2,139 (2.1 Kb)
1 to 2,127 (2.1 Kb)
CIGAR-I
CIGAR-D
1 U1
SR11 s2
2,126 U2,126

O

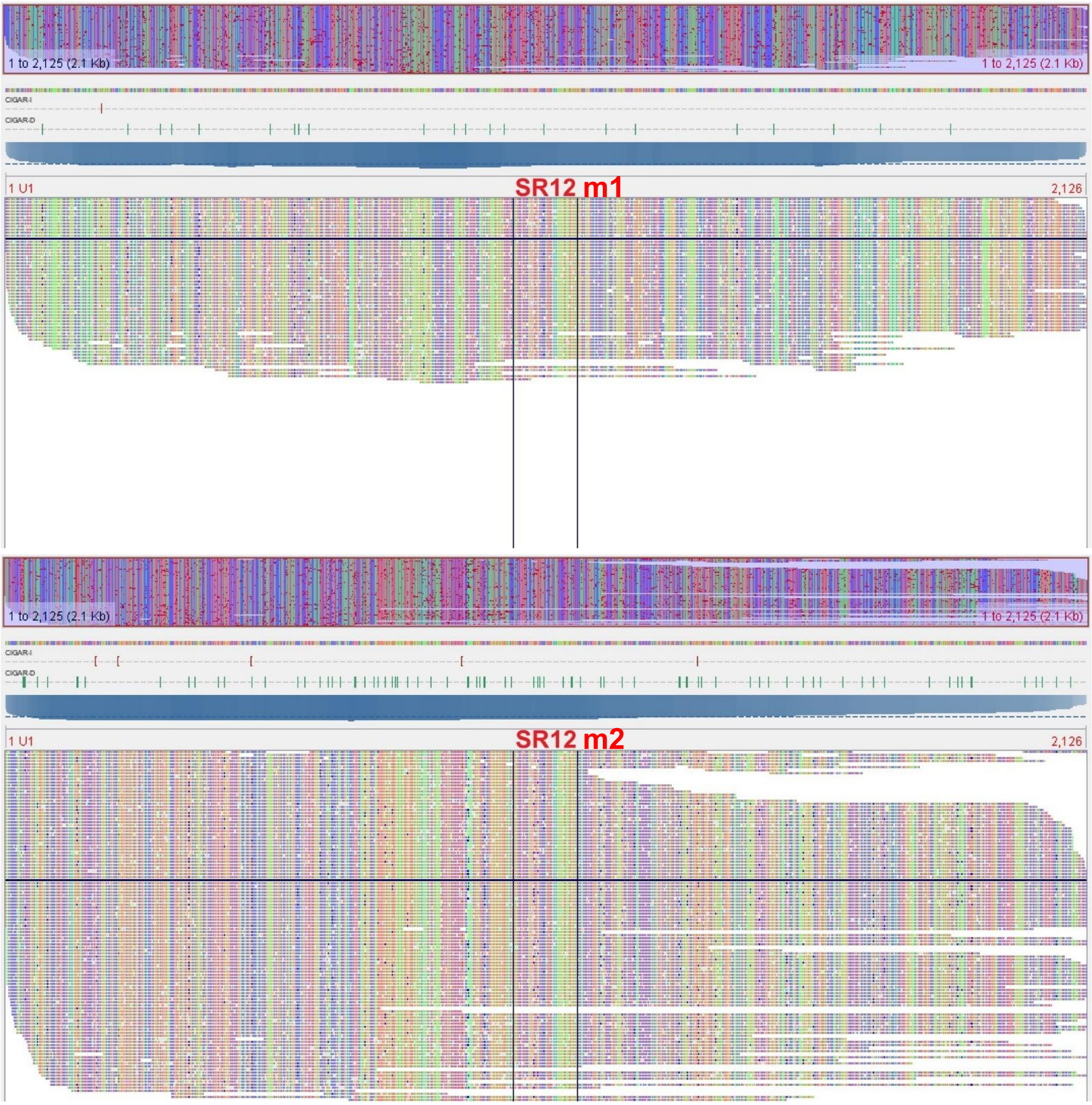
1 to 2,125 (2.1 Kb)
1 to 2,125 (2.1 Kb)
CIGAR-I
CIGAR-D
1 U1
SR12 m1
2,126
1 to 2,125 (2.1 Kb)
1 to 2,125 (2.1 Kb)
CIGAR-I
CIGAR-D
1 U1
SR12 m2
2,126

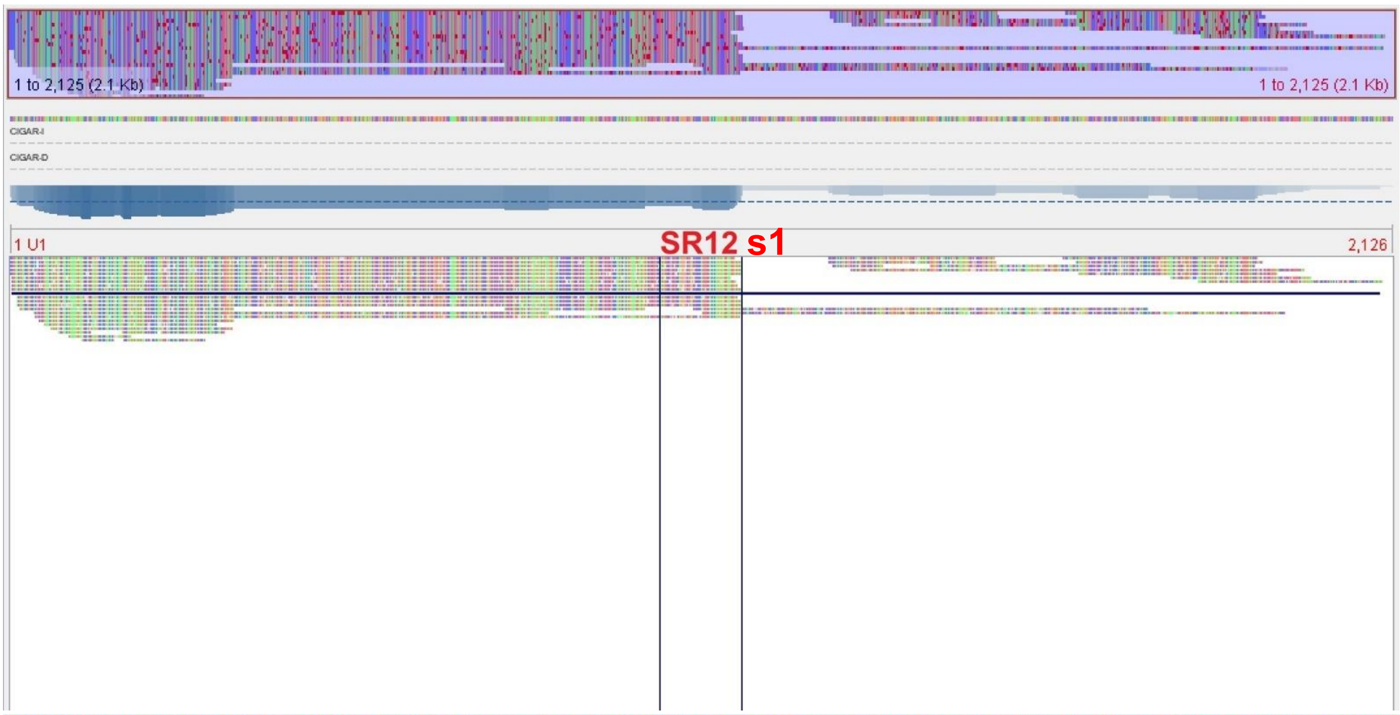
1 to 2,125 (2.1 Kb)
1 to 2,125 (2.1 Kb)
CIGAR-I
CIGAR-D
1 U1
SR12 s1
2,126

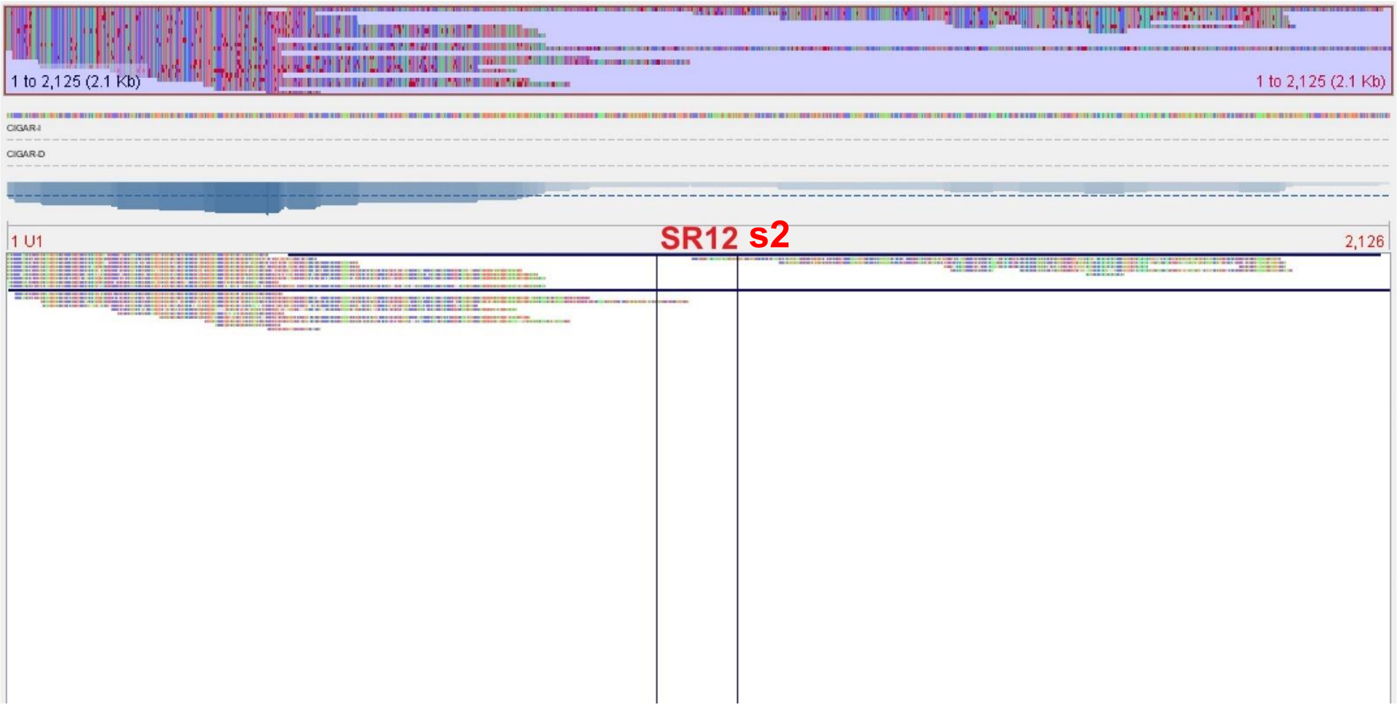
1 to 2,125 (2.1 Kb)
1 to 2,125 (2.1 Kb)
CIGAR-I
CIGAR-D
1 U1
SR12 s2
2,126

Figure S3. The sequencing depth of two chromosomes of mt DNA mapped by Illumina short-reads and Nanopore long reads. **A** and **B** showed the sequencing depth for chr1 and chr2 using the short-reads data. **C** and **D** showed the sequencing depth for chr1 and chr2 using the long-reads data.

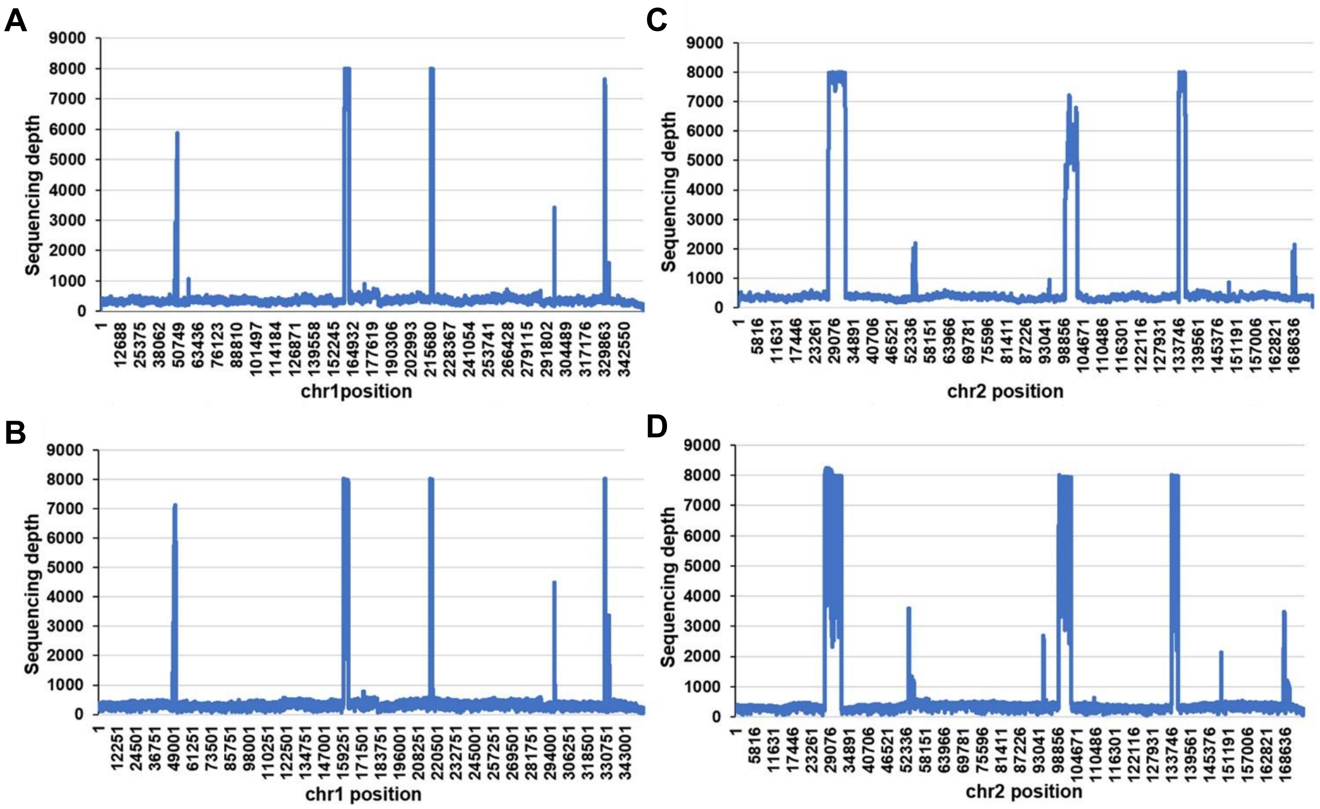

Figure S4. Identification of migrated sequences from cpDNA in okra's mtDNA. We have marked the representative long reads in dark blue, which was flanked by mtDNA, and contained sequences migrating from chloroplasts (MTPT) in the middle. This suggesting that these sequences, although highly similar to cpDNA, have been integrated into mtDNA. While typical reads from cpDNA only had the regions of MTPT could be mapped to the mitochondrial genome. At the top of each subgraph, the overall mapping is marked in red frame, and most reads were from cpDNA. The last subgraph showed the reads mapping of mtpt14 and mtpt16, and some regions were not covered by cpDNA reads, indicating that mtpt14 of mtDNA differs from cpDNA in sequence to some extent.

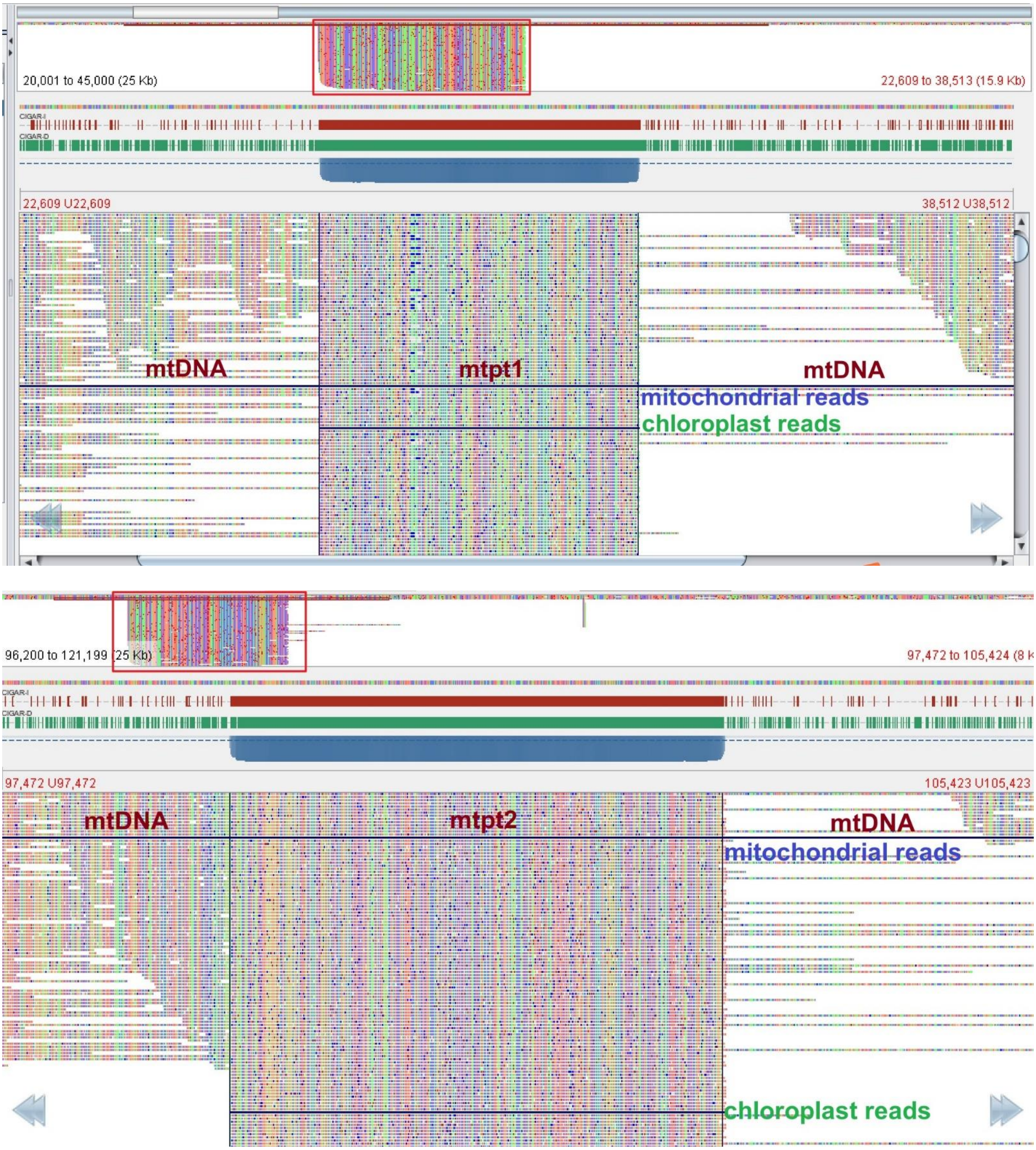

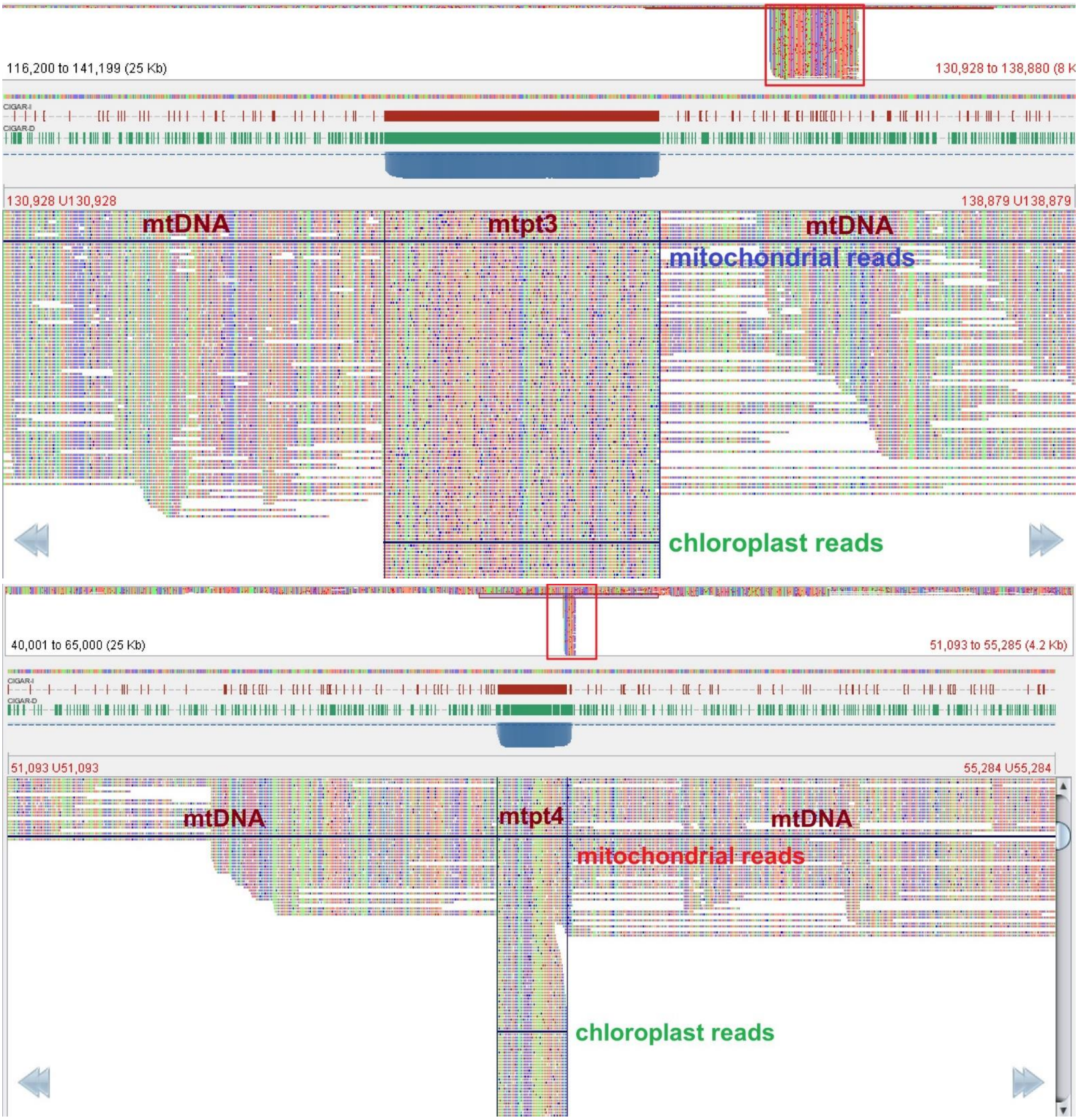
116,200 to 141,199 (25 Kb)
130,928 to 138,880 (8 K
CIGAR-I
CIGAR-D
130,928 U130,928
138,879 U138,879
mtDNA
mtpt3
mtDNA
mitochondrial reads
chloroplast reads
40,001 to 65,000 (25 Kb)
51,093 to 55,285 (4.2 Kb)
CIGAR-I
CIGAR-D
51,093 U51,093
55,284 U55,284
mtDNA
mtpt4
mtDNA
mitochondrial reads
chloroplast reads

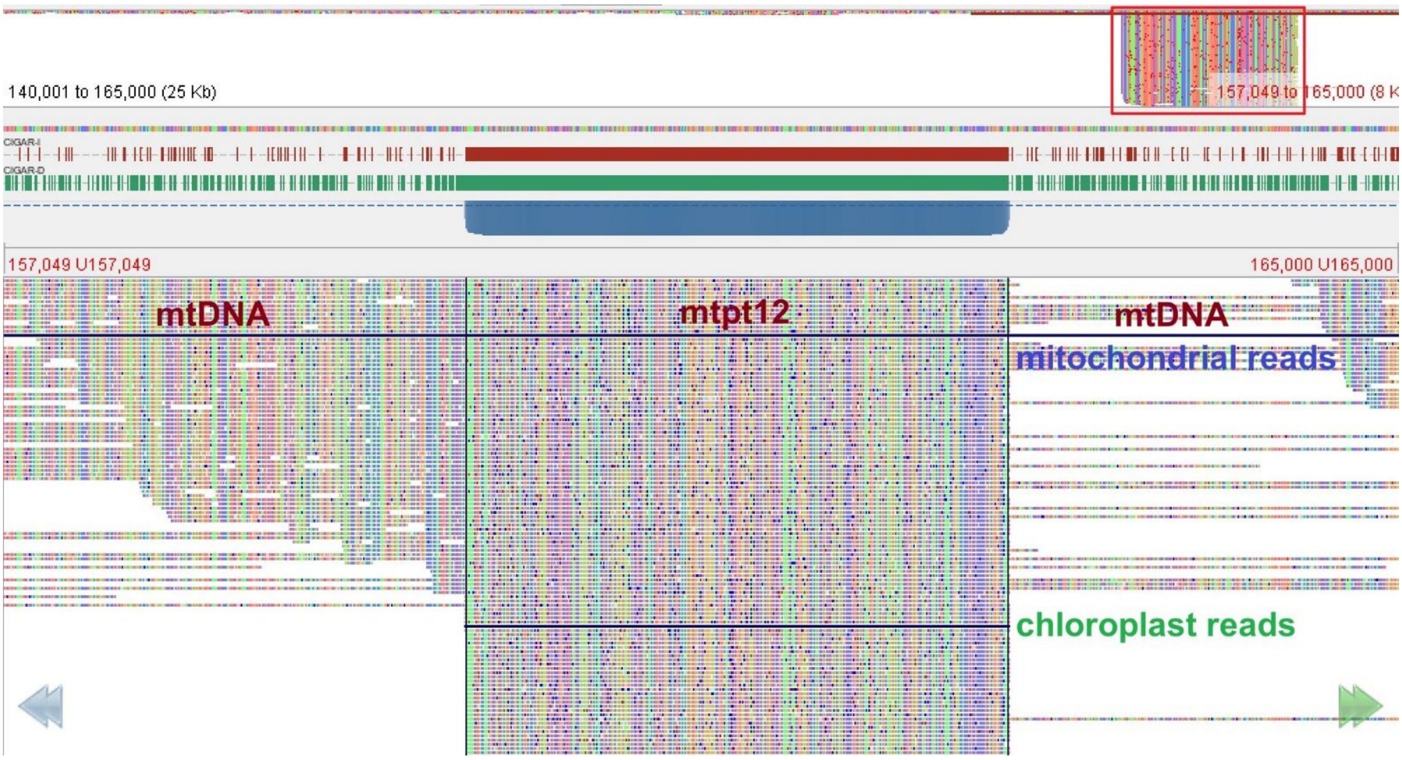
140,001 to 165,000 (25 Kb)
157,049 to 165,000 (8 K
CIGAR-I
CIGAR-D
157,049 U157,049
165,000 U165,000
mtDNA
mtpt12
mtDNA
mitochondrial reads
chloroplast reads

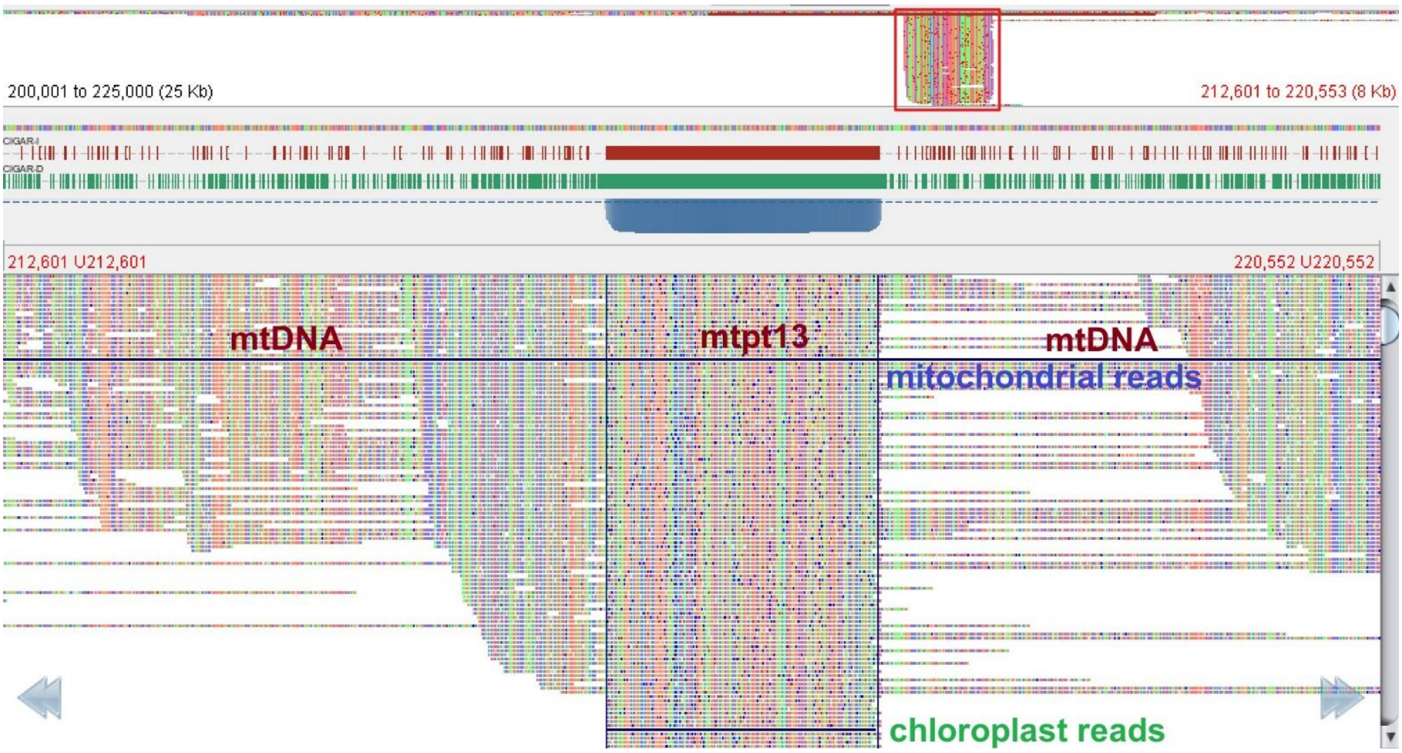
200,001 to 225,000 (25 Kb)
212,601 to 220,553 (8 Kb)
CIGAR-I
CIGAR-D
212,601 U212,601
220,552 U220,552
mtDNA
mtpt13
mtDNA
mitochondrial reads
chloroplast reads

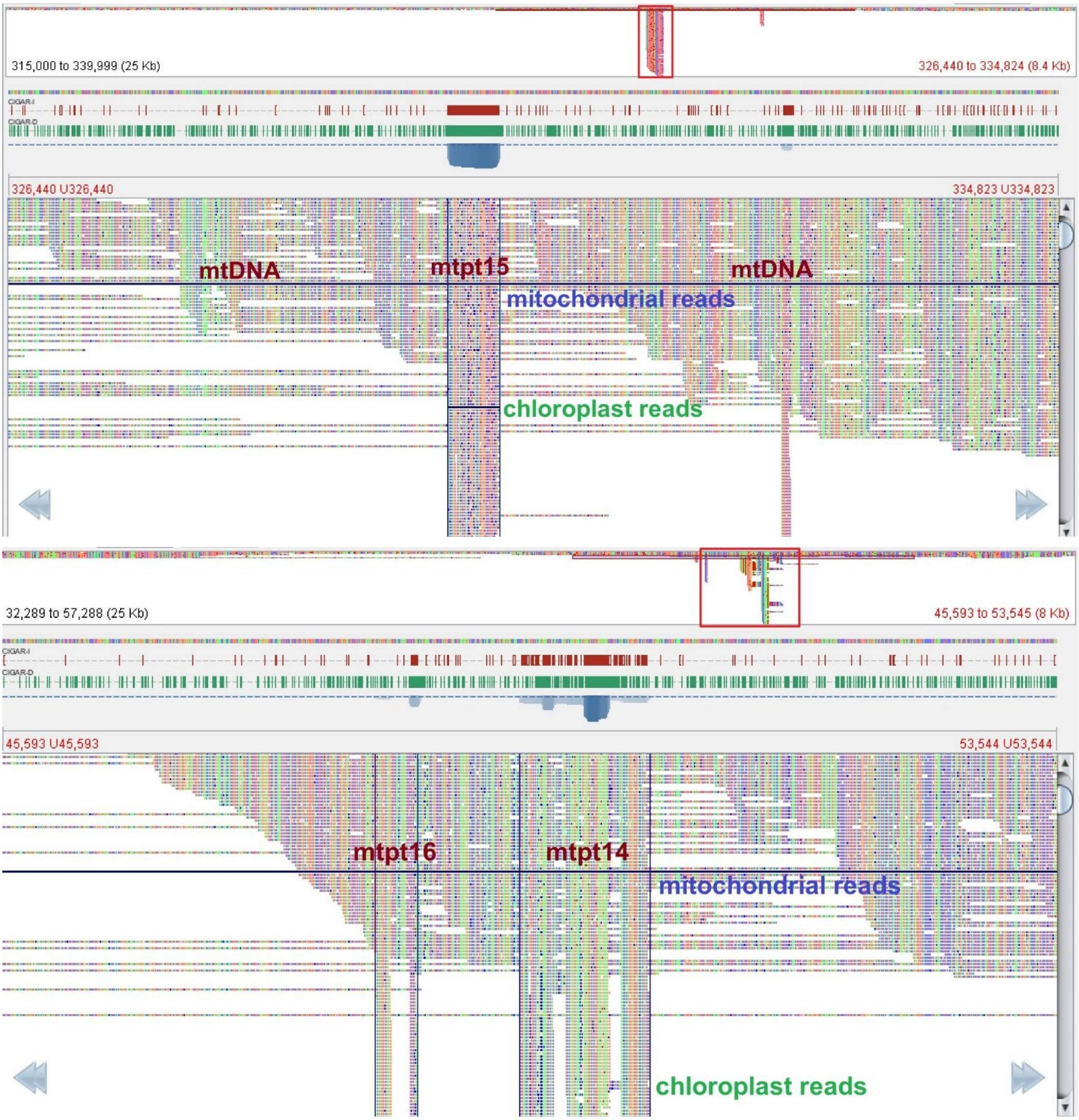
315,000 to 339,999 (25 Kb)
326,440 to 334,824 (8.4 Kb)
CIGAR-I
CIGAR-D
326,440 U326,440
334,823 U334,823
mtDNA
mtpt15
mtDNA
mitochondrial reads
chloroplast reads
32,289 to 57,288 (25 Kb)
45,593 to 53,545 (8 Kb)
CIGAR-I
CIGAR-D
45,593 U45,593
53,544 U53,544
mtpt16
mtpt14
mitochondrial reads
chloroplast reads

Figure S5. Alignment of mtpt14 identified in cpDNA and mtDNA. These homologous sequences contained 4 genes/pseudogenes.

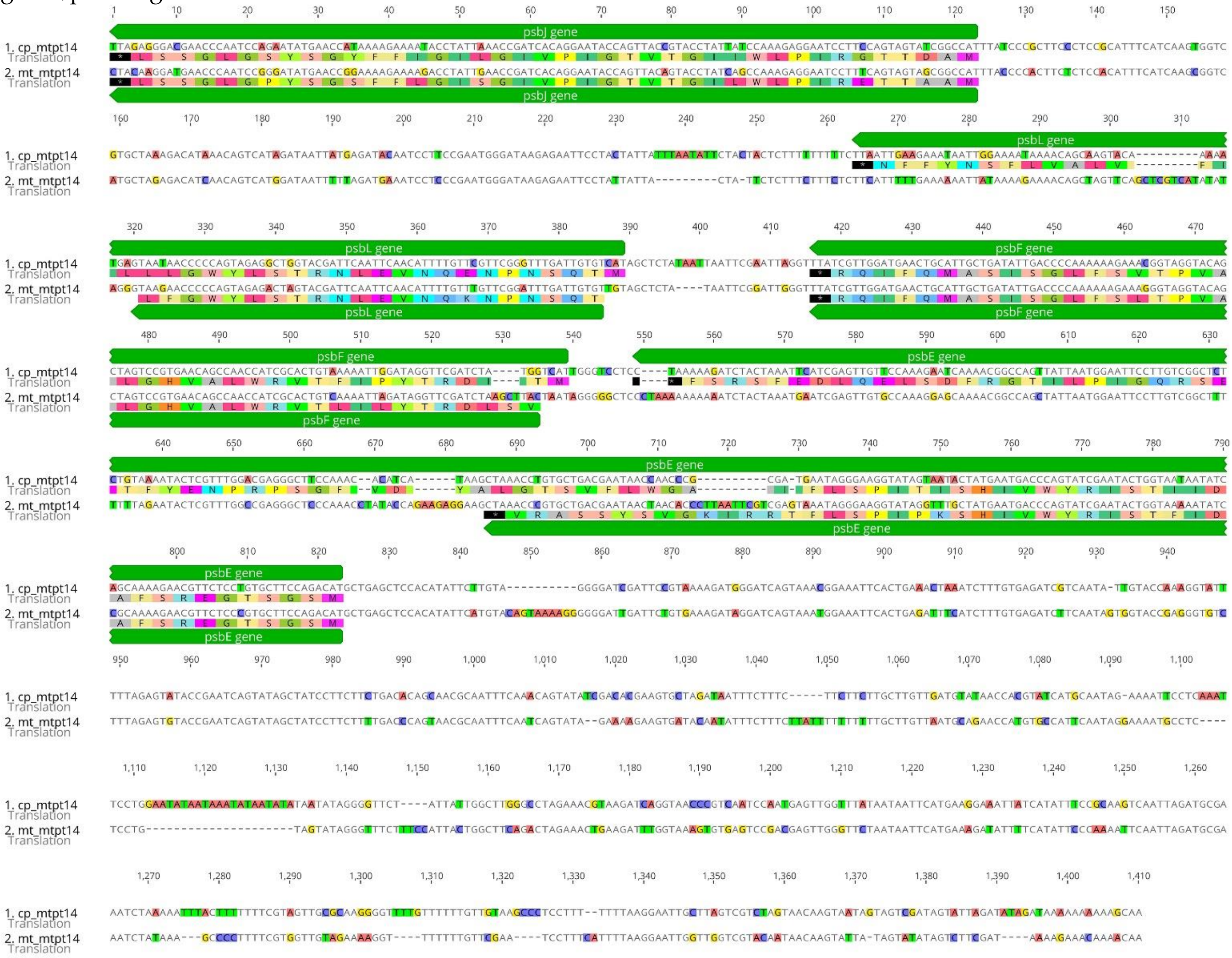

Table S1. The BLASTn results searched among the two organelle genomes.

Query	Subject	Identity (%)	Alignment length	Mismatches	Gap opens	Q. start	Q. end	S. start	S. end	Evalue	Bit score	mtpt#
cpDNA	mtDNA_chr2	99.903	5142	5	0	101579	106720	32524	27383	0	9468	mtpt1
cpDNA	mtDNA_chr2	99.903	5142	5	0	144473	149614	27383	32524	0	9468	mtpt1
cpDNA	mtDNA_chr2	99.583	3833	1	1	40982	44814	103040	99223	0	6975	mtpt2
cpDNA	mtDNA_chr2	100	2039	0	0	142208	144246	135795	133757	0	3766	mtpt3
cpDNA	mtDNA_chr2	100	2039	0	0	106947	108985	133757	135795	0	3766	mtpt3
cpDNA	mtDNA_chr2	98.714	311	4	0	86170	86480	53039	53349	2.43E-156	553	mtpt4
cpDNA	mtDNA_chr2	98.714	311	4	0	86170	86480	168192	168502	2.43E-156	553	mtpt4
cpDNA	mtDNA_chr2	97.619	84	2	0	32029	32112	94520	94437	1.73E-33	145	mtpt7
cpDNA	mtDNA_chr2	93.902	82	4	1	136381	136461	110099	110018	8.13E-27	122	mtpt8
cpDNA	mtDNA_chr2	93.902	82	4	1	114732	114812	110018	110099	8.13E-27	122	mtpt8
cpDNA	mtDNA_chr2	97.015	74	2	0	1	74	149018	148952	4.89E-24	113	mtpt9
cpDNA	mtDNA_chr2	82.54	126	22	0	41419	41544	100379	100254	1.76E-23	111	mtpt5
cpDNA	mtDNA_chr2	82.54	126	22	0	43643	43768	102603	102478	1.76E-23	111	mtpt6
cpDNA	mtDNA_chr2	100	36	0	0	69098	69133	13796	13761	3.86E-10	67.6	mtpt11
cpDNA	mtDNA_chr2	97.436	39	1	0	46790	46828	29944	29906	3.86E-10	67.6	mtpt10
cpDNA	mtDNA_chr2	100	36	0	0	126842	126877	29944	29909	3.86E-10	67.6	mtpt10
cpDNA	mtDNA_chr1	99.968	3114	0	1	150223	153335	162783	159670	0	5744	mtpt12
cpDNA	mtDNA_chr1	99.968	3114	0	1	97858	100970	159670	162783	0	5744	mtpt12
cpDNA	mtDNA_chr1	100	1596	0	0	140003	141598	217672	216077	0	2948	mtpt13
cpDNA	mtDNA_chr1	100	1596	0	0	109595	111190	216077	217672	0	2948	mtpt13
cpDNA	mtDNA_chr1	79.929	1410	179	56	68905	70261	50483	49125	0	941	mtpt14
cpDNA	mtDNA_chr1	96.364	440	4	3	111355	111790	330367	329936	0	713	mtpt15
cpDNA	mtDNA_chr1	96.364	440	4	3	139403	139838	329936	330367	0	713	mtpt15
cpDNA	mtDNA_chr1	79.642	447	53	24	71113	71558	48748	48339	2.72E-76	287	mtpt16
cpDNA	mtDNA_chr1	76.279	430	77	17	143683	144102	38777	38363	7.85E-52	206	mtpt17
cpDNA	mtDNA_chr1	76.279	430	77	17	107091	107510	38363	38777	7.85E-52	206	mtpt17
cpDNA	mtDNA_chr1	76.279	430	77	17	143683	144102	121566	121152	7.85E-52	206	mtpt18
cpDNA	mtDNA_chr1	76.279	430	77	17	107091	107510	121152	121566	7.85E-52	206	mtpt18
cpDNA	mtDNA_chr1	77.673	318	58	11	144237	144546	38220	37908	1.32E-44	182	mtpt19
cpDNA	mtDNA_chr1	77.673	318	58	11	106647	106956	37908	38220	1.32E-44	182	mtpt19
cpDNA	mtDNA_chr1	77.673	318	58	11	144237	144546	121009	120697	1.32E-44	182	mtpt20
cpDNA	mtDNA_chr1	77.673	318	58	11	106647	106956	120697	121009	1.32E-44	182	mtpt20
cpDNA	mtDNA_chr1	87.582	153	8	7	47948	48089	332554	332706	3.70E-40	167	mtpt21
cpDNA	mtDNA_chr1	85.35	157	12	7	37270	37416	57548	57393	1.04E-35	152	mtpt22

cpDNA	mtDNA_chr1	88.889	126	9	3	71090	71212	164006	164129	3.73E-35	150	mtpt23
cpDNA	mtDNA_chr1	100	70	0	0	113130	113199	297221	297152	4.86E-29	130	mtpt24
cpDNA	mtDNA_chr1	100	70	0	0	137994	138063	297152	297221	4.86E-29	130	mtpt24
cpDNA	mtDNA_chr1	94.937	79	4	0	56243	56321	172737	172659	2.26E-27	124	mtpt25
cpDNA	mtDNA_chr1	94.521	73	4	0	56249	56321	91588	91516	4.89E-24	113	mtpt26
cpDNA	mtDNA_chr1	89.61	77	6	2	91951	92025	243859	243783	4.93E-19	97.1	mtpt27
cpDNA	mtDNA_chr1	89.61	77	6	2	159168	159242	243783	243859	4.93E-19	97.1	mtpt27
cpDNA	mtDNA_chr1	100	30	0	0	8468	8497	332703	332674	8.36E-07	56.5	mtpt28

Table S2. RNA editing sites identified in chloroplast PCGs.

gene	Position	Coverage	Reference	Editing efficiency	Edited number	Alternative
accD	806	28	C	0.8928	25	U
accD	1397	31	C	0.9677	29	U
accD	1415	23	C	1	23	U
atpA	914	379	C	0.9868	374	U
atpA	1148	520	C	0.9096	473	U
atpF	92	322	C	0.9596	309	U
atpI	629	105	C	0.9809	103	U
cemA	98	40	C	0.1999	8	U
clpP	559	235	C	0.9872	232	U
matK	164	100	C	0.53	53	U
matK	457	92	C	0.4456	41	U
matK	634	54	C	0.7962	43	U
matK	704	38	C	0.5263	20	U
matK	1237	49	C	0.6122	29	U
ndhB	149	602	C	0.7508	451	U
ndhB	467	444	C	0.8738	388	U
ndhB	542	392	C	0.8877	347	U
ndhB	586	409	C	0.7603	311	U
ndhB	611	442	C	0.595	261	U
ndhB	737	428	C	0.8247	353	U
ndhB	746	419	C	0.7255	304	U
ndhB	830	212	C	0.8301	175	U
ndhB	836	209	C	0.7607	159	U
ndhB	1255	379	C	0.8205	310	U
ndhB	1481	437	C	0.8901	389	U
ndhC	323	324	C	0.8425	273	U
ndhD	383	136	C	0.9558	130	U
ndhD	674	223	C	0.9372	207	U
ndhD	878	216	C	0.9259	199	U
ndhD	1298	490	C	0.9428	462	U
ndhD	1310	491	C	0.8859	434	U
ndhE	233	57	C	1	57	U
ndhG	50	22	C	0.7727	17	U
ndhH	303	87	C	0.1379	12	U
petB	418	99	C	0.8383	83	U
psaI	83	25	C	1	25	U
psbZ	50	30	C	0.8333	25	U
rpl20	293	28	C	0.8214	23	U
rpl23	74	711	C	0.9915	705	U
rpl23	92	695	C	0.8949	619	U
rpoA	200	664	C	0.9683	639	U
rpoA	830	1223	C	0.6181	756	U
rpoC2	1864	30	C	0.6666	20	U
rpoC2	1893	33	A	0.606	20	U
rpoC2	1913	29	C	0.6206	18	U
rpoC2	1945	25	G	0.6799	17	U
rpoC2	1951	23	C	0.6956	16	U
rps12	221	4016	C	0.8598	3446	U
rps14	80	33	C	0.9393	30	U
rps14	149	39	C	0.8974	35	U
rps18	221	1320	C	0.9409	1242	U

rps2	134	49	C	0.9387	46	U
rps2	248	49	C	0.9183	45	U
ycf2	1571	21	A	0.2857	6	U
ycf2	4627	21	G	0.7142	8	U
ycf2	6657	76	C	0.9736	74	U
ndhB	776	277	A	0.7617	211	G
rpoC2	1866	32	A	0.625	20	G
rpoC2	1867	32	T	0.625	20	G
rpoC2	1872	32	A	0.625	20	G
rpoC2	1902	32	T	0.5937	19	G
rpoC2	1906	32	A	0.5937	19	G
ycf1	4678	208	A	0.1105	11	G
ycf2	6694	73	A	0.9589	70	G
ycf2	6697	71	A	0.9718	69	G
ycf2	6709	70	A	0.9571	67	G
ccsA	279	73	T	0.2191	14	C
rpoA	94	400	T	0.1099	20	C
rpoC2	1861	30	T	0.6666	20	C
rpoC2	1924	28	G	0.6428	18	C
ycf2	4610	21	T	0.6666	14	C
ycf2	6651	77	G	0.948	73	C
clpP	364	137	C	0.3576	49	A
ndhB	777	264	C	0.7386	194	A
petB	308	93	G	0.1827	17	A
psbB	1380	26	G	0.1923	5	A
psbB	1394	20	G	0.3	6	A
psbK	61	124	T	0.1048	7	A
rpoC2	1848	30	T	0.6	18	A
rpoC2	1901	32	G	0.5937	19	A
rpoC2	1933	26	G	0.6538	17	A
rpoC2	1939	25	G	0.6799	17	A
ycf2	4621	22	C	0.409	9	A
ycf2	6685	77	C	0.961	74	A
ycf2	6699	70	T	0.9714	68	A

Table S3. RNA editing sites identified in mitochondrial PCGs.

gene	Position	Coverage	Reference	Editing efficiency	Edited number	Alternative
atp1	15	30	C	0.6	18	U
atp1	1039	26	C	1	26	U
atp1	1110	16	C	0.375	6	U
atp1	1216	30	C	1	30	U
atp1	1292	24	C	0.9166	22	U
atp1	1415	14	C	1	14	U
atp4	106	20	C	0.8	16	U
atp4	203	26	C	0.5384	14	U
atp4	215	22	C	0.6363	12	U
atp4	383	10	C	0.8	8	U
atp4	395	10	C	0.8	8	U
atp4	404	12	C	0.5	6	U
atp6	724	10	C	1	10	U
atp8	76	10	C	1	10	U
atp8	77	10	C	1	10	U
atp8	78	10	A	0.6	6	G
atp9	20	56	C	0.8928	50	U
atp9	50	62	C	0.9032	56	U
atp9	182	30	C	0.8	24	U
atp9	191	30	C	0.7333	22	U
atp9	205	30	C	0.6	18	U
atp9	215	28	C	0.7857	22	U
atp9	223	24	C	0.75	18	U
ccmC	76	14	C	0.4285	6	U
ccmC	161	18	C	0.3333	6	U
ccmC	179	16	C	0.5	8	U
ccmC	184	18	C	0.5555	10	U
ccmC	253	30	C	0.6	18	U
ccmC	276	34	C	0.4117	14	U
ccmC	331	24	C	0.5833	14	U
cob	808	10	C	1	10	U
cob	1084	10	C	1	10	U
cox1	11	18	C	1	18	U
cox1	242	12	C	1	12	U
cox1	443	32	C	1	32	U
cox1	452	34	C	1	34	U
cox1	515	40	C	1	40	U
cox1	551	46	C	1	46	U
cox1	590	34	C	0.9411	32	U
cox1	668	30	C	0.9333	28	U
cox1	715	34	C	1	34	U
cox1	746	36	C	1	36	U
cox1	761	34	C	1	34	U
cox1	1079	12	C	1	12	U
cox1	1405	22	C	0.909	20	U
cox1	1433	22	C	1	22	U
cox1	1499	44	C	0.9545	42	U
cox2	27	18	C	1	18	U
cox2	163	12	C	1	12	U
cox2	253	10	C	1	10	U
cox2	278	12	C	1	12	U

cox2	632	10	C	1	10	U
cox2	715	16	C	1	16	U
cox2	736	14	C	1	14	U
cox3	311	12	C	1	12	U
cox3	314	12	C	1	12	U
cox3	413	22	C	0.909	20	U
cox3	754	12	C	0.6666	8	U
cox3	764	12	C	0.5	6	U
matR	326	10	C	0.6	6	U
matR	413	12	C	0.8333	10	U
matR	1679	32	C	0.875	28	U
matR	1700	24	C	0.9166	22	U
matR	1720	24	C	0.9166	22	U
matR	1734	28	C	0.7857	22	U
matR	1826	10	C	1	10	U
matR	1844	12	C	1	12	U
nad1	287	10	C	0.8	8	U
nad1	400	10	C	0.6	6	U
nad1	508	10	C	1	10	U
nad1	538	12	C	1	12	U
nad1	547	10	C	1	10	U
nad2	56	10	C	1	10	U
nad2	958	12	C	1	12	U
nad2	962	12	C	1	10	U
nad2	1298	16	C	1	16	U
nad2	1400	14	C	1	14	U
nad2	1403	14	C	1	14	U
nad2	1408	14	C	0.4285	6	U
nad2	1416	14	C	0.7142	10	U
nad3	230	10	C	1	10	U
nad4	158	16	C	1	16	U
nad4	164	16	C	1	16	U
nad4	166	16	C	1	16	U
nad4	197	10	C	1	10	U
nad4	362	12	C	1	12	U
nad4	368	12	C	1	12	U
nad4	376	10	C	1	10	U
nad4	403	14	C	0.8571	12	U
nad4	436	14	C	1	14	U
nad4	437	14	C	0.8571	12	U
nad4	449	20	C	0.5	10	U
nad4	451	20	A	0.4	8	C
nad4	452	22	T	0.4545	10	C
nad4	455	22	C	0.3636	8	U
nad4	459	22	G	0.3636	8	C
nad4	461	22	T	0.3636	8	C
nad4	608	12	C	1	12	U
nad4	659	14	C	1	14	U
nad4	767	20	C	0.9	18	U
nad4	896	18	C	1	18	U
nad4	977	24	C	0.8333	20	U
nad4	1006	26	C	0.923	24	U
nad4	1007	28	C	0.9285	26	U
nad4	1009	28	C	0.5	14	U
nad4	1010	28	C	1	28	U

nad4	1129	16	C	0.875	14	U
nad4	1148	12	C	0.8333	10	U
nad4	1172	14	C	1	14	U
nad4	1355	18	C	1	18	U
nad4	1373	20	C	0.8	16	U
nad4	1418	20	C	1	20	U
nad4	1434	20	C	1	20	U
nad5	155	16	C	0.625	10	U
nad5	231	10	T	0.6	6	G
nad5	232	10	G	0.6	6	U
nad5	233	10	G	0.6	6	U
nad5	242	10	C	1	10	U
nad5	272	10	C	1	10	U
nad5	670	14	T	0.4285	6	G
nad5	676	14	C	0.5714	8	U
nad5	713	14	C	0.8571	12	U
nad5	725	14	C	0.8571	12	U
nad5	835	10	C	1	10	U
nad5	1490	16	C	0.875	14	U
nad5	1550	16	C	0.875	14	U
nad5	1568	18	C	0.7777	14	U
nad5	1589	20	C	0.7	14	U
nad5	1610	18	C	0.7777	14	U
nad5	1895	10	C	1	10	U
nad7	244	14	C	1	14	U
nad7	251	14	C	1	14	U
nad7	316	10	C	1	10	U
nad7	335	10	C	1	10	U
nad7	959	84	G	0.8571	72	A
rpl10	101	12	C	0.8333	10	U
rpl10	133	10	C	0.8	8	U
rpl10	134	10	C	0.6	6	U
rpl10	155	10	C	1	10	U
rpl14	222	706	G	0.9858	696	A
rpl14	283	718	C	0.9888	710	G
rpl14	284	714	T	0.9859	704	G
rpl16	104	44	C	0.9545	42	U
rpl16	168	36	C	0.1666	6	U
rpl16	232	32	C	0.9375	30	U
rpl16	335	52	C	1	50	U
rpl16	401	36	C	0.6111	22	U
rpl16	407	34	C	1	34	U
rpl2	40	100	A	0.88	86	G
rpl2	44	102	T	0.9607	98	C
rpl2	45	106	G	0.9811	54	U
rpl2	46	104	A	0.3461	36	G
rpl2	49	108	A	0.9814	96	G
rpl2	78	128	A	0.9843	108	U
rpl2	84	130	T	0.9692	126	C
rpl2	90	136	G	0.1323	18	A
rpl2	91	134	G	0.9701	128	A
rpl2	112	166	T	0.9638	160	G
rpl2	114	172	G	0.1976	34	A
rpl2	118	170	C	0.3647	60	A
rpl2	149	180	G	0.4666	84	A

rpl2	158	202	C	0.4752	96	G
rpl2	165	206	C	0.7669	156	U
rpl2	186	186	T	0.3118	56	C
rpl2	210	158	C	0.7468	118	U
rpl2	215	150	C	1	150	U
rpl2	228	148	C	0.9594	142	U
rpl2	234	136	G	0.25	34	A
rpl2	238	130	T	0.9538	66	A
rpl2	239	128	G	0.5	64	A
rpl2	252	104	G	0.423	44	A
rpl2	259	100	A	0.24	22	C
rpl2	264	90	G	0.8888	80	A
rpl2	265	88	A	0.8863	58	G
rpl2	276	80	C	0.925	74	U
rpl2	278	80	C	0.25	20	U
rpl2	279	76	T	0.3947	30	C
rpl2	282	66	G	0.8787	58	A
rpl2	285	60	G	0.3	18	A
rpl2	291	66	G	0.8787	58	U
rpl2	292	66	A	0.8787	58	C
rpl2	297	68	C	0.8823	58	U
rpl2	309	36	G	0.6111	14	C
rpl2	310	36	A	0.7777	28	C
rpl2	318	38	C	0.3157	12	U
rpl2	321	40	C	0.85	34	U
rpl2	324	40	C	0.85	34	U
rpl2	325	38	C	0.8421	32	G
rpl2	331	56	T	0.8928	50	C
rpl2	334	56	T	0.3571	20	A
rpl2	337	52	T	0.3461	18	G
rpl2	345	56	G	0.5357	30	U
rpl2	348	56	C	0.5	28	U
rpl2	353	64	A	0.3125	20	U
rpl2	369	64	G	0.5312	34	U
rpl2	370	64	G	0.5312	34	U
rpl2	388	60	G	0.9	54	A
rpl2	390	60	A	0.3666	22	G
rpl2	397	60	G	0.5666	34	U
rpl2	401	60	C	0.9	54	G
rpl2	402	60	T	0.9	54	G
rpl2	404	60	A	0.9	54	C
rpl2	423	64	T	0.3125	20	A
rpl2	424	64	A	0.9375	60	U
rpl2	428	64	G	0.9375	60	A
rpl2	439	50	T	0.56	28	C
rpl2	441	50	G	0.28	14	U
rpl2	443	50	C	0.88	44	U
rpl2	448	42	A	0.2857	12	U
rpl2	450	46	C	0.8695	40	U
rpl2	453	42	C	0.3333	14	U
rpl2	455	38	T	0.8947	34	C
rpl2	456	42	G	0.8571	36	A
rpl2	463	40	A	0.85	34	G
rpl2	467	36	G	0.7777	28	A
rpl2	477	20	C	0.7	14	U

rpl2	482	20	A	0.3	6	U
rpl2	488	20	G	0.7	14	C
rpl2	489	20	C	0.7	14	U
rpl2	491	22	C	0.6363	10	U
rpl2	498	12	C	0.8333	10	U
rpl2	501	16	C	0.625	10	A
rpl2	514	16	C	0.375	6	U
rpl2	523	16	C	0.375	6	U
rpl5	512	16	C	0.75	12	U
rpl5	515	16	C	1	16	U
rps10	16	10	C	1	10	U
rps10	193	22	C	0.2727	6	U
rps10	238	20	C	1	20	U
rps10	302	16	C	1	16	U
rps10	331	18	C	1	18	U
rps14	4	26	T	1	26	G
rps14	6	30	G	0.7333	12	C
rps14	9	44	G	1	44	U
rps14	22	60	C	1	60	G
rps14	40	84	T	0.8333	70	C
rps14	45	94	C	0.2127	20	U
rps14	48	92	G	1	92	A
rps14	51	104	T	0.7884	78	G
rps14	56	122	A	1	120	U
rps14	69	160	A	0.9375	150	G
rps14	72	158	G	0.1898	30	A
rps14	87	184	T	1	184	A
rps14	100	188	G	1	188	A
rps14	102	202	T	0.2178	42	C
rps14	123	274	C	0.9854	264	U
rps14	133	284	T	0.9929	282	A
rps14	165	252	C	0.992	250	U
rps14	189	222	T	0.6756	148	C
rps14	194	218	C	1	218	U
rps14	198	206	G	0.6504	132	U
rps14	207	160	T	0.6	96	C
rps14	213	156	C	0.9871	154	U
rps14	220	146	G	0.9863	144	A
rps14	232	140	A	0.3714	52	G
rps14	243	138	C	0.5652	46	U
rps14	249	116	T	0.9655	112	C
rps14	262	28	T	0.8571	24	G
rps14	266	28	G	0.8571	24	A
rps14	271	22	C	1	20	U
rps14	283	14	A	0.8571	12	G
rps14	292	12	T	0.8333	10	G
rps3	92	10	C	1	10	U
rps3	497	10	C	1	10	U
rps3	698	14	C	1	14	U
rps3	875	46	C	0.913	42	U
rps3	1343	34	C	1	34	U
rps3	1534	50	C	0.96	48	U
rps3	1567	52	C	0.923	48	U
rps3	1598	48	C	0.9166	44	U
sdh4	29	10	C	1	10	U

sdh4	39	12	C	0.8333	10	U
sdh4	143	34	C	0.2352	8	U
sdh4	146	34	C	0.2352	8	U
sdh4	154	32	C	0.25	8	U
sdh4	155	28	C	0.7142	18	U
sdh4	203	22	C	0.4545	10	U
sdh4	205	22	A	0.2727	6	G
sdh4	209	22	T	0.2727	6	A
sdh4	222	22	G	0.2727	6	A
sdh4	259	14	C	0.8571	12	U

Table S4. The alignment of plastid *psb*J gene and mitochondrial *psb*J gene.

Location	Coding sequences
Mitochondria	ATGGCCGCTACTACTGAAAGGATTCCTCTTTGGCTGATAGGTACTGTAACTGGTATTCC<br>TGTGATCGGTTCAATAGGTCTTTTCTTTTCCGGTTCATATCCCGGATTGGGTTCATCCTTG<br>TAG
Chloroplast	ATGGCCGATACTACTGGAAGGATTCCTCTTTGGATAATAGGTACGGTAACTGGTATTCC<br>TGTGATCGGTTTAATAGGTATTTTCTTTTATGGTTCATATTCTGGATTGGGTTCGTCCCTC<br>TAA
